# Obligate mutualism within a host drives the extreme specialization of a fig wasp genome

**DOI:** 10.1186/gb-2013-14-12-r141

**Published:** 2013-12-20

**Authors:** Jin-Hua Xiao, Zhen Yue, Ling-Yi Jia, Xin-Hua Yang, Li-Hua Niu, Zhuo Wang, Peng Zhang, Bao-Fa Sun, Shun-Min He, Zi Li, Tuan-Lin Xiong, Wen Xin, Hai-Feng Gu, Bo Wang, John H Werren, Robert W Murphy, David Wheeler, Li-Ming Niu, Guang-Chang Ma, Ting Tang, Sheng-Nan Bian, Ning-Xin Wang, Chun-Yan Yang, Nan Wang, Yue-Guan Fu, Wen-Zhu Li, Soojin V Yi, Xing-Yu Yang, Qing Zhou, Chang-Xin Lu, Chun-Yan Xu, Li-Juan He, Li-Li Yu, Ming Chen, Yuan Zheng, Shao-Wei Wang, Shuang Zhao, Yan-Hong Li, Yang-Yang Yu, Xiao-Ju Qian, Yue Cai, Lian-Le Bian, Shu Zhang, Jun-Yi Wang, Ye Yin, Hui Xiao, Guan-Hong Wang, Hui Yu, Wen-Shan Wu, James M Cook, Jun Wang, Da-Wei Huang

**Affiliations:** 1 Key Laboratory of Zoological Systematics and Evolution, Institute of Zoology, Chinese Academy of Sciences, Beijing 100101, China; 2 BGI-Shenzhen, Shenzhen 518083, China; 3 University of Chinese Academy of Sciences, Beijing 100039, China; 4 College of Plant Protection, Shandong Agricultural University, Tai’an 271018, China; 5 Beijing TransGen Biotech Co. Ltd., Beijing 100192, China; 6 Biology Department, University of Rochester, Rochester, NY 14627, USA; 7 State Key Laboratory of Genetic Resources and Evolution, Kunming Institute of Zoology, Chinese Academy of Sciences, Kunming 650223, China; 8 Department of Natural History, Royal Ontario Museum, 100 Queen’s Park, Toronto, Ontario M5S 2C6, Canada; 9 Environment and Plant Protection Institute, Chinese Academy of Tropical Agricultural Sciences, Danzhou 571737, China; 10 College of Life Science, Hebei University, Baoding 071002, China; 11 School of Biology, Georgia Institute of Technology, Atlanta, GA 30332, USA; 12 Key Laboratory of Plant Resources Conservation and Sustainable Utilization, South China Botanical Garden, Chinese Academy of Sciences, Guangzhou 510650, China; 13 College of Life Sciences, Fujian Normal University, Fuzhou 350108, China; 14 School of Biological Sciences, University of Reading, Berkshire, Reading RG6 6AH, UK; 15 Hawkesbury Institute for the Environment, University of Western Sydney, Locked Bag 1797, Penrith South, DC, NSW 1797, Australia

## Abstract

**Background:**

Fig pollinating wasps form obligate symbioses with their fig hosts. This mutualism arose approximately 75 million years ago. Unlike many other intimate symbioses, which involve vertical transmission of symbionts to host offspring, female fig wasps fly great distances to transfer horizontally between hosts. In contrast, male wasps are wingless and cannot disperse. Symbionts that keep intimate contact with their hosts often show genome reduction, but it is not clear if the wide dispersal of female fig wasps will counteract this general tendency. We sequenced the genome of the fig wasp *Ceratosolen solmsi* to address this question.

**Results:**

The genome size of the fig wasp *C. solmsi* is typical of insects, but has undergone dramatic reductions of gene families involved in environmental sensing and detoxification. The streamlined chemosensory ability reflects the overwhelming importance of females finding trees of their only host species, *Ficus hispida*, during their fleeting adult lives. Despite long-distance dispersal, little need exists for detoxification or environmental protection because fig wasps spend nearly all of their lives inside a largely benign host. Analyses of transcriptomes in females and males at four key life stages reveal that the extreme anatomical sexual dimorphism of fig wasps may result from a strong bias in sex-differential gene expression.

**Conclusions:**

Our comparison of the *C. solmsi* genome with other insects provides new insights into the evolution of obligate mutualism. The draft genome of the fig wasp, and transcriptomic comparisons between both sexes at four different life stages, provide insights into the molecular basis for the extreme anatomical sexual dimorphism of this species.

## Background

In symbiosis, different species live together intimately. Symbiosis is responsible for several major transitions in evolution, including the origin of eukaryotes, and it underpins key ecosystem functions like nitrogen fixation and pollination [1]. In this system, a large species (host) usually interacts with a smaller one (symbiont), which may live inside it as an ‘endosymbiont’. Interactions can be antagonistic, when the symbiont harms the host, such as for parasites and most plant-herbivore interactions [2], or mutualistic, where host and symbiont both benefit from the association and their evolutionary interests are more closely aligned [1,3]. Obligate herbivore-plant mutualisms are relatively uncommon, but the fig pollinating wasp-fig mutualism is an ancient and stable association that originated about 75 million years ago [4].

Typically, endosymbionts are host-specific and show specialized adaptations to life inside their hosts [5,6]. For example, parasites often exhibit a series of morphological reductions. Symbionts may also tend towards gene loss and genome reduction [7]. For example, parasites like tapeworms [8] and the mutualistic bacterium *Buchnera*[9,10] have smaller genomes than their free-living relatives. Many endosymbionts spend most or all of their lives in the host [5,6] and are vertically transmitted from one host generation to the next, often through eggs or propagules [9-11]. Even when an endosymbiont has a free-living stage, it is often quiescent (for example, tapeworm eggs); transmission to other hosts occurs via host contact or through food or water. Consequently, most obligate endosymbionts, benign or parasitic, live most of their lives in somewhat simplified, relatively predictable environments, allowing selective reduction of their anatomies and genomes [5,6].

**Figure 1:**
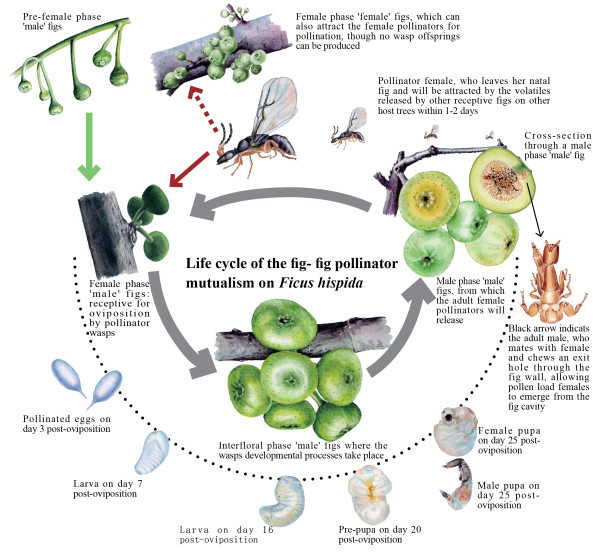
**Life cycle of fig-fig pollinator mutualism on *Ficus hispida*.** Development of the fig pollinator *C. solmsi* is mapped onto the developmental stages of the fig fruit. The fig is dioecious; ‘female’ trees produce fig seeds only, and ‘male’ trees produce fig wasps only.

**Figure 2:**
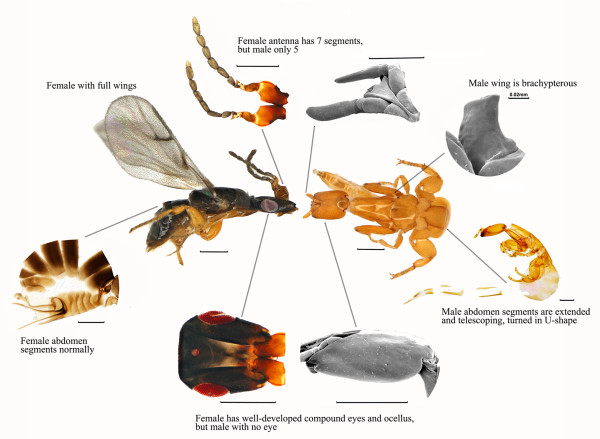
**Extreme morphological dimorphism between female and male fig wasps, *C. solmsi*.** Morphologically, the genders exhibit extreme differences in their compound eyes, wings, antennae, and body color. Scale bar indicates 0.2 mm for each part, except 0.02 mm for male wing.

Fig-pollinating wasps (Agaonidae: Hymenoptera), hereafter referred to as ‘fig wasps’ or ‘fig pollinators’ for brevity, have an obligate, pollinating mutualism with fig trees (*Ficus*: Moracae) [12,13]. Therefore, unlike most other insect herbivores (for example, *Tribolium* beetles, pea aphids, diamondback moths), fig wasps are highly mutualistic with the plants upon which they feed. This is one of the most dramatic and ancient examples of an obligatory herbivore-plant mutualism known. Males spend their entire lives inside figs, but females have a brief (1 to 2 day) free-living adult stage that is crucial because these tiny wasps (2 to 5 mm long) achieve record feats of dispersal to lay eggs in trees up to 160 km away [14,15] (Figure 1). Selection on males favors anatomical and genomic reductions, but selection on females may oppose this. The need of female fig wasps to disperse great distances and precisely locate and enter host figs requires impressive environmental sensing and locomotor abilities. Thus, sex-differential selection results in extreme anatomical sexual dimorphism (Figure 2). Female fig wasps resemble other related wasps, although their narrow heads and detachable antennae are key adaptations for entering figs. In contrast, being wingless, de-pigmented, having reduced antennae and eyes [12,13], males show a series of morphological reductions befitting obligate endosymbionts.

*Ceratosolen solmsi* is the obligate pollinating wasp of *Ficus hispida*, and it has the lifestyle and morphological characters typical of fig pollinators (Figures 1 and 2). We investigate how the longstanding mutualism and largely endosymbiotic lifestyle have shaped the genome of this herbivorous insect by deciphering the draft genome, as well as exploring life-staged transcriptomic differences between both sexes. We also test whether this largely endosymbiotic lifestyle has led to the endosymbiont signature of genome reduction, or if a life cycle with dispersive females prevents this from happening. In the latter case, we predict that male morphological reduction largely reflects reduced gene expression. This draft genome provides a valuable new genome resource for hymenopteran insects, and also permits comparisons with other insects to shed new light on the evolution of obligate mutualism.

## Results and discussion

### Genome assembly and genome size

We sequenced the genome of *C. solmsi* to 92.9× average coverage using shotgun and paired-end sequencing approaches. We obtained 44.6 Gbp of data and estimated a genome size of 294 Mbp based on a k-mer analysis of 12.3 Gbp of high-quality sequences. The genome is spread across 15,018 contigs (contig N50 = 74,395 bp, scaffold N50 = 9,558,897 bp), and assembly results in a 278 Mbp genome (Table 1), which is comparable to sequenced genomes of other insects (most are 200 to 300 Mbp) (summarized in Additional file 1: Table S1). It is about 2.5 times larger than that of human body louse (108 Mbp) [16]. The louse genome has been shown to be unusually small, likely reflecting both extreme host specificity and simple dispersal [16], but we must look to distantly-related insects to find species with contrasting lifestyles. Fig wasps have a similar genome size to other insects in the order Hymenoptera (ants, bees, and wasps) that have different lifestyles, including the honeybee *Apis mellifera*[17] and the parasitoid jewel wasp *Nasonia vitripennis*[18]; fig wasps and parasitoid jewel wasps belong to the same superfamily (Chalcidoidea) within the order.

**Table 1:** **General assembly statistics for the genome of the fig pollinator, *C. solmsi***

**Statistics**	***C. solmsi***
Contigs (*n*)	15,018 (length ≥100 bp)
Average length of contigs	18,421 bp
Max contig length	683,425 bp
Total length of contigs	276,647,649 bp (length ≥ 100 bp)
Contig size N50	74,395 bp
Scaffolds (*n*)	7,397 (length ≥ 100 bp)
Average length of scaffolds	37,575 bp
Max scaffold length	27,400,720 bp
Total length of scaffolds	277,939,842 bp (length ≥ 100 bp)
Scaffold size N50	9,558,897 bp
Total coverage	94.52% (estimated size 294,060,873 bp)
Predicted genes	11,412

### Assessment of genome assembly and annotation

Several analyses serve to infer the accuracy and completeness of the genome sequence of *C. solmsi*. Gene coverage of *C. solmsi* and some other insect genomes were assessed against 248 core eukaryotic genes. The coverage rates for assembly and the gene-set of *C. solmsi* are 100% and >88%, respectively, using CEGMA 2.4 [19]. These rates are comparable to other insects (Additional file 1: Tables S2 and S3). Analyses involving our independently sequenced and assembled *C. solmsi* transcriptome and EST datasets (unpublished EST data from our lab) find that all assemblies cover most of the gene regions (Additional file 1: Table S4).

### Repetitive DNA and non-coding RNA

Fig wasps have one of the most AT-rich (69.6%) insect genomes sequenced to date. The genome contains only 27.4 Mbp of repetitive sequences and only 6.4% transposable elements (TEs) (details in Additional file 1: Tables S1 and S5). Consequently, fig wasps have far less repetitive DNA than jewel wasps, which have among the highest abundance of transposable elements in insects [18], and ants [20]. Nevertheless, fig wasps are quite similar to honeybees in having few repetitive sequences [17]. Our annotation also includes 64 microRNAs, 138 tRNAs, 39 rRNAs, and 19 small nuclear RNAs in the genome of *C. solmsi* (Additional file 1: Table S6).

### Gene annotations, comparative genomics, and natural selection of genes

**Figure 3:**
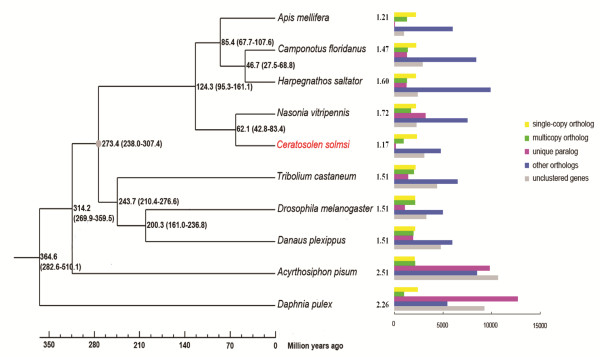
**Phylogenetic relationships and gene-family clusters of 10 species of arthropods.** Gray dot (for calibration) represents the divergence time of 307.4 to 238.0 million years ago between *Drosophila melanogaster* and *Apis mellifera* based on fossil evidence. Numbers following each species indicate the average numbers of genes per gene family. Single-copy orthologs have only one copy in each species, multicopy orthologs have more than one copy in different species, unique paralogs include the species-specific, other orthologs are unclassified orthologs, and unclustered genes cannot be clustered into known gene families.

**Figure 4:**
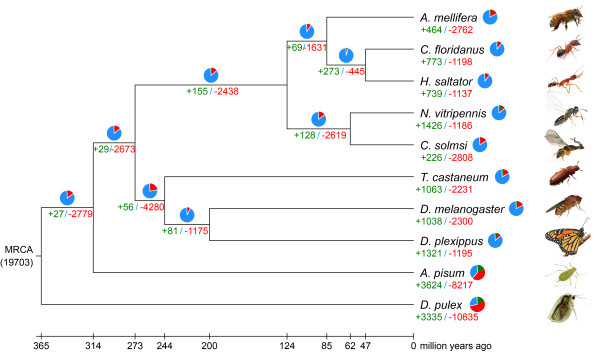
**Gene family contraction and expansion in 10 arthropod species.** Green indicates expansions, red denotes contractions, and blue signposts no obvious change. Compared to the other species, the *C. solmsi* has the smallest number of expanded gene families.

The gene set of *C. solmsi* consists of only 11,412 protein-coding genes based on a combination of *ab initio*, EST-based, and sequence similarity-based methods. EST or RNA-seq analyses support the occurrence of more than 90% of the genes (see below; Additional file 1: Table S4). Compared with other insects, *C. solmsi* has fewer unique paralogs and annotated genes (Figure 3). Overall, the genome has many contracted gene families, yet we detect expansion of only a few families. This pattern differs from other insects. Among the studied insect species, the *C. solmsi* has the lowest ratio of contracted gene families to expanded ones, with 2,808 contracted families comprising only 466 genes and only 226 expanded families comprising 704 genes (Figure 4). The expanded families mainly involve brain morphogenesis, startle response, locomotion involved in locomotory behavior, and neuromuscular processes, all of which may be associated with the fig wasps’ refined behaviors of host localization, oviposition, and mating (Additional file 1: Table S7). Contracted gene families involve various cellular and metabolic processes (Additional file 1: Figure S1). Interestingly, genes unique to fig wasps mainly involve the remodeling of chromatin structure, which is often subjected to epigenetic regulation of gene expression [21,22] (Additional file 1: Figure S2).

Phylogenetic analysis based on all single copy orthologous genes provides a good resolution of the phylogenetic relationship among nine sequenced insect genomes. It also dates the divergence of the jewel wasps and fig wasps to approximately 42.8 to 83.4 million years ago (Figure 3). This date is consistent with previous inferences about the origin of fig and pollinator mutualism [4].

The fig wasp genome has a higher overall Ka/Ks ratio than other insects (mean omega values: *C. solmsi*, 0.039; *Tribolium castaneum*, 0.004; *N. vitripennis*, 0.026; *A. mellifera*, 0.028; *Camponotus floridanus*, 0.029; paired Wilcoxon rank sum tests: *P* <0.0001) (Additional file 1: Figure S3), indicating its faster proteins evolution. All the 157 genes with Ka/Ks > 1 along the branch leading to *C. solmsi* are analyzed further using the codon-based branch-site tests implemented in PAML [23], and 13 genes are identified as obviously rapidly evolved, which may be due to positive or relaxed selection (functional annotations of these 13 genes are provided in Additional file 1: Table S8). Eight of these 13 genes either encode transmembrane proteins, or are transmembrane protein-associated; these often act as transporters of signals or substances and are expected to evolve rapidly. For example, CSO_001764 may encode a hippocampus abundant transcript 1 protein, a sugar transporter [24], while CSO_006481 is a lipid storage droplets surface-binding protein 1-like gene involved in the activation of lipolysis [25]. Interestingly, two positively selected genes, CSO_003961 (rac GTPase-activating protein 1-like) and CSO_005676 (guanine nucleotide exchange factor 2), act together yet contrarily to control the activity of G proteins [26].

### Extreme contraction of gene repertoires in the chemosensory toolbox

In the remarkable lifecycle of the fig wasp (Figure 1), a fig wasp plays a much more active role than immovable fig trees in the choosing of an appropriate host, though the figs can also ‘actively’ release some specific chemical signals to attract fig wasps when they are receptive [27] (Figure 1). Female fig wasps may search over long distances for the only fig species in which they can reproduce, even though tropical forests often have tens of species of *Ficus*. In addition, fig wasps must rapidly locate a chemically signaling host within their short free-living stage [14] (Figure 1). To understand the gene composition of the chemosensory toolbox of fig wasps, we examine five gene families that mediate detection of chemical cues [28]: gustatory (Gr), olfactory (Or), and ionotropic receptors (Ir); odorant binding proteins (OBPs); and chemosensory proteins (CSPs) (Table 2).

**Table 2:** Comprehensive list of chemosensory system genes among insects

	***C. solmsi***	***N. vitripennis***	***A. mellifera***	***S. invicta***	***A. pisum***	***T. castaneum***	***P. xylostella***	***P. humanus***
Gr	6 (1)	58 (11)	13 (3)	NA	77 (2)	215 (25)	26	6
Or	46 (2)	301 (75)	174 (1)	297	79 (10)	307 (42)	83	10
Ir	11	10 (1)	9	NA	11	23	49	10
OBP	7	90	21	18	15 (1)	50 (1)	64	5 (1)
CSP	7	9	6	14	11 (1)	20 (1)	20	7 (1)

Comparative data from references [2,16,31,32,90-92].

Pseudogenes are in parentheses.

*A. mellifera*: *Apis mellifera*; *A. pisum*: *Acyrthosiphon pisum*; *C. solmsi*: *Ceratosolen solmsi*; CSP*,* chemosensory protein; Gr*,* gustatory receptor; Ir*,* ionotropic receptors; *N. vitripennis*: *Nasonia vitripennis*; NA*,* not applicable; OBP*,* odorant binding protein; *Or*, olfactory receptor; *P. humanus*: *Pediculus humanus; P. xylostella*: *Plutella xylostella*; *S. invicta*: *Solenopsis invicta*; *T. castaneum*: *Tribolium castaneum*.

Fig wasps have only five Gr genes, the smallest repertoire known in insects, and one less than in human body lice, which have been permanent parasites for the past 5 to 7 million years [16]. Among the Gr genes, two orthologs of sugar receptors, CsGr1 and CsGr2 that help insects acquire nutrition, are conserved in other insects. Although CsGr3 and CsGr4 are conserved along with the gene AmGr7 in the honeybee, they are expanded in the genome of the jewel wasp (NvGr48-58). Lineages CsGr4 and NvGr48-50 are chalcidoid-specific (Additional file 1: Figure S4). No orthologs occur for the otherwise conserved carbon dioxide and the bitter receptors Gr genes. Interestingly, transcriptomic data and PCR experiments confirm the presence of one pseudogene (CsGr5PSE), which encodes a highly conserved Gr in all other insects (for example, NvGr3 and DmGr43a, Additional file 1: Figure S4). The gene appears to generally be involve in sensing fructose, and in *Drosophila*, it is also an important brain fructose receptor to sense hemolymph fructose and promote feeding in hungry flies but suppress feeding in satiated ones [29]. The pseudogenization of this gene in fig wasps is unusual. We also detect six other pseudogenes distributed on different scaffolds, each of which contains a short fragment similar to part of a gustatory receptor gene only. These might be remnants of gene losses.

Fig wasps have 46 Ors (with two pseudogenes), compared to 301 in jewel wasps and 174 in honeybees, and they lack fig wasp-specific Or subfamilies (Additional file 1: Figure S5). Similarly, jewel wasps have 90 OBPs, while honeybees (with 21) and fire ants (with 18) have far fewer [30], and fig wasps have just seven. The contrast with other herbivorous insects is also striking. For example, *Tribolium* beetles have 307 Ors and 50 OBPs [31] and the diamondback moth has 83 Ors and 64 OBPs [2]. The extreme contraction of Or and OBP diversity in fig wasps is convergent with the case of the human body louse [16], possibly reflecting the common theme of extreme host-specificity.

Fig wasp Gr and Or genes, but not those of jewel wasps or honeybees, appear to be under more relaxed selection as they show a higher ka/ks ratio than single copy orthologous genes (Additional file 1: Table S9). Consistent with the repertoire reductions, this may be associated with the process of host specialization [32].

Genes involved in the chemosensory system have been suggested to be involved in the evolution of host specialization in some insects, such as pea aphids [33] and *Drosophila sechellia*[34,35]. In fig wasps, sensing other distracting aspects of the environment is probably of trivial consequence, and perhaps it reduces the success of finding a new host. Thus, the Gr, Or, and OBP families in fig wasps appear to have experienced dramatic contractions relative to other insects. Though fig wasps have few Or genes, they still have far more than lice [16]. This may reflect more complex mate-searching, as male fig wasps must locate mates inside galled flowers in the dark cavity of a fig that often contains other species of fig wasps, including abundant and sympatric non-pollinating wasps (Figure 1).

### Reduced system of detoxification

In insects, three major groups of enzymes have important functions in the processing of environmental chemicals: glutathione-S-transferases (GSTs), cytochrome P450s (P450s), and carboxylesterases (CCEs). These enzymes play the major roles in disarming toxic xenobiotics [36]; the P450s and CCEs are also used for clearing signals related to the reception of kairomones and pheromones [37,38]. Most insects have similar numbers of genes involved in detoxification [18], except for the honeybee, which has far fewer genes associated with xenobiotic metabolism. Reductions in the honeybee may be related to its specialized eusocial behavior and homeostasis of the nest environment [17]. In the obligate fig-fig pollinator mutualism, the highly specialized ecology and strict host specificity of fig wasps may obviate the need for many detoxification genes.

*C. solmsi* has only 11 cytosolic GST genes, far fewer than the 19 to 37 of other herbivorous arthropods but similar to the eight of honeybees (Additional file 1: Table S10). Honeybees have the smallest number of P450 genes (with 46) among previously characterized insect genomes. However, the fig wasp has only 34 P450 genes (Additional file 1: Table S10), which cluster according to sequence similarity into mitochondrial CYP, CYP2, CYP3, and CYP4 groups. The dramatic difference in gene members is not due to the CYP2 or CYP mitochondrial groups, which are conserved across different insects, but mainly due to the CYP3 and CYP4 clans, which are important gene members involved in xenobiotic metabolism [39]. For example, the fig wasp has a significant reduction to only 11 basic members in CYP3 clan (Additional file 1: Table S10 and Figure S6). In addition, the genome of *C. solmsi* has only one-third as many genes in the CYP4 clan as *N. vitripennis*.

The fig wasp genome has 17 CCE sequences, again the smallest number in any of the insect genomes characterized so far (Additional file 1: Table S10). These genes occur in three major functional classes: dietary/detoxification; hormone/semiochemical processing; and neuro-developmental/cell adhesion [39]. Fig wasps have 11 genes in neuro-developmental/cell adhesion, and the fig wasp, jewel wasp, and honeybee (all hymenopterans) have the same number of genes in each of the six clades in this class. This indicates an ancient origin of these lineages [39] and suggests that these genes may play vital roles beyond detoxification; they may act as cell adhesion molecules in the development of the nervous system [40,41]. The other two classes are extremely under-represented in the fig wasp genome. For example, the fig wasp genome contains only four members in the clade of dietary/detoxification genes, and only two members in the clade of hormone/semiochemical processing - hymenopteran b-esterases and juvenile hormone esterases.

Most herbivorous insects have an antagonistic relationship with their host plants [2], but mutualistic fig wasps develop inside benign hosts. The fig acts as a ‘nursery’ for developing fig wasp larvae, each of which consumes one presumptive seed (Figure 1). Thus, figs (and galls within figs) provide a relatively safe nursery by insulating developing larvae from the external environment and many antagonists. In tune with this, the fig wasp genome shows a marked reduction of its detoxification system relative to other insects. It has not only the fewest cytochrome P450 and carboxylesterase genes of nine arthropod genomes compared, but also far fewer glutathione-S-transferases genes than other insect species.

### Reduced cuticular protein genes mainly expressed in late pupa stage

A group of cuticular proteins specific to arthropods (hereafter called CPR), characterized by an extended version of the Rebers and Riddiford (R&R) consensus, a conserved chitin-binding domain of about 63 amino acids [42], have previously been studied in many insect species and the results indicate that hymenopterans have far fewer CPR genes than dipterans and lepidopterans. The relatively high level of protection afforded for the development of these hymenopteran larvae may account for the trend [18].

**Figure 5:**
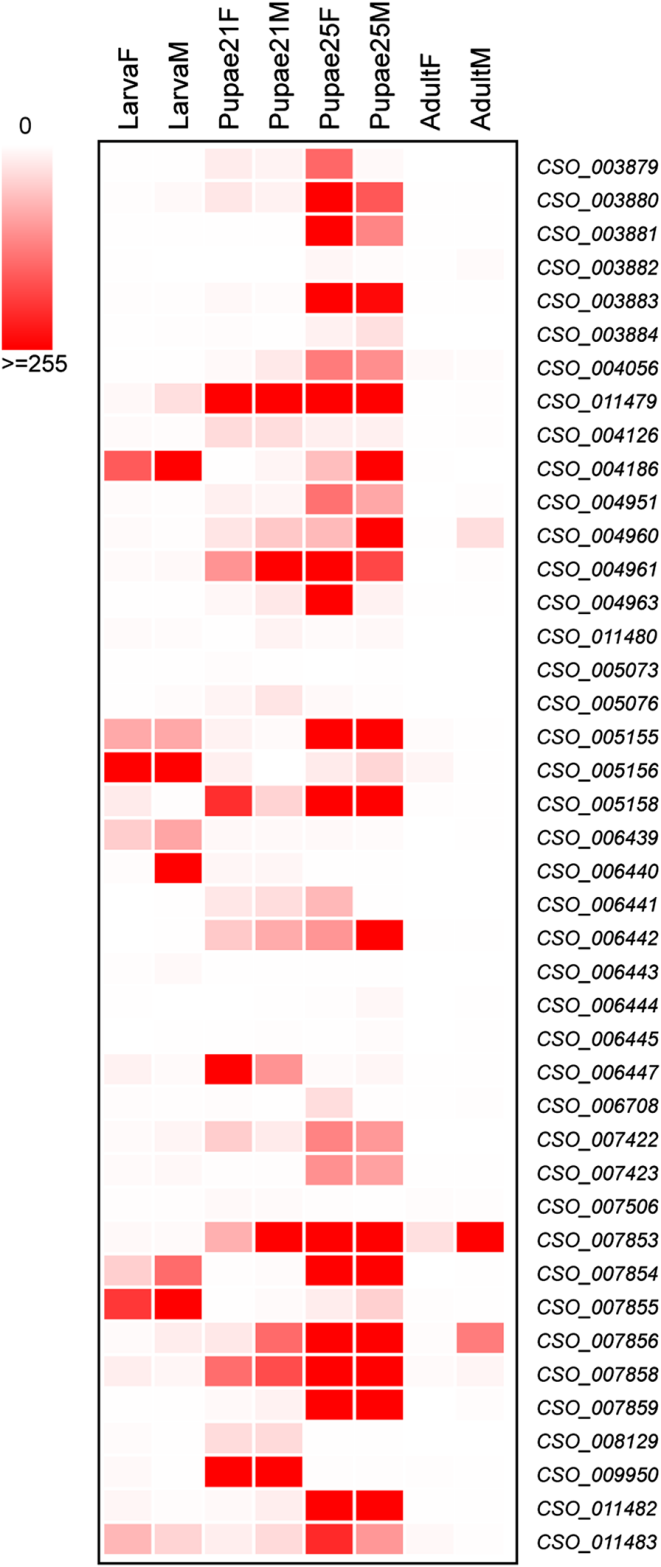
**Heatmap indicating the CPR gene expression through different life stages of fig wasps, *C. solmsi*.** Most of the genes are highly expressed in late pupa stage. The bar indicates expression level ranging from zero to higher. LarvaF: female at larva stage; LarvaM: male at larva stage; Pupae21F: female at early pupa stage (the 21st day after oviposition); Pupae21M: male at early pupa stage (the 21st day after oviposition); Pupae25F: female at late pupa stage (the 25th day after oviposition); Pupae25M: male at late pupa stage (the 25th day after oviposition); AdultF: female at adult stage; AdultM: male at adult stage.

Throughout development, fig wasp larvae shelter in safer places than jewel wasps and honeybees, considering the double safeguards of the fig and their galls within the fig. Thus, we predict fewer CPR genes will occur in fig wasps than in jewel wasps and honeybees. The fig wasp genome has 42 CPR genes, less than *N. vitripennis* (with 62 genes), but a few more than *A. mellifera* (with 35 genes). Among four different life stages of fig wasps (larva, early pupa, late pupa, and adult; details in Methods), most genes have the highest expression in pupae, especially the late pupa stage, and only eight genes have highest expression in larvae (Figure 5). These expression patterns further suggest that the protected environment in which larval fig wasps develop drives the reduction of CPR genes; the larva stage express very few CPR genes and most of the remaining genes are expressed in the late pupa stage when the chitin skeleton is formed.

### Gene members in innate immune response

Insects have effective immune responses in defense of their exposure to a variety of infections, including bacteria, fungi, viruses, and parasites. The responses involve clotting, phagocytosis, encapsulation, and the production of antimicrobial proteins, with the activation of four signaling pathways: Toll, immunodeficiency (IMD), c-Jun N-terminal kinase (JNK), and Janus kinase/Signal transducers and activators of transcription (JAK/STAT) [43,44].

**Figure 6:**
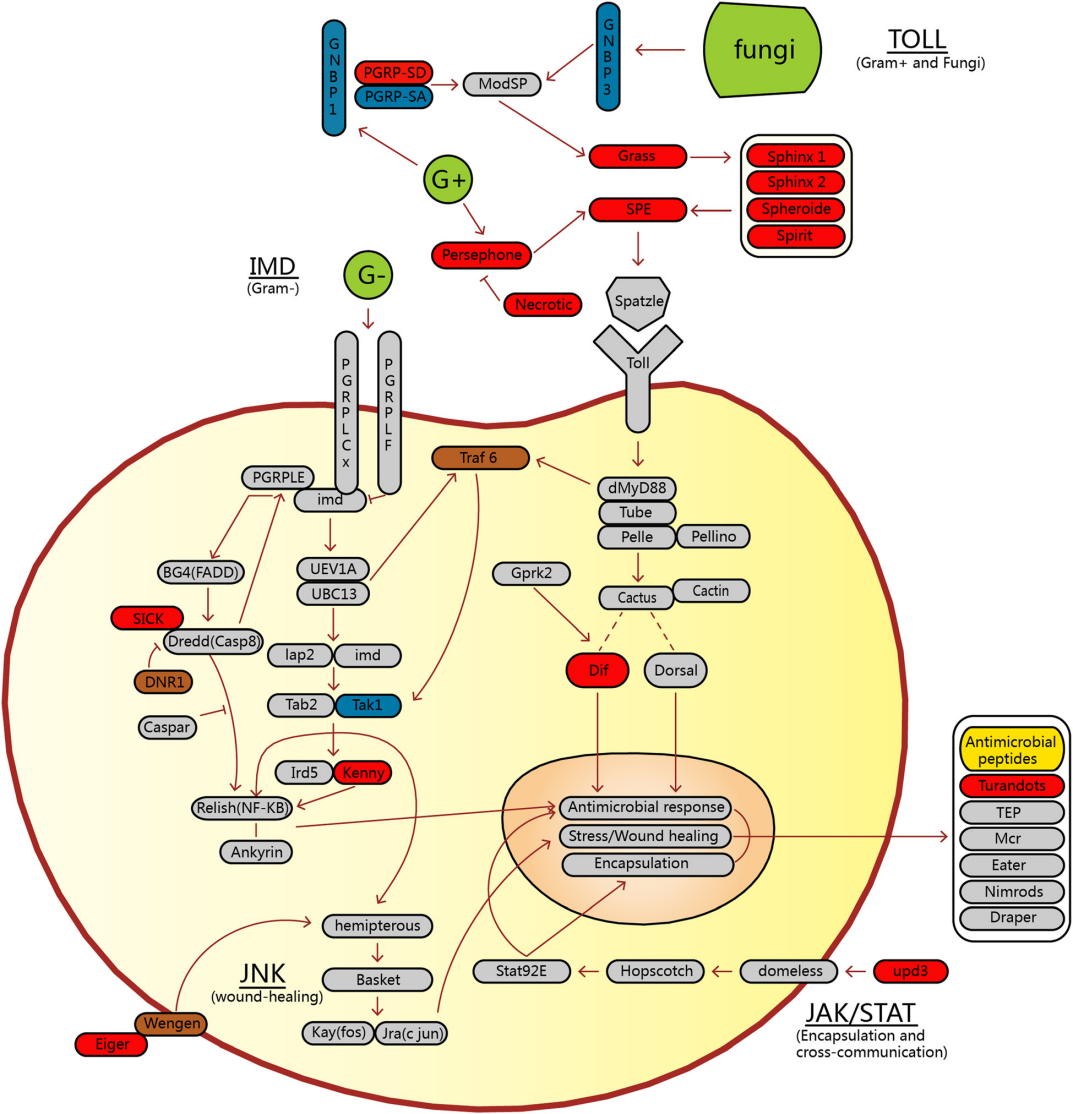
**Four main immunity pathways in *Drosophila melanogaster *and their counterparts in three hymenopteran species.** The four main immunity pathways are Toll, immunodeficiency (IMD), c-Jun N-terminal kinase (JNK), and Janus kinase/Signal transducers and activators of transcription (JAK/STAT). The hymenopterans are *Apis mellifera*, *Nasonia vitripennis*, and *C. solmsi*. Gray indicates genes occurring in all four species. Red indicates genes described from *D. melanogaster* but absent in all hymenopterans. Brown indicates genes absent in the chalcid *N. vitripennis* and *C. solmsi*, but present in *D. melanogaster* and *A. mellifera*. Blue shows genes absent in *C. solmsi* only. Green signposts outside infections such as Gram-positive and Gram-negative bacteria, and fungi. Yellow denotes antimicrobial peptides (details in Additional file 1: Table S11).

Fig wasps may live in sheltered gall-environments with few bacteria, yet, like *Drosophila*, they are at great risk of attack by parasitoids. Thus, we compare immune pathway models of the annotated draft genome with those of honeybees, jewel wasps, and four other insects (Additional file 1: Table S11). We manually annotate 89 genes involved in humoral immune actions that contribute to pathogen recognition, signaling, and response (Figure 6; Additional file 1: Table S11). Like *Drosophila*, the fig wasps appear to conserve key components of the four signaling pathways, except for the absence of *traf6* and *dif* in Toll signaling, and *sick*, *dnr1*, *tak1*, and *kenny* in IMD signaling. Except for *tak1*, these are also absent in *N. vitripennis*, indicating a lineage-specific characteristic of chalcids. Fig wasps do not have orthologs of *grass*, *necrotic*, *persephone*, *SPE*, *spheroide*, *sphinx1*, *sphinx2*, *spirit*, and *PGRP-SD* that encode proteins involved in the recognition of Gram-positive bacteria and fungi. This consistency with honeybees and jewel wasps suggests a Hymenoptera-specific mechanism for stimulating Toll signaling by Gram-positive bacteria and fungi. Genes that can trigger JAK/STAT (*upd3*) or JNK (*eiger*, *wengen*) signaling are absent in the chalcid genomes. The fig wasp genome has nearly intact gene members in the cellular response process, but is missing three genes: *turandots*, *hemese*, and *rac*. It also has very few genes encoding antimicrobial peptides (AMPs) (Additional file 1: Table S11). Whereas *D. melanogaster* has 20 AMP genes, the fig wasp has only eight, and both species have distinct gene-member composition. Chalcidoid *N. vitripennis* has 37 AMP genes [45]. Thus, each species of insect, even those that are fairly closely related, has distinct gene repertoires encoding antimicrobial peptides.

### Sex-specific gene expression and extreme anatomical sexual dimorphism

Male fig wasps live within the fig for their entire lives and females for about 98% of theirs (Figure 1). The genders exhibit extreme intraspecific morphological divergence in the compound eyes, wings, antennae, body color, and size, as expected given their functional adaptations to different life histories (Figure 2). Females must emerge from syconia and fly to another fig tree to oviposit and pollinate; they need fully developed compound eyes (with ocelli), wings, and antennae to do so. In contrast, males have no compound eyes (ocelli absent), vestigial wings, and shorter antennae because these characters are advantageous for living inside syconia.

**Figure 7:**
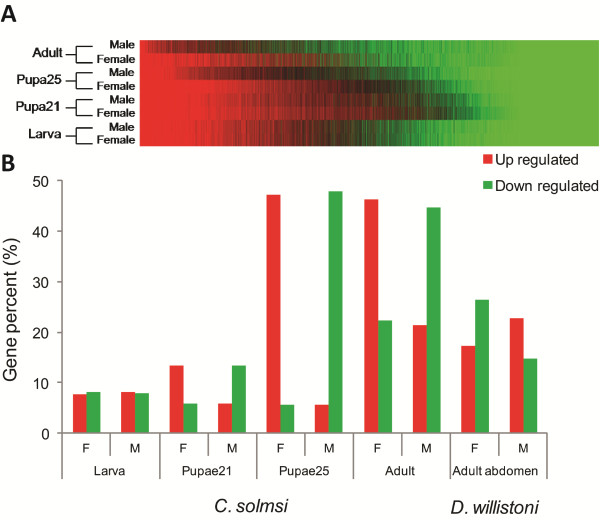
**Gene expression profiles for female and male fig pollinators at four key life stages. (A)** Gene expression profiles with highly expressed genes shown in red, moderately expressed genes in black, and low or unexpressed genes in green. **(B)** Comparisons of the gene numbers with significantly diverged expression between both genders in the four stages of fig wasp (data from abdomen of *Drosophila willistoni* as control); red columns indicate the percentages of upregulated genes and green columns show the percentages of downregulated genes. Figure 5 provides descriptions of the samples.

Our manual annotation fails to discover significant divergences between the fig wasp and other insects in genes potentially involved in the development of compound eyes and wings (Additional file, section of ‘development of compound eyes and wings’). The extreme sexual dimorphism of fig wasps on morphological and other biological characters argues against genome simplification. However, a comparison of transcriptomes discovers sex-differential gene expression at the life-stages of larva, early pupa, late pupa, and adults (Figure 7). Approximately 53% of annotated genes show sex-differential expression in pupae and 67% in adults (Figure 7; Additional file 1: Table S12). Typically, reduced expression in males is most evident in late pupae (48.0% downregulated in males *vs*. 5.5% in females) and adults. These findings are consistent with the hypothesis that males use a much reduced gene repertoire. In contrast, free-living insects (for example, *Drosophila willistoni*) have lower percentages of sex-biased expressed genes (37.6% to 43.6%) and no distinct sex bias occurs for the over-expressed gene ratio (17.2% in females and 22.8% in males) (Additional file 1: Table S12). Consequently, this strong bias in sex-differential gene expression presumably underlies the dramatic sexual dimorphism of fig wasps.

Analyses of GO functional enrichment among genes divergently expressed in females and males provide critical insights into sexual dimorphism (Additional file 1: Table S13). Larvae do not have significantly enriched divergent genes, except for some upregulation of genes involved in oxidoreductase activity in males. Few genes are divergently expressed at early pupa stage, when upregulated male genes are enriched in GO functions of catalytic and oxidoreductase activities, and downregulated in channel, receptor, and transmembrane transporter activities. At late pupa and adult stages, expressed genes are distinctly sex-biased and the distributions of up- and downregulated male genes differ. Very few genes are upregulated in late pupa stage and no significant enrichment is detected. Upregulated, enriched genes of adults involve transporter activity functions. Downregulated genes in late pupa and adult stage males are significantly enriched in the structural constitution of ribosomes, and many functions involved in gene translation. Thus, the sexual divergence of gene expression results in female and male morphological dimorphism.

### Screening for Horizontal Gene Transfer (HGT)

Many recent studies report the horizontal transfer of genes between bacteria and animals and this may be important source of evolutionary innovation [46]. Given the tight association of fig wasp with its microbes such as *Wolbachia*[47], and the long co-evolutionary history with fig trees, fig wasps may have acquired genes from their microbes, or even from fig trees.

Our exhaustive search for genes of bacteria, fungi, viruses, and plants in the genome of fig wasp fails to detect any from either fungi or plants. However, some gene fragments appear to have apparent bacterial or viral origins. A *blastp* search of the all-protein sequences database detects transferred genes encoding RNA-dependent RNA polymerase (CSO_001922) and ANK repeat protein (CSO_009275). Both genes are intact without introns. CSO_001922 may have been transferred from a virus and CSO_009275, which also occurs in *N. vitripennis*, appears to be a recent transfer from *Wolbachia* (Additional file 1: Figures S7 and S8). Thus, fig wasps appear to have acquired a few functional gene fragments and intact genes via lateral gene transfer from bacteria and viruses.

The sequenced fig wasp species is uninfected with *Wolbachia*, which facilitates our detection of lateral gene transfers from *Wolbachia* by reducing the risk of contamination. A further *blastn* search of the fig wasp genome against the whole bacteria database detects 12 small fragments putatively recently acquired from bacteria; their lengths range from 105 bp to 1,509 bp (Additional file 1: Table S14). PCR amplification of the sequences spanning the transferred fragment plus its flanking region confirms one candidate fragment in scaffold 108 that is similar to UDP-N-acetylmuramoylalanine-D-glutamate ligase of *Wolbachia* endosymbiont *wVitB*, although it cannot be translated due to a premature stop codon. This fragment may represent an ancient transfer event in an ancestor of *C. solmsi* infected with *Wolbachia*, or it may indicate that *Wolbachia* previously infected *C. solmsi*, but not now. The transferred fragments and frequency of laterally transferred genes events in the genomes of *N. vitripennis* and *C. solmsi* differ substantially, indicating that many of these transfer events happened independently and relatively recently in different lineages [18].

## Conclusions

The fig-fig wasp system is one of the most dramatic and ancient examples of an obligatory herbivore-plant mutualism known. Both figs and fig wasps show morphologies highly specialized to this mutualism. The first draft genome sequence of a fig wasp, that of *C. solmsi*, reveals how the long-term mutualism has shaped its genome. The genome generally resembles that of other insects in that it has similar gene content to other hymenopterans and other insects in genes such as those encoding for heat shock proteins, circadian rhythm, yellow and royal jelly-like proteins, hox complex, sex determination, and DNA methylation (for further information on these analyses plus an interesting analysis on the nutritional and microbial in the genome, please see Additional file 1). However, importantly, the fig wasp genome shows marked reductions of genes associated with environmental sensing and protection (for example, detoxification). Extreme host-specificity and endosymbiosis in a mutualistic host appears to drive this pattern. Counter-intuitively, complex dispersal is viable with a streamlined chemosensory toolbox, maybe because overwhelming selection pressure involves homing in on the correct host during the short adult lives of females; the sensing of other aspects of a complex external environment is of little importance, and might even be distracting. Comparisons of transcriptomes between female and male fig wasps at the key life stages of larva, early pupa, late pupa, and adult indicate that extreme anatomical sexual dimorphism likely results from a strong bias in sex-specific gene expression. Our analyses facilitate a deeper understanding of the biology of fig wasps, and also provide new insights into the evolution and genomic adaptations of mutualisms.

## Materials and methods

### Materials

We chose three trees of *Ficus hispida* in Danzhou (19° 30’ N, 109° 31’ E), Hainan province, China for inoculation of the fig pollinator species (*Ceratosolen solmsi*). Samples for genomic DNA extraction were collected from June to August in 2010. In each inoculation experiment, naturally growing figs were covered with mesh bags from their very early developmental stages to exclude all insects, including fig wasps. One mated female (‘mother’) fig wasp was introduced into each bagged fig to lay eggs. About 1 month later, when the offspring were mature, we transferred several mated female daughter fig wasps into other bagged figs at their receptive stages. Following this second generation of development, we collected all male grandson fig wasps for genome sequencing. In fig wasps, like other hymenopterans, males are haploid and provide better targets than diploid females for genome sequencing projects. These processes reduced genomic heterozygosity and, thus, improved the quality of assembly. After thoroughly washing with double-distilled water, we immediately froze the samples in liquid nitrogen and transported them to the laboratory on dry ice. DNA extraction occurred immediately on arrival.

The following groups of samples were selected for transcriptome analyses: (1) larval female-16th day (larva), (2) larval male-16th day (larva), (3) pupal female-21th day (early pupa), (4) pupal male-21th day (early pupa), (5) pupal female-25th day (late pupa), (6) pupal male-25th day (late pupa), (7) adult female-29th day, and (8) adult male-29th day. The days indicate time since the eggs were laid. For sample groups 1 to 6, we used only one fig wasp for each RNA extraction. For each adult group, we used 50 individuals. All RNA extractions occurred immediately after collection.

### DNA extraction

About 500 male fig wasps were divided into 10 samples of 50 individuals each and used for DNA extraction using a method modified from the protocol developed by J. Rehm, in the Berkeley *Drosophila* Genome Project [48]. Briefly, 50 fig wasps were completely homogenized in 400 μL Buffer A (100 mM Tris–HCl, pH 7.5; 500 mM EDTA; 100 mM NaCl; 0.5% SDS). A total of 3 μL RNaseA was added to the homogenate, followed by 2 h incubation at 37°C. Then 3 μL Proteinase K was added to the mixture, followed by 2 h incubation at 58°C. Next, 800 μL of LiCL/KAc solution (5 M KAc and 6 M LiCl) was added before the tube was incubated on ice for 10 min. Then the mixture was centrifuged at 14,000 g for 15 min at 4°C, and 1 mL of supernatant was transferred into a new 2 mL tube. To precipitate the genomic DNA from the supernatant, 0.8 mL ice isopropanol was added, and centrifuged at 14,000 g for 15 min at 4°C. The supernatant was then aspirated. The DNA pellet was washed with 70% ethanol, followed by drying for 5 min. The DNA was dissolved in 50 μL of TE buffer and stored at −80°C.

### RNA extraction

Total RNA was isolated using the RNeasy® Micro Kit (Qiagen, Shanghai, China) and treated with DNase (Qiagen, Shanghai, China). A NanoDrop ND-1000 Spectrophotometer (Nano-Drop Technologies, Wilmington, DE, USA) was used to confirm adequate RNA concentration and A260/A280 ratio. RNA was dissolved in 20 μL RNase-free water and kept at −80°C. Larval females and males that had no distinct morphological divergence were discriminated by the variable splicing pattern of the sex determination gene *doublesex*[49]. The procedure used 50 ng dissolved RNA of larva fig wasp to synthesize first-strand cDNA by priming with oligo(dT) with TransScript® II First-Strand cDNA Synthesis SuperMix (TransGen Biotech, Beijing, China). The sex of larva individual was then confirmed by PCR of the male-specific splice isoform of *doublesex*.

### Shotgun libraries construction and sequencing

Genomic DNA was sheared into fragments and seven libraries were constructed with inserted fragment sizes ranging from 200 bp, 500 bp, 800 bp, 2 kb, 5 kb, and 10 kb to 20 kb by the manufacturer’s library kit (Illumina) [50]. A PCR-free library was also constructed. The libraries were sequenced using the Illumina-HiSeq™ 2000 platform with paired-end sequencing approaches.

For RNA-seq, beads with oligo(dT) were used to isolate poly(A) mRNA. Fragmentation buffer was then added for cutting mRNA into short fragments, which were used as templates. Random hexamer primers were used to synthesize first-strand cDNA. Second-strand cDNA was synthesized using a mixture of buffer, dNTPs, RNase H, and DNA polymerase I. Short fragments were purified with QiaQuick PCR extraction kits and resolved with EB buffer for end repair and addition of poly(A). Next, the short fragments were connected with sequencing adaptors. For amplification with PCR, we selected suitable fragments as templates based on agarose gel electrophoresis. Finally, the libraries were sequenced using an Illumina HiSeq™ 2000. RNA-seq for abdomen of adult *Drosophila willistoni* was downloaded and compared between female and male [51].

### Genome assembly

We used SOAPdenovo (version 2.01) to assemble the genome with the following procedures (basic information in Table 1; details are referred to the giant panda [50]):

(1) construct contig: split the short-insert size library data into 43-mers and construct a de Bruijn graph. Next, obtain parameters from a simplified graph. Finally, connect the 43-mer path to get contigs;

(2) construct scaffold: realign all usable reads onto contig sequences, then calculate the amount of shared paired-end relationships between each pair of contigs, weight the rate of consistent and conflicting paired-ends, and then construct scaffolds;

(3) fill gaps: use the paired-end information from the short insert size library and the PCR-free library to retrieve read-pairs that had one end mapped to a unique contig and the other end located in the gap region. We then carried out a local assembly of the collected reads to fill the gaps using Gapcloser.

To evaluate the assembly, we employed CEGMA and EST evaluations. For CEGMA (version 2.4) [19] evaluation, we used 248 ultra-conserved core eukaryotic genes (CEGs) that were widely distributed and conserved in species to assess the completeness of genome assembly and gene-set. The CEGMA evaluation utilized several software packages, including *tblastn* (blast-2.2.25), genewise (wise2.2.3), hmmer (hmmer-3.0), and geneid (geneid v1.4). Four insect genomes including *C. solmsi* were compared. For the EST evaluation, we used *BLAT*[52] to map Sanger-sequenced ESTs or Trinity [53] assembled tgicl [54] clustered unigenes to the *C. solmsi* genome assembly. Then we calculated genome coverage using both the percentage of bases covered by ESTs and the percentage of scaffold numbers with >90% or 50% covered by ESTs. Eight transcriptome datasets were assembled separately by Trinity and then clustered to remove redundancy by tgicl to get unigene sequences before evaluation.

### Gene prediction

Multiple approaches were used to predict gene structures in this genome including *de novo*, homology-based, EST and RNA-seq based predictions. *De novo* prediction was performed based on the repeat-masked genome and with the help of the HMM model using AUGUSTUS [55], GENSCAN, and SNAP. Homologous proteins of the following species were mapped to the genome using *tblastn* with an E-value cutoff 1e-5: *Homo sapiens* (*H. sap*): data downloaded from [56]; *Apis mellifera* (*A. mel*): data downloaded from [57]: *Bombyx mori* (*B. mor*): data downloaded from [58]; *Drosophila melanogaster* (*D. mel*): data downloaded from [59]; and *Nasonia vitripennis* (*N. vit*): data downloaded from [60]. The aligned sequences, as well as their corresponding query proteins, were then filtered and passed to *GeneWise*[61] to search for accurately spliced alignments. ESTs (unpublished data in our lab) were aligned to the genome using *BLAT*[52] to generate spliced alignments. The alignments were then linked according to overlap using *PASA*. Source evidence generated from the above three approaches was integrated by *GLEAN* to produce a consensus gene set.

To improve the integrity and correctness of the genome, transcriptome reads were aligned against the genome using *TopHat* to identify candidate exon regions and the donor and acceptor sites. *Cufflinks*[62] was employed to assemble the alignments into transcripts. Then, based on assembled candidate transcript sequences, ORFs were predicted to get reliable transcripts by using HMM-based training parameters. Finally, we combined the *GLEAN* set with the transcripts from RNA-seq to generate a confident gene set.

### Gene function annotation

Gene functions were assigned according to the best match of the alignments based on *blastp* to the databases SwissProt (release2011 01) [63] and TrEMBL (release2011 01). The motifs and domains of genes were determined by *InterProScan* (iprscan 4.7) [64] against protein databases such as ProDom, PRINTS, Pfam, SMART, PANTHER, and PROSITE. Gene Ontology (GO) [65] IDs for each gene were obtained from the corresponding InterPro entries. All genes were aligned against KEGG (release54) [66] proteins, and the pathway in which the gene might be involved was inferred from matched genes.

### Annotation of repeats and non-coding RNA

Initially, non-interspersed repetitive regions (including simple repeats, satellites, and low complexity repeats) were predicted by RepeatMasker [67] with the ‘-noint’ option. These tandem repeats were also annotated using Tandem Repeats Finder (v.4.04) with parameters of ‘Match = 2, Mismatch = 7, Delta = 7, PM = 80, PI = 10, Minscore = 50, and MaxPeriod = 2000’ [68].

Implementing a homology strategy, we identified known transposable elements (TEs) against the Repbase database (v.20120418) in the genome of *C. solmsi* using RepeatMasker v.open-3.3.0 (ab-blast engine [69], with parameters ‘-nolow, -no_is -norna, -parallel 1 -s’) [67] and RepeatProteinMask (with parameters ‘-noLowSimple, -pvalue 0.0001’) at the DNA and protein level, respectively [70].

A *de novo* repeat library was also generated using RepeatModeler (v1.0.5) [71] and PILER-DF [72], and a RepeatMasker analysis against the final non-redundant library was performed again to find homologs in the genome and to classify the found repeats.

We searched the whole genome sequence to detect four types of non-coding RNAs. Employment of *tRNAscan-SE*[73] identified reliable tRNA positions. We searched for small nuclear RNAs and microRNAs using a two-step method: sequences were aligned with *blast* and then searched with *INFERNAL* against the Rfam database (release 9.1) [74]. The rRNAs were found by aligning with *blastn* against a ref rRNA sequence from the closest related species.

### Orthologous gene clusters and the phylogeny of arthropods

We identified gene families using TreeFam [75] and the following steps: first, *blastp* was used to compare all the protein sequences of 10 species including *C. solmsi*. The E-value threshold was set to 1e-7; second, HSP segments of each protein pair were concatenated by *solar*[50], H-scores were computed based on Bit-scores, and these were taken to evaluate the similarity between genes; finally, gene families were identified by clustering of homologous gene sequences using *hcluster_sg.* Genes specific to *C. solmsi* were those that did not cluster with other arthropods chosen for gene family construction, and those that did not have homologs in the predicted gene repertoire of the compared genomes (Figure 3). However, these genes could have GO annotation if they had the functional motifs. The motifs and domains of these genes were determined by *InterProScan* (iprscan 4.7) [64] against protein databases such as ProDom, PRINTS, Pfam, SMART, PANTHER, and PROSITE. GO IDs of each gene were obtained from the corresponding InterPro entries, from which we also obtained gene functional enrichment.

Single-copy orthologous genes were used to reconstruct the phylogeny. CDS sequences from each gene were aligned using MUSCLE and protein sequences were concatenated to form one super gene for each species. Codon position 2 of aligned CDS sequences was extracted for subsequent analysis. PhyML [76] was used to construct the phylogeny using the GTR substitution model and gamma distribution rates model across sites. Branch reliability was assessed via aLRT values.

Divergence times were estimated using PAML *mcmctree*[77] while implementing the approximate likelihood calculation method. Gamma prior and alpha parameters were computed based on the substitution rate per time unit estimated by PAML *baseml*. We ran *mcmctree* to sample 10,000 times, with sampling frequency set to 5,000, and burnin parameter set to 5,000,000 using correlated molecular clock and REV substitution model. Finetune parameters were set to make acceptance proportions fall in range (0.2, 0.4). Other parameters were the defaults. Convergence of results was checked by *Tracer*[78] and two independent runs were performed to confirm convergence.

### Gene family expansion and contraction

We identified gene families using CAFE [79], which employed a random birth and death model to study gene gains and losses in gene families across a user-specified phylogeny. The global parameter λ, which described both the gene birth (λ) and death (μ = −λ) rate across all branches in the tree for all gene families, was estimated using maximum likelihood. A conditional *P* value was calculated for each gene family, and families with conditional *P* values less than threshold (0.0001) were considered as having an accelerated rate of gain or loss. We identified branches responsible for low overall *P* values of significant families.

### Evolutionary rates of genes

We calculated ka/ks ratios for all single copy orthologs of *C. solmsi*, *Nasonia vitripennis*, *Apis mellifera*, *Camponotus floridanus*, and *Tribolium castaneum*. Alignment quality was essential for estimating positive selection. Thus, orthologous genes were first aligned by PRANK [80], a good alignment tool for studies of molecular evolution. We used Gblocks [81] to remove ambiguously aligned blocks within PRANK alignments. We employed ‘codeml’ in the PAML package [23] with the free-ratio model to estimate Ka, Ks, and Ka/Ks ratios on different branches. The difference in mean Ka/Ks ratios for single-copy orthologous genes between *C. solmsi* and each of the other species were compared with paired Wilcoxon rank sum tests.

Genes that showed values of Ka/Ks higher than 1 along the branch leading to *C. solmsi* were reanalyzed using the codon based branch-site tests implemented in PAML [23,82]. The branch-site model, which allowed ω to vary both among sites in the protein and across branches, was used to detect episodic positive selection. Test 1 (M1a *vs*. branch site model) and test 2 (branch site null model *vs*. branch site model), which differentiated positive selection from the relaxation of selective constrains, were used. The pairwise comparisons M1a *vs*. branch-site model, and branch-site model (model = 2, NSsites = 2) *vs*. branch-site null model (fixed ω = 1 and ω = 1) were used to perform likelihood ratio tests (LRTs). Their significance was evaluated using a χ2 distribution. When the LRT was significant, a Bayes Empirical Bayes (BEB) analysis was conducted to identify putatively positively selected sites, which may also be relaxed selected sites though.

### Manual annotation and evolutionary analyses of interested genes

For genes requiring greater annotation, protein homologs of *N. vitripennis*, *A. mellifera*, and sometimes *D. melanogaster* were collected from NCBI, Hymenoptera Genome Database [83], and FlyBase [84]. Both *tblastn* and *blastp* searches were performed for candidate genes in the assembled genome of *C. solmsi*. The annotated protein set used an E-threshold 0.005. The threshold was raised when protein sequences were short and few blast hits were found. A blast of candidate genes to the NCBI non-redundant (nr) protein database confirmed their orthology. IGV browser was used to view gene annotations, EST, and RNA-seq BAM alignments in the genome of *C. solmsi*. Gene models were refined manually according to RNA evidence and *tblastn* results conducted with the assistance of custom perl scripts. Pseudogenes and irregular features such as missing start codons, stop codons, and other anomalies were noted. For annotation of cuticular proteins with an R&R consensus, the ‘chitin_bind_4’ domain was required. Proteins containing cysteines were removed unless the cysteines lay in signal peptide regions, which were identified by SignalP [85]. For P450s, gene models for *C. solmsi* were searched by *tblastn* and *blastp* against *D. melanogaster*, *A. mellifera*, and *N. vitripennis* CYP sequences representing the CYP2, 3, 4 and mitochondrial P450 clans (E-value cutoff = 10^-4^). All models with predicted proteins that included the canonical heme binding sequence were manually verified for the presence of the other key features of P450 enzymes; the gene model was corrected whenever necessary (incorrect predictions such as fusions with adjacent genes or fragmentation) or possible (when RNA-seq sequences were available). Final gene models were confirmed by blasting back to the reference gene set to confirm reciprocal best hits. The obtained gene models were inspected and, if necessary, edited. Care was taken to ensure that the predicted gene structures matched corresponding transcriptomic data. Pseudogenes and gene fragments (detritus exons) were separated from putative full-length CYP coding sequences. To annotate genes involved in the development of eyes and wings, proteins putatively participating in development of eyes and wings described for *D. melanogaster* were used as query sequences. These queries were used in *blastp* and *tblastn* searches (E-value cutoff = 10^-4^) against the protein predictions and scaffolds of the *C. solmsi* genome. Iterative searches were also conducted with each new protein of *C. solmsi* as a query until no new genes were identified in each major subfamily or lineage.

To further understand the evolutionary history and homologies between gene families of *C. solmsi* and other insects, we performed a phylogenetic analysis using the genes found in *C. solmsi* and some other insect taxa with completed genomes: *A. mellifera*, *N. vitripennis*, and *D. melanogaster*. The amino acid sequences of homologous genes were aligned with ClustalX v2.0 [86]. ProtTest [87] identified evolutionary models that best fit this dataset according to the Akaike Information Criterion. A maximum likelihood tree was then reconstructed with PhyML 3.0 using the best-fit model with a gamma correction using four discrete classes, an estimated alpha parameter and proportion of invariable sites [76]. Node support values were obtained by the rapid bootstrap algorithm as implemented in PhyML 3.0 (100 replicates). Some tree images were created using the iTOL web server [88]. Gray circles on branches were used to indicate bootstrap values >80% from 100 bootstrap replicates.

We tested for selection on Gustatory receptor (Gr) and Olfactory receptor (Or) genes. The calculation of each Ka/Ks value of Gr or Or gene were based on each orthologous group of Gr or Or gene members among *A. mellifera*, *N. vitripennis*, and *C. solmsi*. The difference between mean Ka/Ks of Or plus Gr genes and all single-copy genes was compared using paired Wilcoxon rank sum tests. This determined if Or and Gr genes underwent different selective pressures than single-copy genes.

### Validation of extremely contracted CCE, OBP, and Gr gene families

Since the fig wasp had many fewer genes than other insect species, we performed additional analyses to confirm that the absence of genes is not due to a poor or incomplete assembly or inadequate annotation. Validation of assembly quality was fully analyzed and described above (see genome assembly). We also tried to confirm the absence of genes by focusing on the annotations of the gene families CCE, OBP, and Gr, in which the fig wasp had the fewest gene members among the studied insects, by delving into the raw genomic reads.

We chose the library with the inserted fragment size of 500 bp (altogether 68,549,132 pair end reads after correction), which was 25× coverage of the genome, and aligned it to the assembled genome of *C. solmsi* by SOAP [89] (default except: -v, 5; -g, 3). Altogether, 91.2% of reads mapped to the genome. We then compared the unmapped reads (= 6,042,453) to the protein sequences of all CCE, OBP, and Gr members of the fig wasp, jewel wasp, and honeybee by *blastx*[69] (references given in Table 2 and Additional file 1: Table S10) with e-value of 1e-5. Only one read mapped to gene AmGr9 (a Gr gene member in the honeybee), which indicated that the unmapped reads scarcely had any similar sequences in the three gene families. Thus, the absence of genes in these gene families was not due to incomplete annotation.

### Detection of horizontal gene transfer

Two independent approaches were used to identify possible HGT events. The first used gene models. We used *blastp* (*E*-value cutoff 10^-10^ and a continuous overlap threshold of 33%) to compare the predicted protein sequences of *C. solmsi* against sequences in the RefSeq and nr databases to exclude unique genes and those with high similarity to other insects. Next, we constructed phylogenies for the retained proteins with highest similarity to non-insects. Multiple alignments were performed by using ClustalW2 [86], followed by manual refinement. Phylogenetic analyses were conducted using maximum likelihood (ML) and Bayesian inference (BI). A distance-based phenogram was also constructed using neighbor joining (NJ). ML trees were estimated by PhyML [76] using best-fit substitution model estimated by Prottest 3.0 [87]. In all cases, we used a discrete gamma-distribution model with four rate-categories plus invariant positions. The gamma parameter and proportion of invariant sites were estimated from the data. Bootstrap branch-support values involved 1,000 pseudoreplicates. BI used MrBayes 3.1.2 [93]. For each HGT, we ran two independent analyses using four MCMC chains (one cold and three hot) for one million generations and stopped them when the average deviation of split frequencies fell well below 0.01. We sampled trees every 100 generations and discarded the initial 25% of the total trees as burn-in. Compatible groups were shown in the majority rule consensus tree. NJ trees were constructed by using Neighbor in Mega5 [94]. Bootstrap values were obtained by generating 1,000 pseudoreplicates. HGTs were detected by clustering non-related species on a well-supported node.

Using the scaffold sets of *C. solmsi*, we identified regions involved in recent HGT events between bacteria, fungi, plants, and other non-insects to *C. solmsi*. The pipeline involved *blastn* of *C. solmsi* against other non-insects. Usually, this approach has detected two categories of genes: candidate HGT events; and highly conserved genes shared by non-insects and *C. solmsi* (not HGTs). Thus, the pipeline formed two categories based on the following certain criteria:

HGT_scaf: non-insects score > animal score AND scaffold length > 5 K AND range_per < 90%;

highly_cons: best non-insects score < insect score.

The scaffolds with both non-insect and insect sequences served as evidence of non-contamination.

### Data availability

Data for this Whole Genome Shotgun project were deposited at DDBJ/EMBL/GenBank under the accession no. ATAC00000000. The version described in this paper is version ATAC01000000. The transcriptome data reported in this paper were deposited in the National Center for Biotechnology Information Short Read Archive [95] under the accession no. SRP029703.

## Abbreviations

AMP: Antimicrobial peptides; CCE: Carboxylesterases; CPR: Cuticular protein; CSP: Chemosensory protein; CYP: Cytochrome P450; Gr: Gustatory receptor; GST: Glutathione-S-transferases; HGT: Horizontal gene transfer; IMD: Immunodeficiency; Ir: Ionotropic receptors; JAK/STAT: Janus kinase/signal transducers and activators of transcription; JNK: c-jun N-terminal kinase; OBP: Odorant binding protein; Or: Olfactory receptor; P450: Cytochrome P450.

## Competing interests

The authors declare that they have no competing interests.

## Authors’ contributions

DWH and JHX initiated the project and designed the study. JHX, ZY, LYJ, XHY, LHN, ZW, PZ, BFS, SMH, ZL, TLX, WX, HFG, and BW performed the research and generated the data. JHW, DW, LMN, GCM, TT, SNB, NXW, CYY, NW, YGF, SVY, XYY, QZ, CXL, CYX, LJH, LLY, MC, YZ, SWW, SZ, YHL, YYY, XJQ, YC, LLB, SZ, JYW, YY, HX, GHW, HY, WSW, and JW analyzed the data. WZL participated in preparation of Figure 1. JHX, DWH, JMC, RWM, JHW, XHY, and SVY wrote the paper. All authors read and approved the final manuscript.

## Supplementary Material

Additional file 1 **Supplementary text, Figures S1 to S19, Tables S1 to S22, and supplementary references.** The supplementary text details the manual annotations of gene families involved in heat shock proteins, development of compound eyes and wings, circadian rhythm, yellow and royal jelly-like proteins, the hox complex, and sex determination in the *C. solmsi* genome. We also provide evidence on DNA methylation, and nutritional and microbial analysis in the genome. Click here for file
