# Supplementary material for: Obligate mutualism within a host drives the extreme specialization of a fig wasp genome
Source: Genome Biol. 2013 Dec 20;14(12):R141. doi: 10.1186/gb-2013-14-12-r141 (PMC4053974; doi:10.1186/gb-2013-14-12-r141)
Supplement: Additional file 1 — Supplementary text, Figures S1 to S19, Tables S1 to S22, and supplementary references. The supplementary text details the manual annotations of gene families involved in heat shock proteins, development of compound eyes and wings, circadian rhythm, yellow and royal jelly-like proteins, the hox complex, and sex determination in the C. solmsi genome. We also provide evidence on DNA methylation, and nutritional and microbial analysis in the genome. [file gb-2013-14-12-r141-S1.pdf]

Additional file for

Obligate mutualism within a host drives the extreme specialization of an insect genome

Jin-Hua Xiao*, Zhen Yue*, Ling-Yi Jia*, Xin-Hua Yang*, Li-Hua Niu*, Zhuo Wang*, Peng Zhang*, Bao-Fa Sun, Shun-Min He, Zi Li, Tuan-Lin Xiong, Wen Xin, Hai-Feng Gu, Bo Wang, John H Werren, Robert W Murphy, David Wheeler, Li-Ming Niu, Guang-Chang Ma, Ting Tang, Sheng-Nan Bian, Ning-Xin Wang, Chun-Yan Yang, Nan Wang, Yue-Guan Fu, Wen-Zhu Li, Soojin V. Yi, Xing-Yu Yang, Qing Zhou, Chang-Xin Lu, Chun-Yan Xu, Li-Juan He, Li-Li Yu, Ming Chen, Yuan Zheng, Shao-Wei Wang, Shuang Zhao, Yan-Hong Li, Yang-Yang Yu, Xiao-Ju Qian, Yue Cai, Lian-Le Bian, Shu Zhang, Jun-Yi Wang, Ye Yin, Hui Xiao, Guan-Hong Wang, Hui Yu, Wen-Shan Wu, James M. Cook#, Jun Wang#, Da-Wei Huang#

* These authors contributed equally to this work.
# To whom correspondence should be addressed. Email: huangdw@ioz.ac.cn (D.W.H.); wangj@genomics.cn (J.W.); James.Cook@uws.edu.au (J.M.C.).

This file includes
Supplementary text
Figure S1 to S19
Table S1 to S22
Supplementary references

Supplementary text

This supplementary text includes the detailed analyses on the manual annotation of gene families including heat shock proteins, development of compound eyes and wings, circadian rhythm, yellow and royal jelly-like proteins, hox complex, and sex determination in the *Ceratosolen solmsi* genome. We also provide evidence on DNA methylation, and nutritional and microbial analysis in the genome.

Heat shock proteins

Most living organisms produce heat shock proteins (HSPs) in response to a range of environmental stresses besides heat. These molecular chaperons help in protein stabilization, or as proteases to degrade damaged proteins; they may also serve as signaling proteins in immune responses [1, 2]. We annotated 47 genes of varying molecular weight encoding HSPs (Table S15). Trees constructed for genes encoding HSP70 and HSP90 indicate that fig pollinators and closely related jewel wasps have similar genes (Figure S9).

Gene members involved in development of compound eyes and wings

Female and male *C. solmsi* are extremely divergent morphologically in their compound eyes, wings, antennae, body color, and size as functional adaptations to different lifestyles in fig syconia (Figure 2). Females must emerge from syconia and fly to another fig tree to oviposit and pollinate; they need fully developed compound eyes (with ocelli), wings, and antennae. In contrast, males have no compound eyes (ocelli absent), vestige wings and shorter antennae because these characters are advantageous for living inside the syconia. We focus on the genes that might be involved in the development of insect compound eyes and wings.

Manual annotation of all genes suggested to be involved in the development of eyes identified 61 genes in the fig wasp genome for which the *D. melanogaster* has 65 orthologs (Table S16). Several genes missing in the fig wasp genome involve those that seem to be restricted to *Drosophila*, such as *twin of eyegone*, *phyllopod*, *drosocrystallin*, and *PvuIIPstI homology 13* [3]. The fig wasp lacks *seven in absentia*, *melted*, *iroC*, and the gene encoding sine oculis binding protein; these are also lost in the genome of *N. vitripennis*. Regardless, the fig wasp genome also has notable duplications of genes involved in eye development. Whereas *Drosophila* has one *homothorax*, all other insect genomes have two counterparts. The fig wasp has two *big brother* genes, which function during cell fate specification in the eye [4]. It also has two *hairy* genes and even five duplicates of *chaoptin*, which seems to be similar with *Nasonia* (Figure S10). Chaoptin is essential for the development and maintenance of photoreceptor cells, and in *Drosophila* it helps organize the inner architecture of the compound eyes [5, 6]. The duplication of *chaoptin* may be linked with the diversification of rhabdom. Fruit flies have an open rhabdom system, in which the seven rhabdomeres of each ommatidium are structurally and functionally separate units. In the Hymenoptera, the system is closed; rhabdomeres within each ommatidium are fused and share the same visual axis (Figure S11). This area deserves further study. Altogether, 55 genes that may be involved in the development of insects' wings were also annotated (Table S17).

Circadian rhythm genes

Most organisms have circadian rhythm in their gene expression and behavior as adaptations to environmental changes. The circadian systems have three major components: an endogenous circadian clock for time keeping; input pathway for synchronizing the clock to environment; and an outputting pathway for modulating physiology and behavior [7]. The circadian phase of insects synchronizes to

24-hour cycles of light and temperature via a key photoreceptor protein [8]. Fig wasps spend most of their lifetime within the syconium throughout their maturation. Though the light environment within the syconium is obviously different from that outside, fig wasps inside still have circadian rhythms in their gene expressions [9] and behaviors. For example, mature wasps emerge out from the syconium only in the daytime and most frequently in the early morning hours. We are very interested in the genes involved in the synchronization of the clock to environment.

Components of the clock are largely conserved across a broad range of species yet insects have diverse clock structure and function [10]. Fig wasps share eight putative principle clock genes with honeybees and jewel wasps: *period, timeout, cycle, clock, cryptochrome, vrille, par domain protein 1,* and *clockwork orange*. They also share the output circadian gene *pdf* (Table S18). The gene sequences and structure of most genes are conserved in the fig wasp and honeybee. However, *timeout* in the fig wasp differs from that of other insects. It has an intron of around 90 kb length between the fifth and sixth exon, which harbors another gene (CSO_004810). We cannot obtain PCR products in the cDNA template with specific primers located in the fifth and sixth exons, indicating that the two exons are located in different transcripts. Furthermore, expression profiles of RNA-seq data reveal a polyA signal in the 3' end of the fifth exon. These observations suggest that *timeout* is separated into two genes in the fig wasps.

Yellow and Royal Jelly-like Proteins

Yellow-like proteins occur mostly in insects, as well as in certain bacteria and fungi. They have been implicated in pigmentation, development and reproductive maturation in insects [11, 12]. Multiple genes encode Yellow-like proteins and they range from eight in *Drosophila pseudoobscura* to 20 and 26 in *A. mellifera* and *N. vitripennis*. Phylogenetic relationships indicate putative functions. The genomes of *A. mellifera* and *N. vitripennis* harbor a subfamily of genes encoding Major Royal Jelly Proteins (MRJPs) or Royal Jelly-like Proteins (RJPLs). MRJPs appear to function as components of larval food for bees while the functions of RJPLs are unknown [13]. Both subfamilies originate from *yellow-e3* as independent duplications. They form gene clusters in almost syntenic genomic regions, with nine genes in *A. mellifera* and 10 in *N. vitripennis*.

The genome of *C. solmsi* contains only 12 *yellow*-like genes, less than the other two hymenopteran genomes. However, it harbors members of all subfamilies of *yellow*-like genes in *N. vitripennis* (Table S19). This indicates that the genome of *C. solmsi* has the basic genes required to fulfill the functions of this gene family. The genome of *C. solmsi* has only two tandem *rjpl* genes similar to those of *N. vitripennis*. This indicates that *C. solmsi* has lost *rjpl* genes, as have ants [14]. The genome of *C. solmsi* has a syntenic genomic region of five gene members that are similar to the seven of *N. vitripennis*. Transcription patterns at four different life stages reveal that most of the other *yellow*-like genes have relatively higher expression in pupae than in adults, except for *e3, g* and *g2*, which maintain only a basic level of transcription through all stages (Figure S12). The expression of *Cs-yellow* (CSO_007840) is 70 times higher in female than male *C. solmsi* at the late pupa stage, which suggests it plays an important role in the formation of pigmentation.

Hox complex genes

Homeobox genes encode homeodomain-containing proteins, a major class of transcriptional factors that regulate a variety of developmental processes including pattern formation, segmentation, cell cycle regulation and differentiation, among others [15]. The complex is a special group of Hox genes; their conservative and successional clustering along a chromosome can determine the identity of a segment along the anterior-posterior axis of the embryo. Although the co-linear order of the Hox complex genes on a

chromosome tends to be conserved, many distinct divergences exist, including complex structure, size, and expression patterns. The Hox cluster consists of two parts in *Drosophila* and some other insects. In contrast, the honeybee has 10 conserved genes directly linked together on the same chromosome [16, 17].

The fig wasp genome has 90 Hox genes (Table S20). The Hox complex (1.6 Mb in size) has canonical characteristics of the insect Hox gene cluster, like that of honeybee, in that it has all 10 expected Hox genes on the same scaffold (Scaffold 56) and transcribed from the same strand (Figure S13). The size of the Hox complex of the honeybee was about 1.37 Mb, larger than the 0.66 Mb of *Drosophila*, 0.70 Mb of *Tribolium*, and 1.18 Mb of *Anopheles*[17]. The honeybee Hox complex has an insertion of one protein coding gene (non-homeobox) and three transposable elements, while no insertion of other protein-coding genes occurs in the Hox cluster of the fig wasp. The size variation of the complex seems to be due to the intergenic region (average 137,432 bp/region of fig wasp compared to 115,890 bp/region of the honeybee resulting a ratio of 1.186, which is similar with the ratio of complex size: 1.6 Mb versus 1.37 Mb makes a ratio of 1.168). No transposable elements occur in the Hox complex. Among three miRNAs, *mir10* occurs between *Dfd* and *Scr*, and *mir-iab-4* and *mir1027 4* occur between *Abd-A* and *Abd-B*. The former two are conserved in insects in their sequences and locations [18], while the other one constitutes a new insertion in the fig wasp Hox complex (Figure S13).

Genes involved in sex determination

As in other Hymenoptera, fig wasp males are haploid and females are diploid. Many hypotheses attempt to explain the mechanism of sex determination in male haploid hymenopterans, such as CSD (complementary sex determination, encoded by *csd*) for honeybees, ants and other species [19, 20], as well as maternal imprinting of *transformer* (*tra*) for *N. vitripennis* [21]. In *A. mellifera*, the homolog of the *tra* gene of *Drosophila* is named *feminizer* (*fem*).

In the major sex determination models, *csd* and/or *tra* occupy important positions. Gene *csd* is believed to have arisen from the duplication of *fem*, and both genes occur in the genome of most Aculeata [20]. However, the absence of *csd* and high inbreeding levels rule it out as the primary regulator of sex determination in *N. vitripennis*. Our research seems to confirm this. The annotation detects 16 genes involved in sex determination of *C. solmsi* (Table S21) and reveals the absence of *csd* orthologs, in common with *N. vitripennis*. Like *N. vitripennis*, fig pollinators also have a high probability of inbreeding. Thus, sex determination in *C. solmsi* likely follows the same pathway of *N. vitripennis*, which does not involve CSD.

DNA methylation in the genome

1) Methods for CpG o/e analyses

We compared CpG o/e [CpG observed/ expected (o/e)] of different genomic regions. For the genomic background, we randomly cut the scaffolds into 1000bps fragments. CDs (gene coding) contained combined exons for each gene. For each genomic region, we calculated CpG o/e as well as GpC o/e. We estimated CpG o/e using the following formula:

$$CpGo/e = \frac{\boldsymbol{P}_{CpG}}{\boldsymbol{P}_C * \boldsymbol{P}_G},$$

where $\boldsymbol{P}_{CpG}$, $\boldsymbol{P}_C$ *and* $\boldsymbol{P}_G$ were the frequencies of CpG dinucleotides, C nucleotides, and G nucleotides, respectively, estimated from each genomic fragment.

2) Annotation and clustering of DNA methylation machinery genes

We first performed a *blastp* search against the human, honeybee, and DNA methyl transferases (DNMTs) of *Nasonia* using all fig wasp protein sequences as queries with an e-value threshold of 1e-20. We also performed clustering analyses of DNMTs using neighbor-joining.

3) Evidence of DNA methylation

DNA methylation plays an important role in gene regulation in animals[22]. DNMTs add a methyl group to cytosine bases, predominantly in 5'-CpG-3' dinucleotides (CpG). All DNMTs share a conserved catalytic domain, suggesting a common, ancient origin. Different mammalian DNMTs undertake distinct functions. For example, human genomes contain one DNMT1, one DNMT2, and two DNMT3s (DNMT3a/b). DNMT1 maintains the pattern of DNA methylation between DNA replications and is referred to as the maintenance methyltransferase. DNMT3s mediate *de novo* methylation of previously unmethylated cytosines. Although not completely resolved, DNMT2 may act as a tRNA methyltransferase [23]. Several hymenopteran insect genomes, such as that of *N. vitripennis,* contain all three DNMTs [13]. However, the fig wasp genome encodes two putative DNMT1, one putative DNMT2, and one putative DNMT3. Clustering analysis of DNMTs shows the expected relationship among the three DNMTs classes (Figure S14); they appear to be homologs.

Because DNA methylation in animals almost entirely targets CpG dinucleotides, and methylated cytosines are highly vulnerable to spontaneous deamination, which causes a C to T transition, methylated genomic regions will gradually lose their CpG dinucleotides [24, 25]. Thus, we can detect DNA methylation through its evolutionary signature in the form of CpG depletion by analyzing normalized CpG dinucleotide content. We compare CpG o/e of different genomic regions in the fig wasp genome (Figure S15). The mean CpG o/e in the whole genome is 1.33, indicating an over-representation of CpG dinucleotides at the genomic level. Similar patterns have been observed in other hymenopterans such as the honeybee [26]. The distributions of CpG o/e in the genome, introns and UTRs are all similar to each other. However, gene coding (CDs) regions exhibit conspicuously lower CpG o/e compared to other genomic regions, with a mean value of only 1.01, about 24% less than the genomic background ($P< 10^{-16}$). This result is consistent with previous studies that, in contrast to the pattern of genome-wide DNA methylation in vertebrates, DNA methylation in invertebrates is relatively sparse and largely confined to transcription units or gene bodies [22]. In particular, in hymenopterans, DNA methylation occurs more frequently in exons than introns[27, 28].

We also analyze the distribution of other dinucleotides in fig wasp CDs. Among all 16 dinucleotides, the CpG o/e distribution is unique, because it is the only one that exhibits a characteristic 'fat tail' on the lower CpG o/e range. This pattern differs conspicuously when compared to GpC o/e (Figure S16) and indicates that the genes in the low CpG o/e regions may have DNA methylation. The difference between CpG o/e and GpC o/e can identify potentially methylated genes and application of a 60% lower value for the former relative to the latter identifies 1594 potentially methylated genes. These genes are distinctly enriched in the processes of biosynthesis and metabolism. Interestingly, these genes are significantly devoid of fig wasp-specific genes (Table S22). This observation is consistent with studies indicating that methylated genes tend to be highly conserved at the sequence level and most orthologs that occur over long evolutionary timescales exhibit DNA methylation [28, 29].

Nutritional and microbial analyses detect possible microbial symbiosis

After struggling into a fig cavity for pollination and oviposition, female pollinators die. This long-evolved maternal sacrifice may supply their offspring with peculiar nutritional foods or a much safer living environment within the fig syconia. Thus, we predict that the fig wasp may have evolved a reduction in their nutrient acquisition system during the long evolutionary history of living inside the fig cavity.

We performed a metabolic reconstruction analysis using the Kyoto Encyclopedia of Genes and Genomes (KEGG) on the genome of the fig wasp and compared the results with *D. melanogaster*, *N. vitripennis*, *Atta cephalotes*, and *A. pisum*. Like other insects, fig wasps cannot *de novo* synthesize histidine, isoleucine, leucine, lysine, methionine, phenylalanine, threonine, tryptophan, and valine, which may be easily acquired from their foods. However, *C. solmsi*, *A. cephalotes*, and *A. pisum* have also lost the biosynthesis pathway for arginine, because they have independently lost two key genes that catalyze the synthesis of arginine from citrulline and aspartate: argininosuccinate synthase (EC 6.3.4.5) and argininosuccinate lyase (EC 4.3.2.1) (Figure S17).

The loss of these two genes indicates a dependence on externally-acquired arginine. The pea aphid *A. pisum* depends entirely on its endosymbiont *Buchnera aphidicola* for arginine [30] and the leaf-cutter ant *A. cephalotes* gets arginine from the mutualistic fungus it cultivates [14]. Thus, we were curious to know how fig wasps acquire arginine. Transcriptomic data from unvisited ovaries of *Ficus hispida* obtained using RNA-seq provide insights into the metabolic pathway of arginine in fig trees. The preponderant bacteria (up to 47%) of fig wasps are in the genus *Enterobacter* (Proteobacteria: Enterobacteriaceae) (Figure S18). The predominant fungi in fig wasps are in *Candida* (Dikarya: Saccharomycetaceae) and this is consistent with its occurrence in fig fruit [31] (Figure S19). In early-stage fig ovaries unvisited by fig wasps the preponderant bacteria are *Delftia* and *Acinetobacter* (unpublished data).

We mapped the presence of genes involved in the metabolism of arginine in these bacteria, fungi, and plant onto the metabolism pathway (Figure S17) and compared them with those of the insects. The fig tree has a complete biosynthesis pathway for arginine, like all other plants. It was not possible to determine if the fig tree had surplus arginine for the fig wasp (http://www.genome.jp/kegg-bin/show_pathway?org_name=zma&mapno=00330&mapscale=1.0&show_description=show).

The pattern of fungi is similar to that of plants. Both *Delftia* and *Acinetobacter* seem to have a good self-balance in the biosynthesis and consumption of arginine. However, the bacteria associated with fig wasps (*Enterobacter*), is much like *Buchnera* in synthesizing arginine; it can provide its hosts with arginine. Furthermore, unlike *Buchnera*, *Enterobacter* can synthesize arginine from citrulline in another pathway, with the presence of arginine deiminase (EC 3.5.3.6), which is rare in nature. PCR experiments confirm that all genes encoding argininosuccinate synthase (EC 6.3.4.5), argininosuccinate lyase (EC 4.3.2.1), and arginine deiminase (EC 3.5.3.6) occur in *Enterobacter* of fig wasps. Thus, the fig wasp is more likely to get arginine from the significant accessory symbiont *Enterobacter* it harbors rather than from the fig tree or fungi.

Supplementary figures

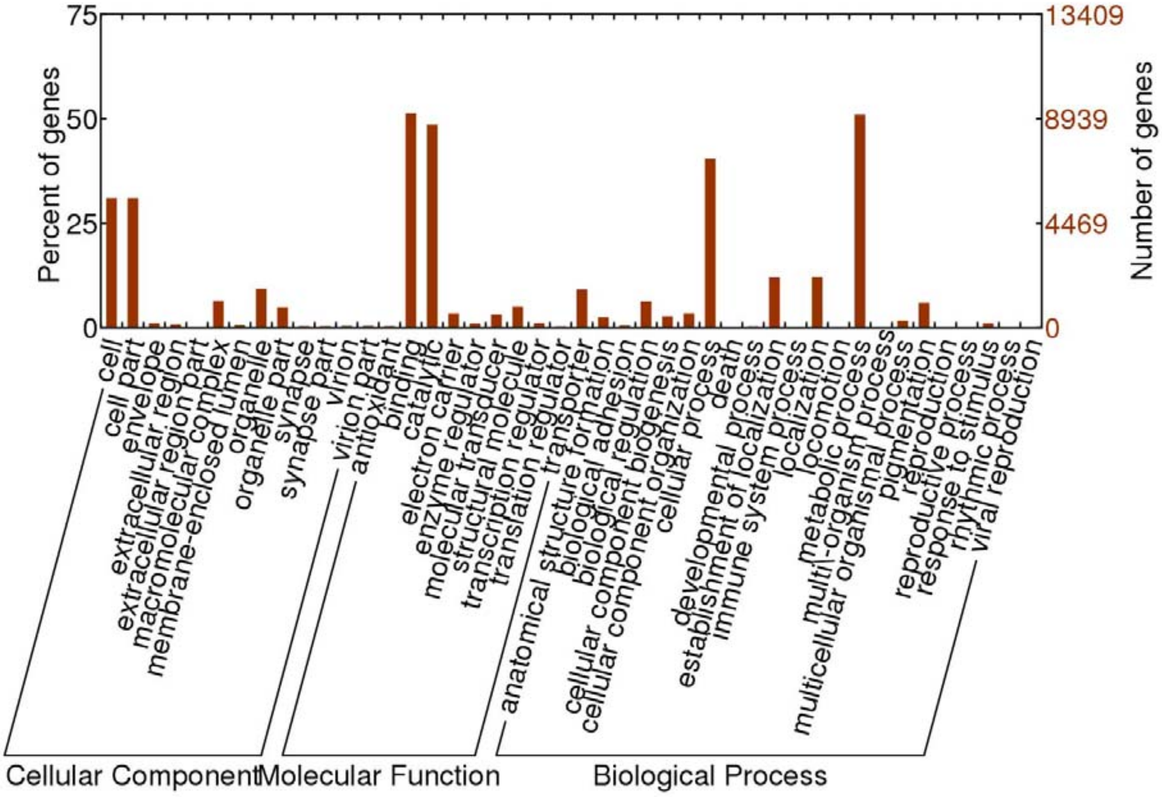

Figure S1. GO enrichment pattern of contracted genes families in fig wasps. Compared with other insects, contracted genes families in fig wasps are enriched in binding and catalytic functions or in cellular and metabolic processes.

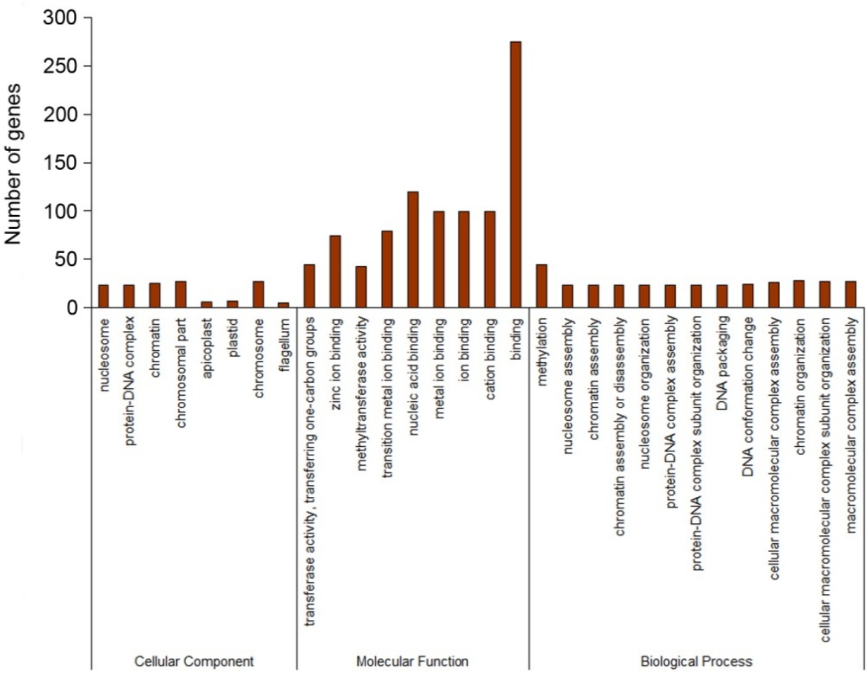

Figure S2. Significant GO enrichment of genes unique to fig wasps.

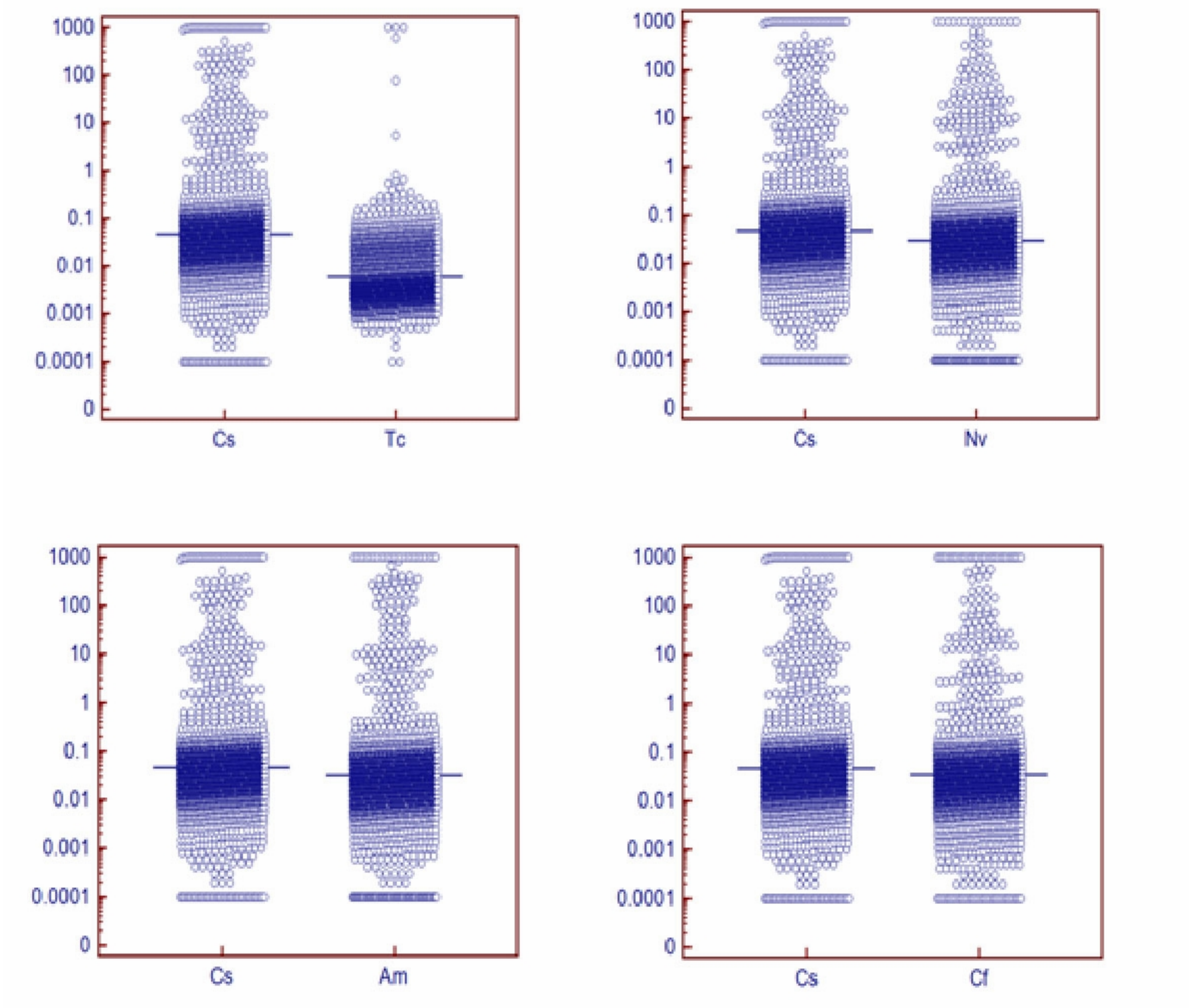

Figure S3. Comparison of the mean omega ratios of all single copy orthologous genes. The average omega of *C. solmsi* genome is higher than other insects (paired Wilcoxon rank sum tests: P< 0.0001). Mean omega value: *Cs,* 0.039; *Tc,* 0.004; *Nv*, 0.026; *Am,* 0.028; *Cf*, 0.029. (*Cs*: *Ceratosolen solmsi*, *Tc*: *Tribolium castaneum*, *Nv*: *Nasonia vitripennis*, *Am*: *Apis mellifera*, *Cf*: *Camponotus floridanus*).

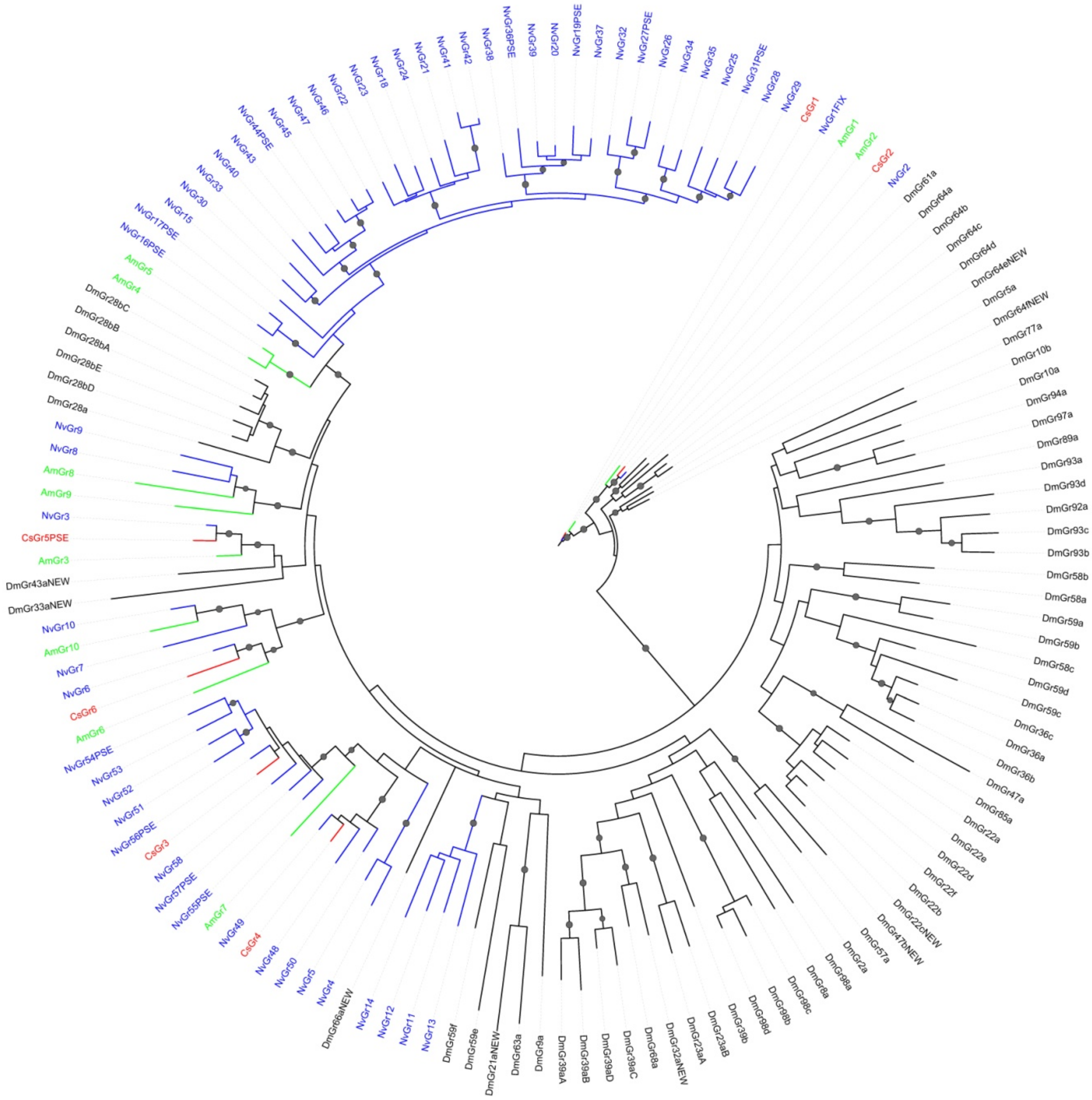

Figure S4. Gene tree constructed from all gustatory receptor genes (Grs) of four insect species. The *C. solmsi* genome has much reduced Gr members. Notes: *Ceratosolen solmsi* (red); *Nasonia vitripennis* (blue); *Apis mellifera* (green); *Drosophila melanogaster* (black).

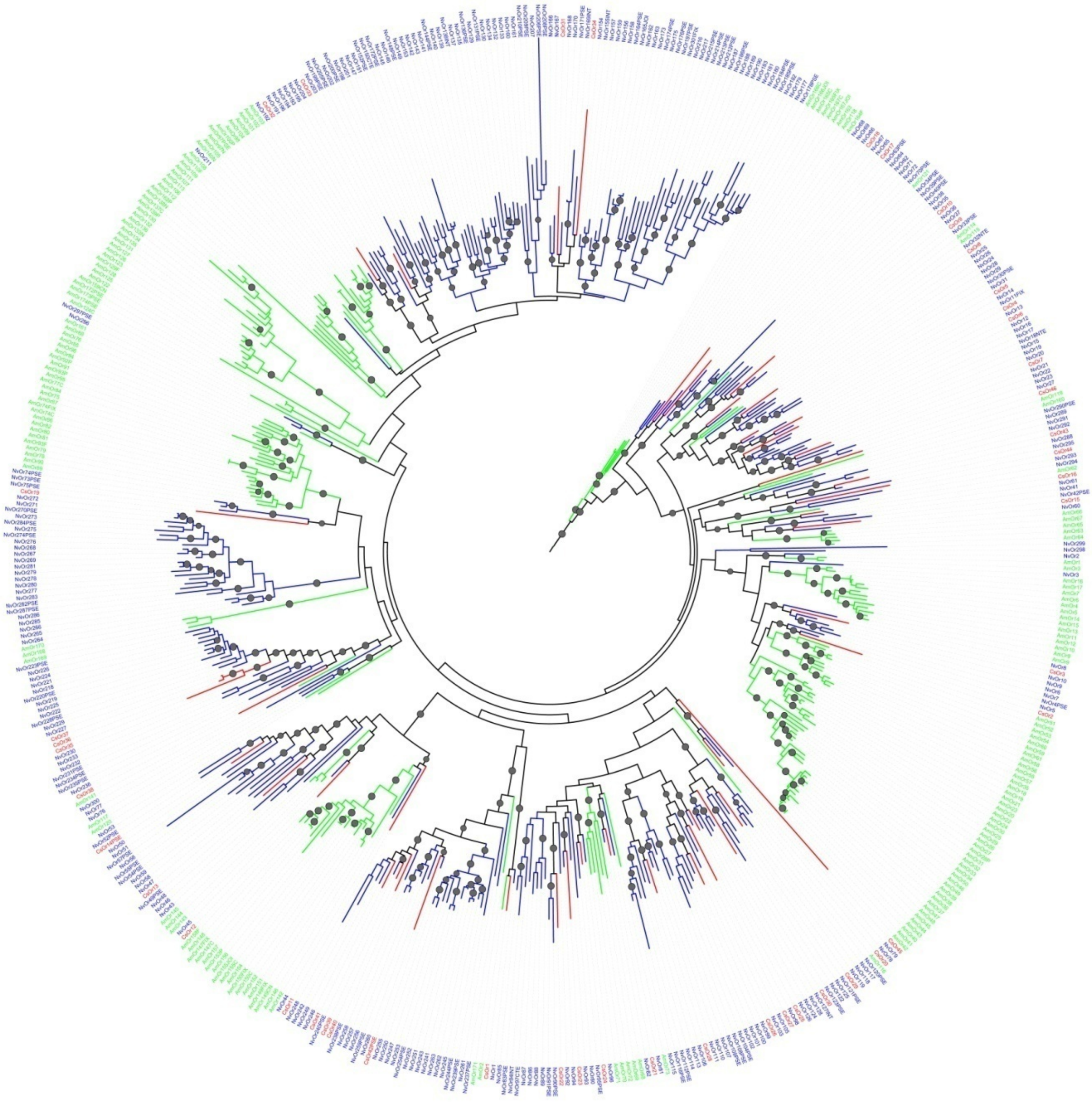

Figure S5. Gene tree constructed with all the olfactory receptor genes (Ors) of three hymenopteran insect species. Notes: *Ceratosolen solmsi* (red); *Nasonia vitripennis* (blue); *Apis mellifera* (green).

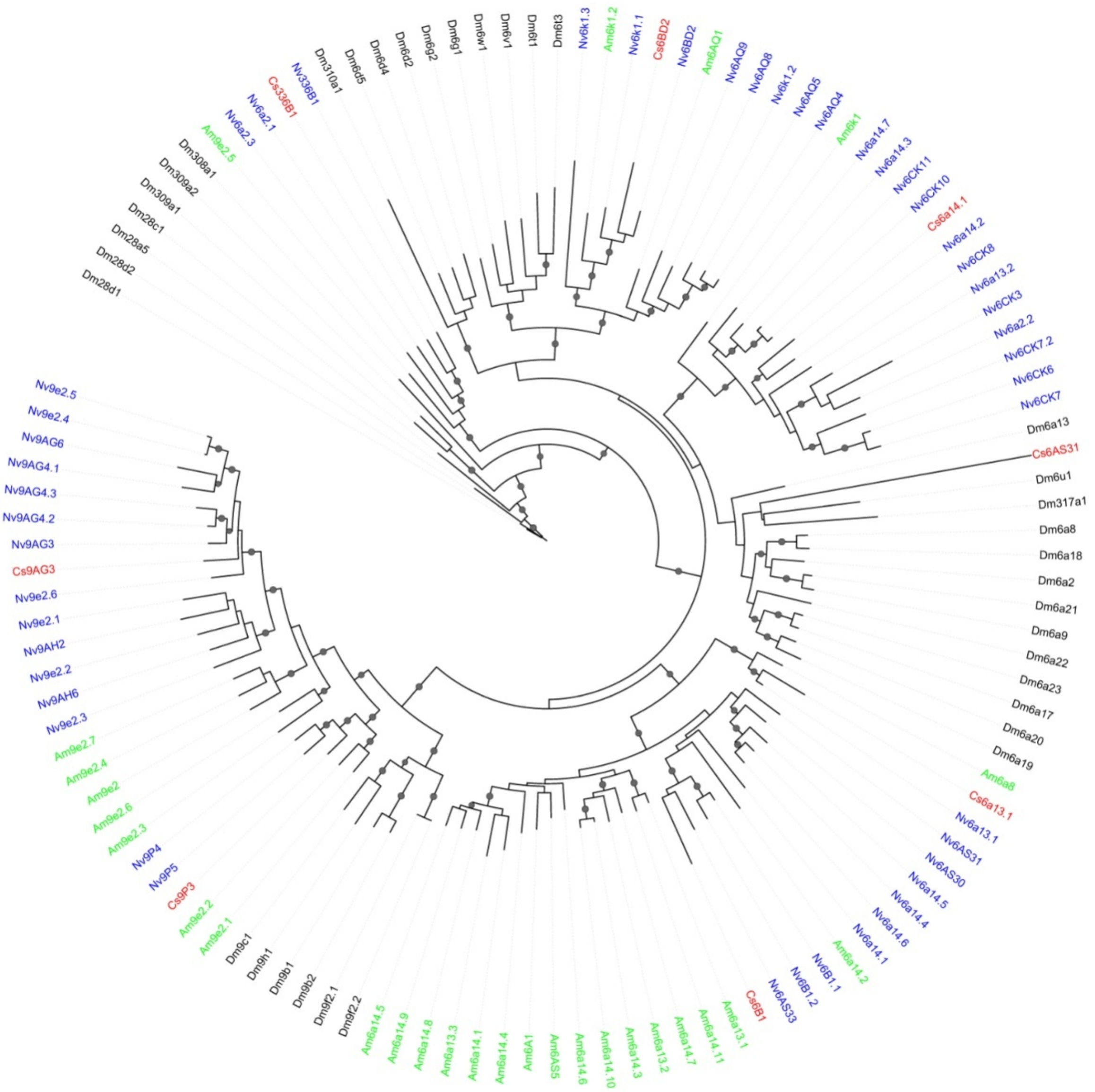

Figure S6. Gene tree constructed with all the CYP3 genes of four insect species. The *C. solmsi* genome has much reduced gene members in CYP3 clan. Notes: *Ceratosolen solmsi* (red); *Nasonia vitripennis* (blue); *Apis mellifera* (green); *Drosophila melanogaster* (black).

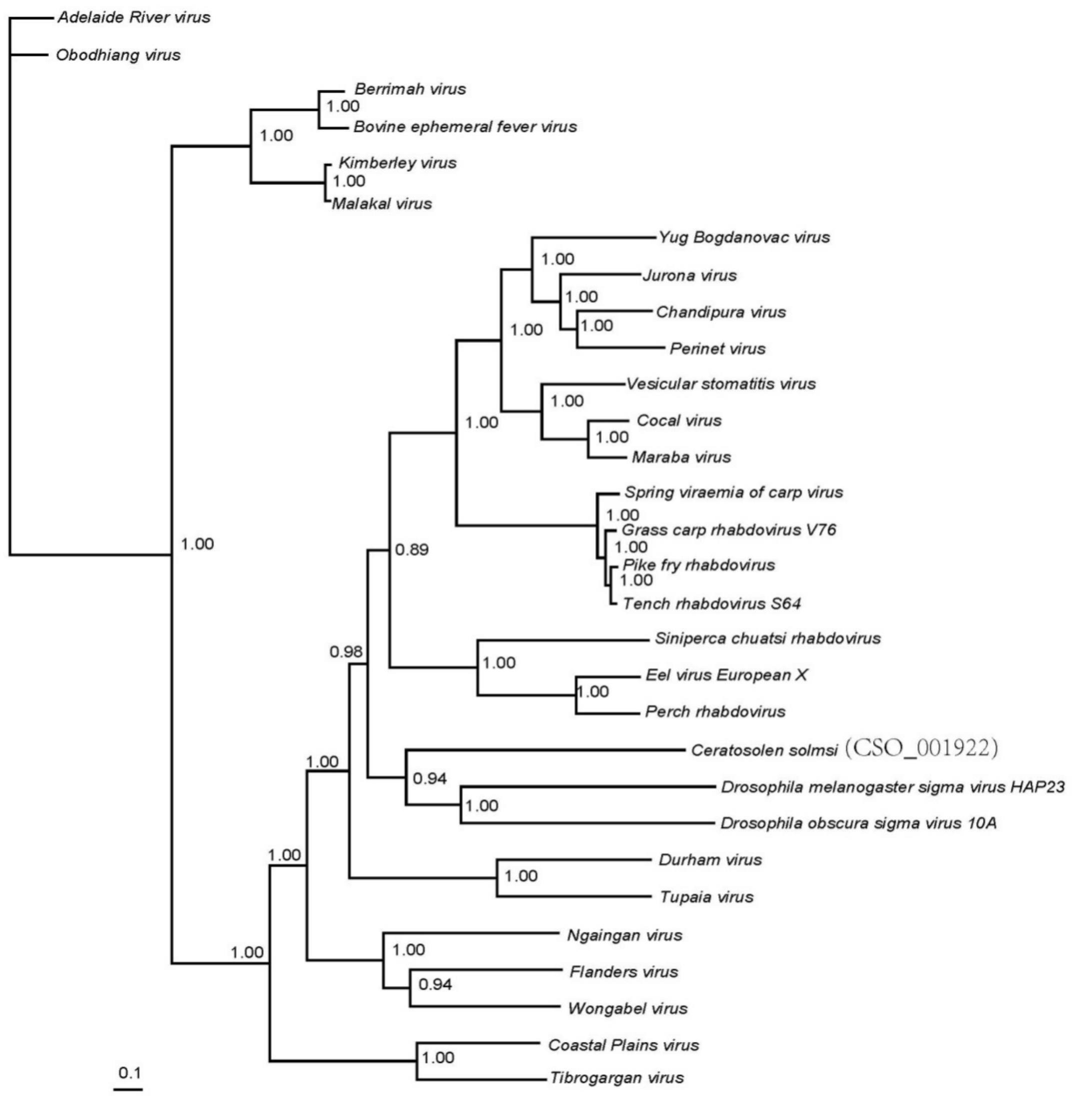

Figure S7. Phylogeny indicating the viral origin of HGT gene in fig wasp genome (indicated as CSO_001922).

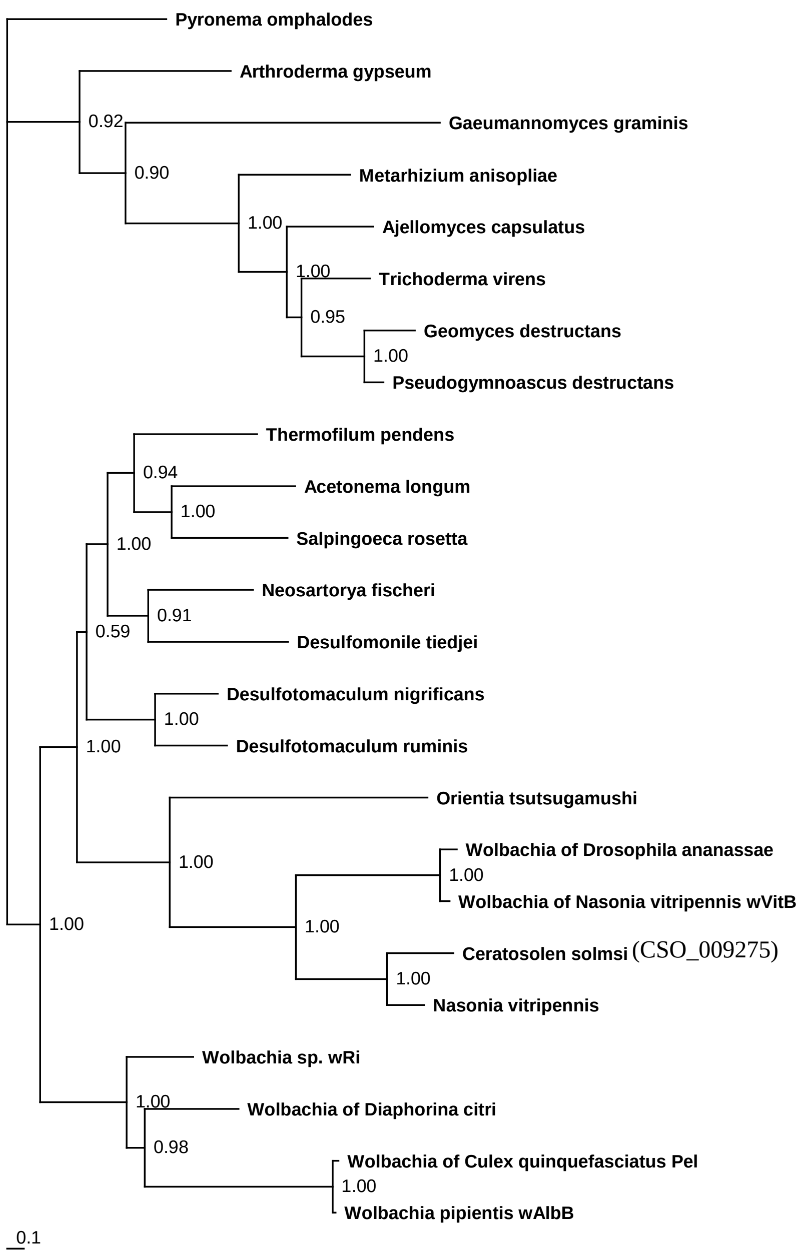

Figure S8. Phylogeny indicating the bacterial origin of HGT gene in the fig wasp genome (indicated as CSO_009275).

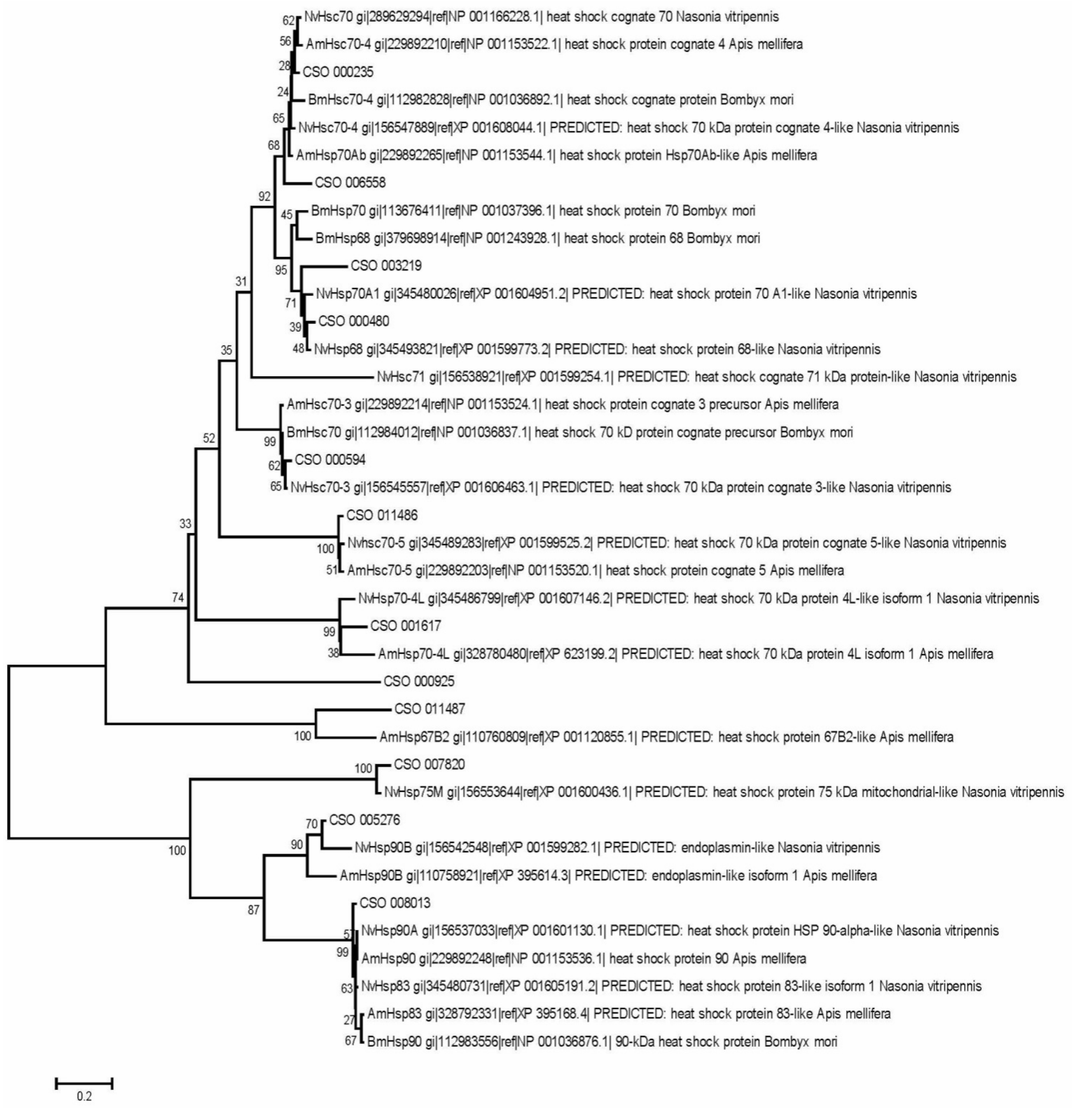

Figure S9. Neighbor-Joining tree of all the *hsp70* and *hsp90* genes in three hymenopteran species.
Notes: *Ceratosolen solmsi* (*CSO_*); *Nasonia vitripennis* (Nv); *Apis mellifera* (Am).

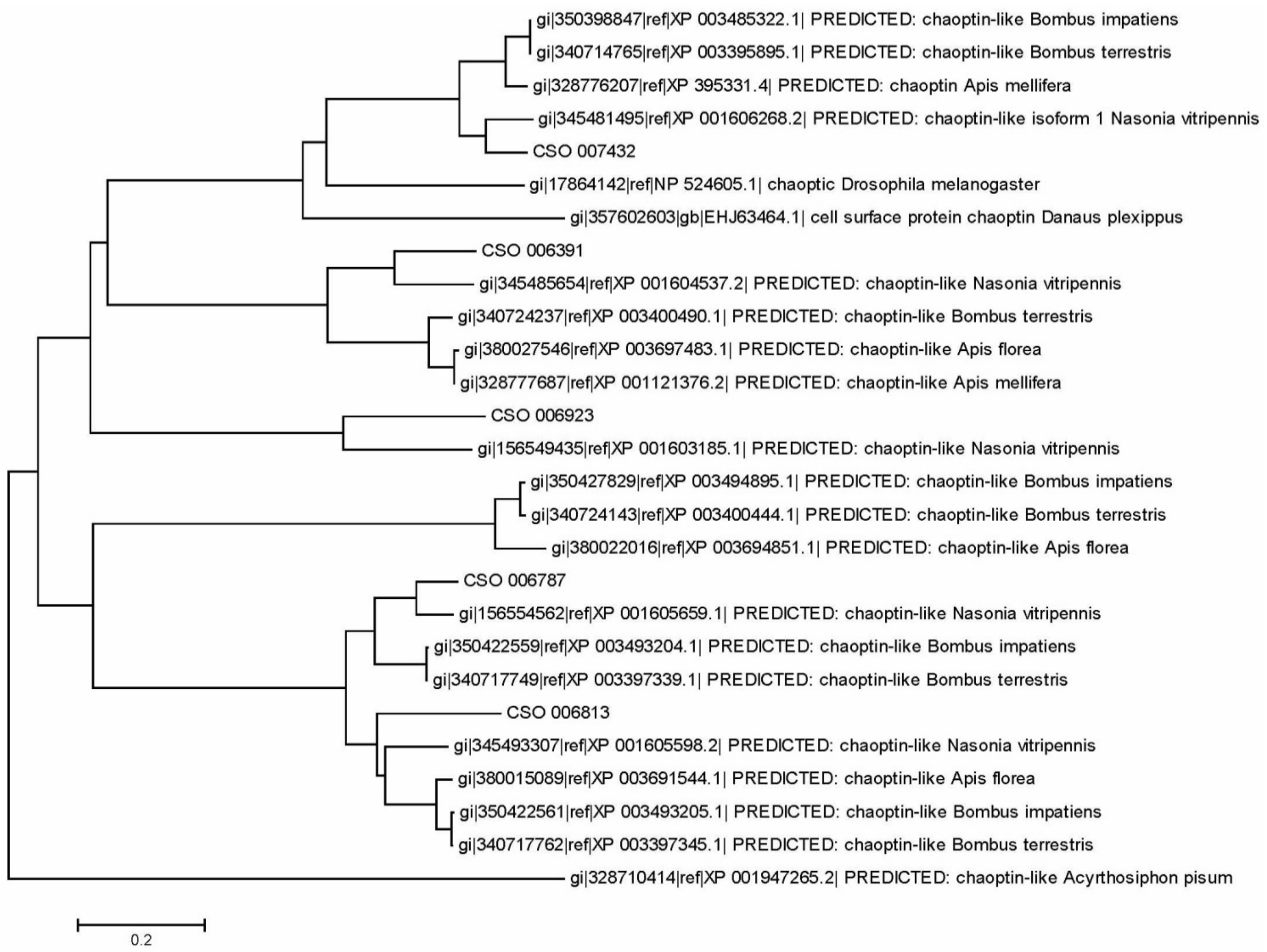

Figure S10. Phylogeny of *chaoptin* in several insect species. *Ceratosolen solmsi* and *Nasonia vitripennis* have multiple duplicates.

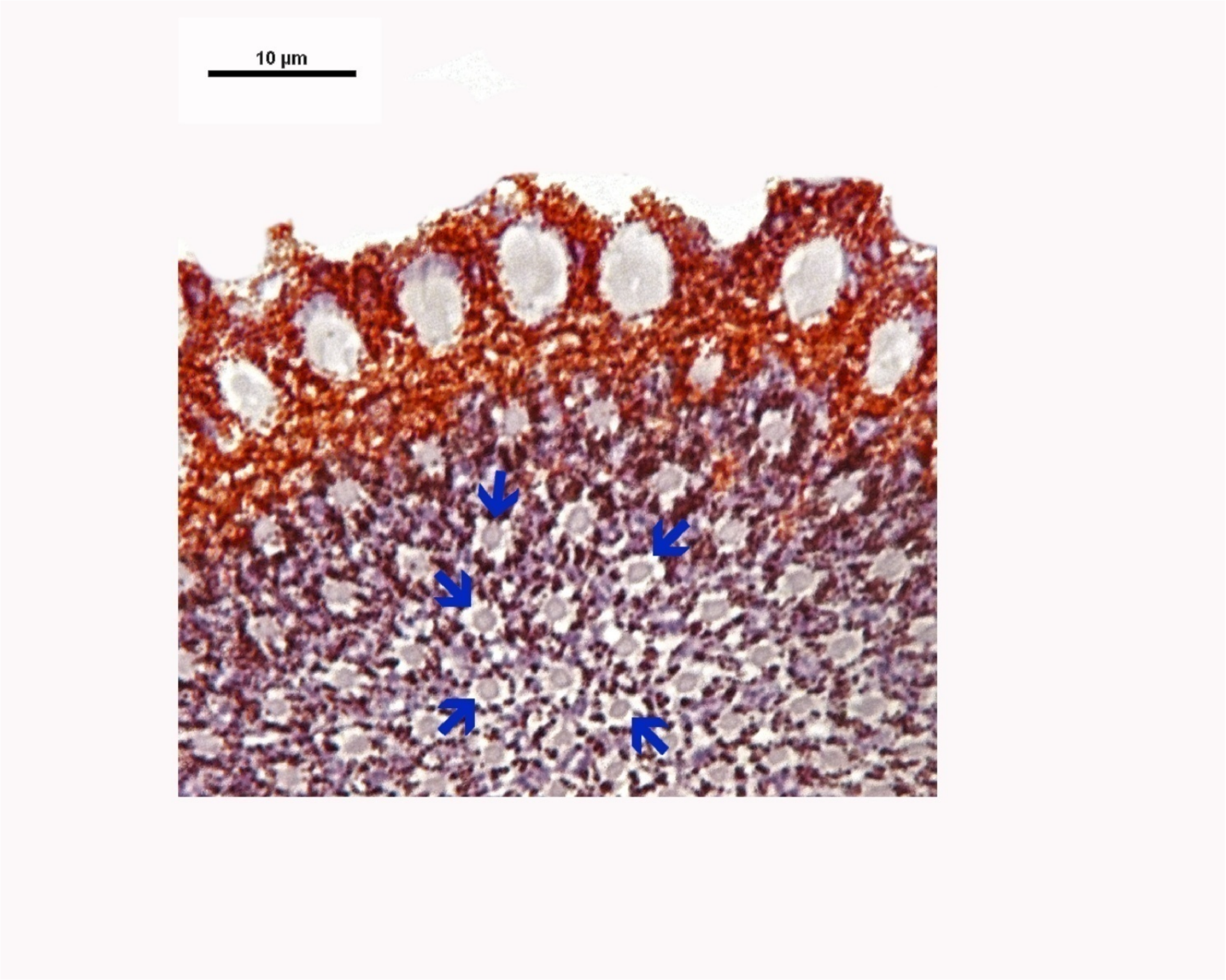

Figure S11. Images of the transverse section of the compound eyes of *Ceratosolen solmsi* (female). Within each ommatidium, the rhabdomeres are fused (arrows).

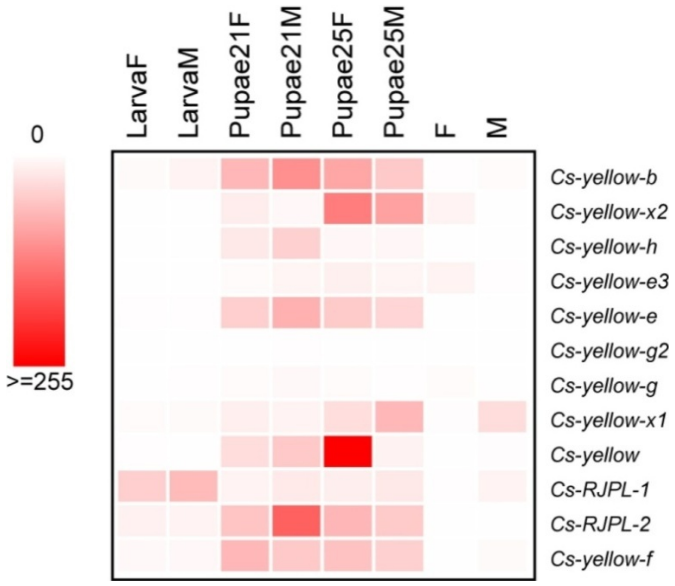

Figure S12. Heatmap of the expression pattern of *yellow*-like genes in fig wasps at four life stages. The bar indicates expression level ranging from zero to higher. Notes: LarvaF: female larva; LarvaM: male larva; Pupae21F: female early pupa; Pupae21M: male early pupa; Pupae25F: female late pupa; Pupae25M: male late pupa; F: female adult; M: male adult.

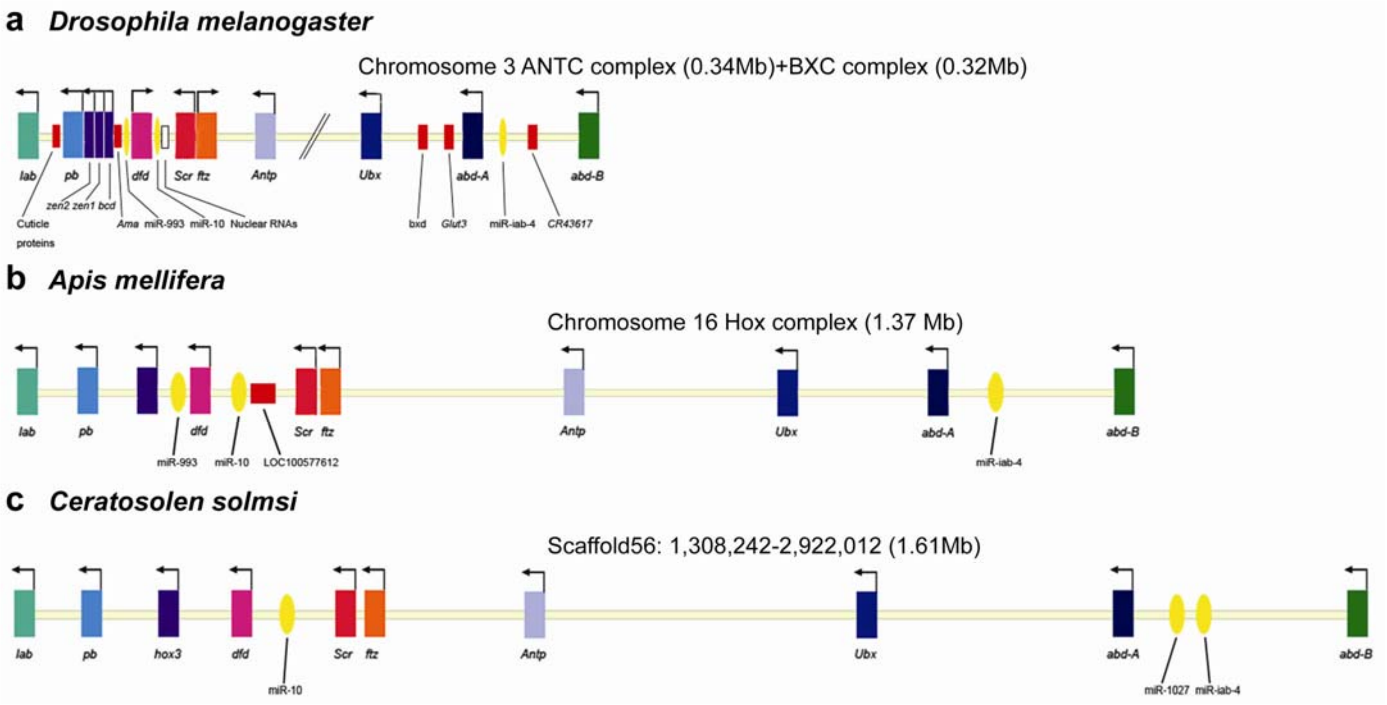

Figure S13. Hox complex structure of *Drosophila melanogaster* (a), *Apis mellifera* (b) and *Ceratosolen solmsi* (c). Black arrows indicate the transcription directions of genes. The complex genes are distributed in two different chromosomes in *Drosophila*.

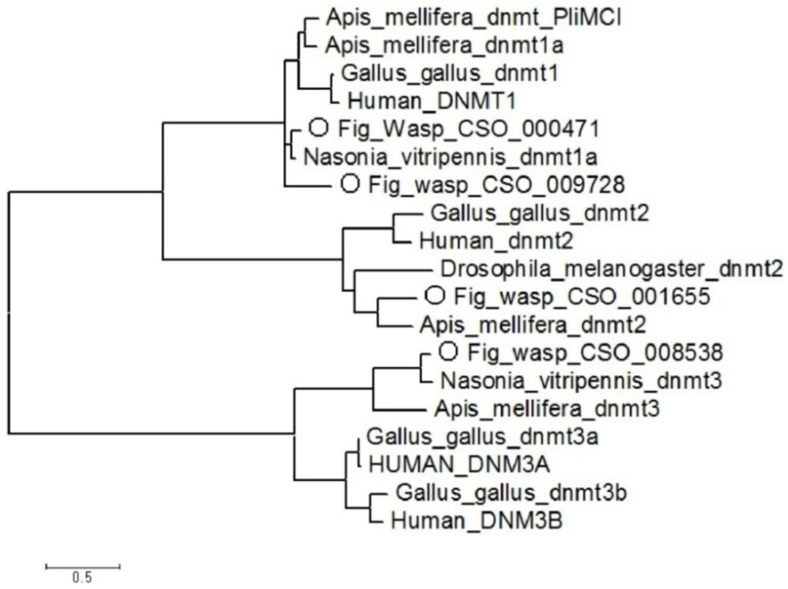

Figure S14. Phylogeny of all *dnmt* genes among vertebrates and invertebrates. The *dnmt* genes in the *Ceratosolen solmsi* are indicated by ○.

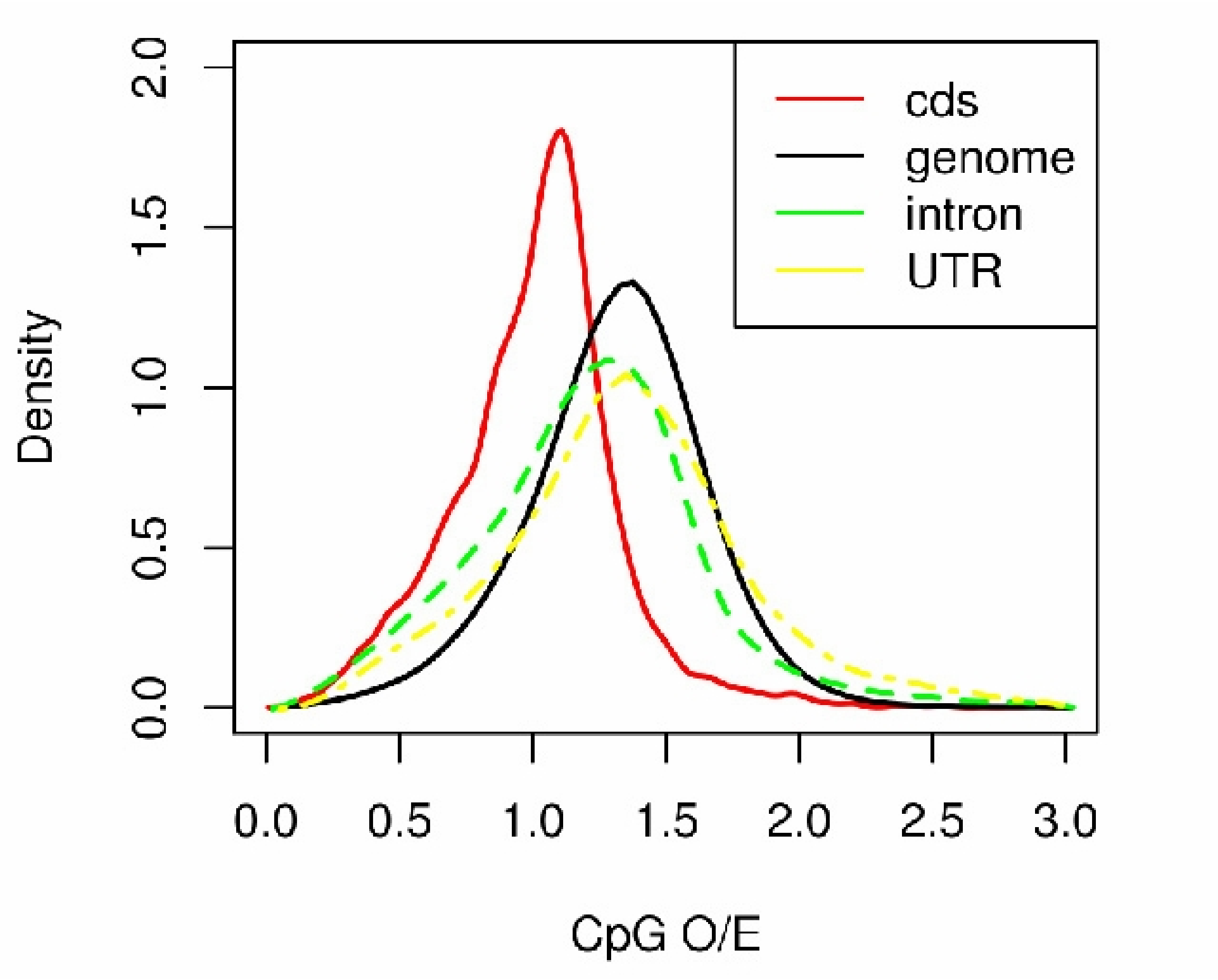

Figure S15. Distribution of CpG o/e in the genome of fig wasps. Gene coding (cds) regions exhibit conspicuously lower CpG o/e compared to other genomic regions.

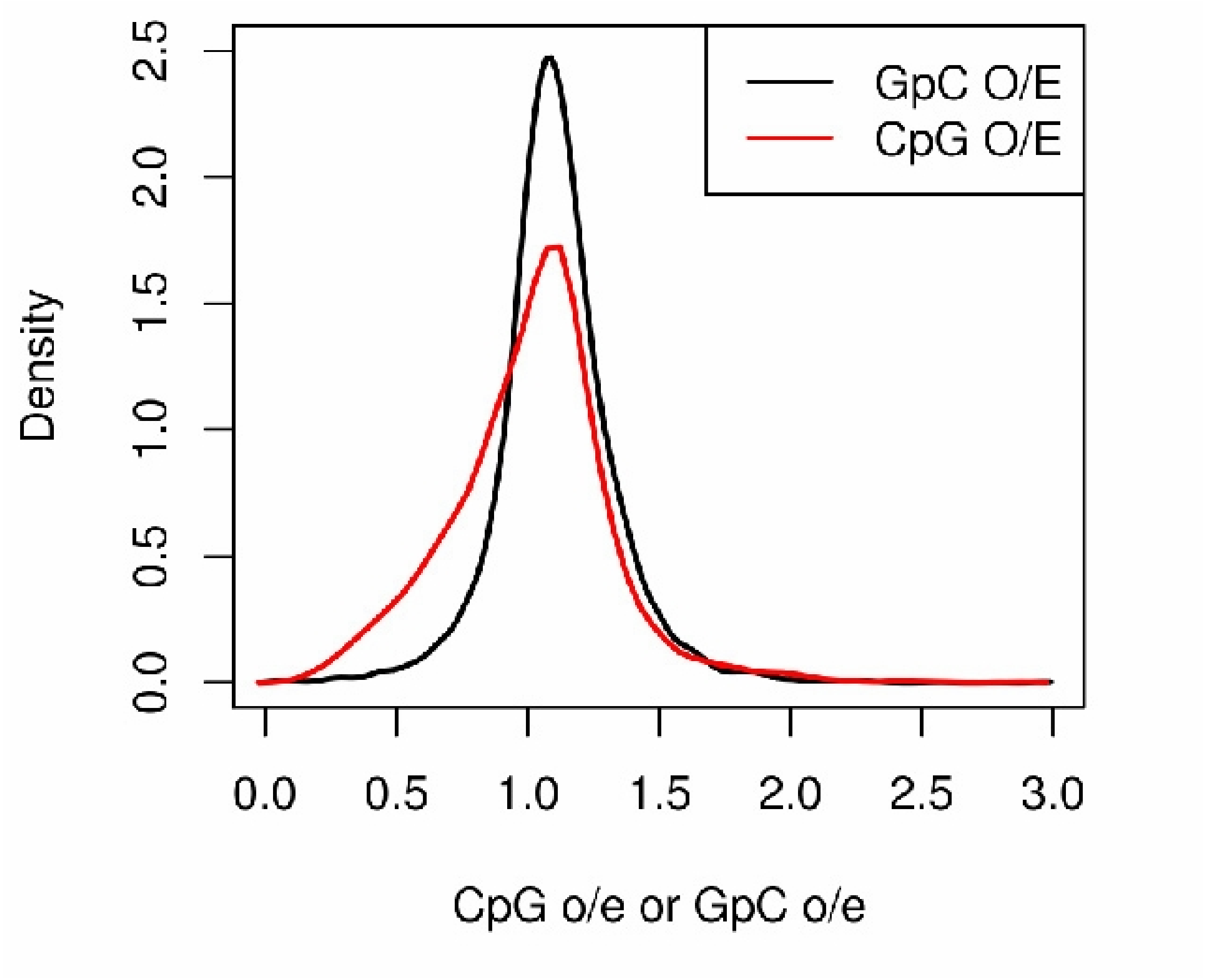

Figure S16. Distribution of CpG o/e and GpC o/e of coding region in fig wasps. Compared to GpC o/e, the distribution of CpG o/e exhibits a characteristic 'fat tail' on the lower CpG o/e range.

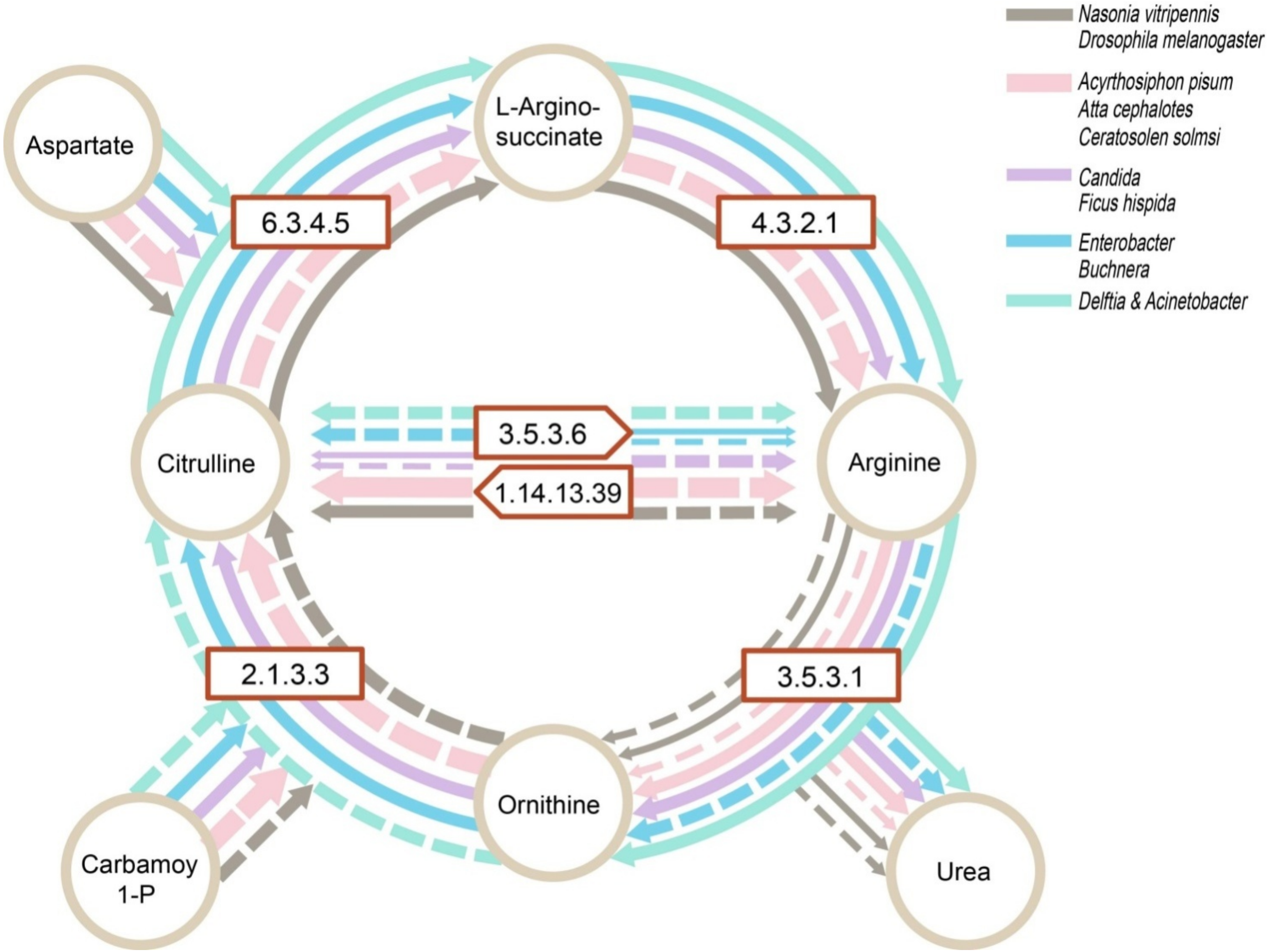

Figure S17. Comparisons of metabolic pathways of amino acid arginine in different organisms. The organisms include: 1) the insects *Nasonia vitripennis*, *Drosophila melanogaster*, *Acyrthosiphon pisum*, *Atta cephalotes*, and *Ceratosolen solmsi*, 2) the preponderant fungi in fig wasp (*Candida*), 3) the host plant *Ficus hispida*, and 4) bacteria including *Enterobacter* (preponderant in fig wasps), *Buchnera* (endosymbiont of aphid), and *Delftia* and *Acinetobacter* (preponderant in fig ovary). Solid arrows indicate presence of a metabolic process and dashed arrows the absence of a metabolic process. Dashed grey line across EC 3.5.3.1 indicates the absence of it in *N. vitripennis*. The dashed pink line across EC 3.5.3.1 indicates the absence of it in *A. pisum*. The thin dashed purple line from arginine to citrulline (from right to left) indicates the absence of EC 1.14.13.39 in *Candida*. The thin dashed blue line from citrulline to arginine (from left to right) indicates the absence of EC 3.5.3.6 in *Buchnera*.

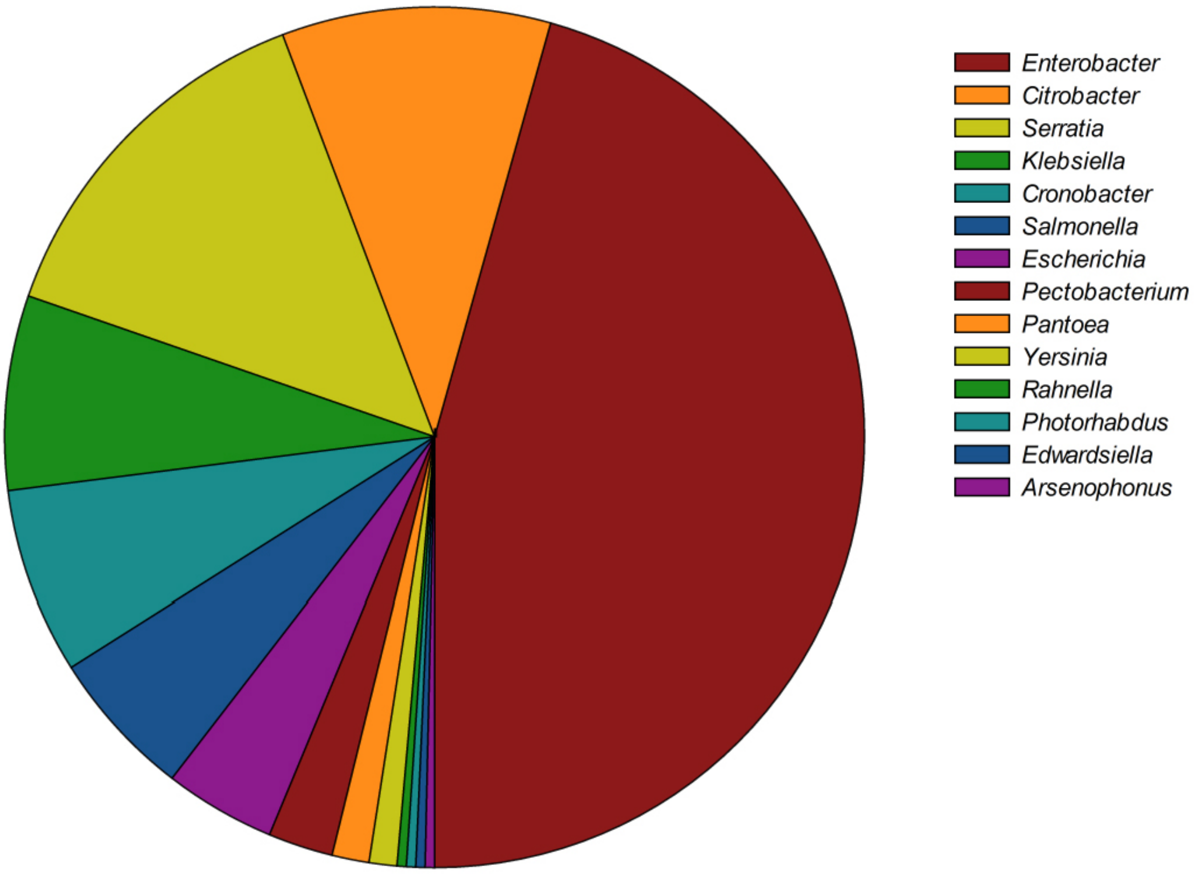

Figure S18. Distribution of bacterial sequences in the fig wasp genome.

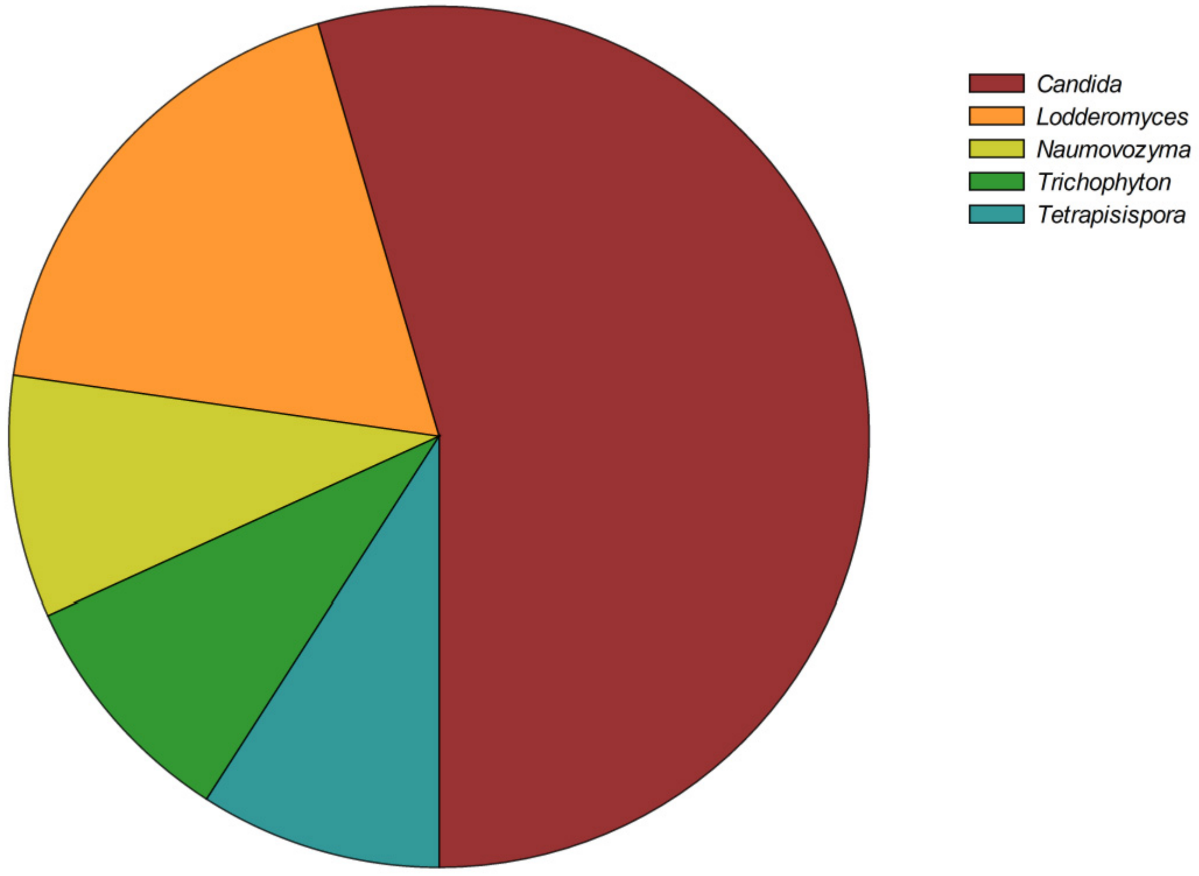

Figure S19. Distribution of fungal sequences in the fig wasp genome.

Supplementary tables

Table S1. Repeats and other genome features comparison among Hymenoptera.

	Repeats contents									Genome feature				
	Repeats	Interspersed	DNA	LTR	LINE	SINE	unclassified	TRF	GC	G-size * (exp.) / Mbp	G-size (assembly) / Mbp	Missing residues ** / Mbp	Repeats (exp.) ***	*Refs.*
Csol	9.37%	6.40%	4.08%	2.58%	1.11%	0.01%	1.79%	3.97%	30.4%	294	278	17.3	14.28%	-
Amel	7.57%	2.58%	1.13%	0.26%	0.17%	0.02%	1.00%	4.99%	33.0%	263	236	32.0	17.06%	*[26]*
Pbar	11.63%	7.93%	2.50%	1.39%	0.26%	0.04%	3.75%	3.70%	36.5%	267	235	46.9	22.22%	*[32]*
Cflo	15.63%	12.40%	2.56%	1.02%	0.64%	0.06%	8.11%	3.23%	34.3%	240	238	15.5	16.33%	*[33]*
Acep	25.10%	21.90%	4.50%	0.68%	0.45%	0.09%	16.30%	3.26%	32.6%	300	290	9.8	27.60%	*[14]*
Hsal	27.52%	25.00%	9.57%	1.53%	1.44%	0.05%	12.41%	2.53%	45.2%	330	297	48.0	34.77%	*[33]*
Lhum	27.34%	10.95%	1.83%	1.08%	0.66%	0.07%	7.91%	2.38%	37.7%	251	216	35.2	37.47%	*[34]*
Aech	34.53%	32.28%	11.99%	9.98%	2.64%	0.10%	7.86%	2.25%	33.7%	324	300	35.5	39.38%	*[35]*
Nvit	31.27%	24.77%	8.11%	8.97%	7.62%	0.07%	0.00%	6.50%	40.6%	332	295	92.2	38.93%	*[13]*

Notes:

*G-size (exp.): Genome size estimated by procedures of K-mers, flow cytometer, etc.

**Missing residues: unassembled data plus N residues in assembly.

***Repeats (exp.): repeats proportion with missing residues in expected genome size.

Table S2. Genome completeness evaluated based on 248 CEGs (Core Eukaryotic Genes).

	#Prots	%Completeness	#Total	Average	%Ortho
Apis mellifera					
Complete	235	94.8	275	1.17	14.0
Group 1	63	95.5	68	1.08	6.4
Group 2	53	94.6	67	1.26	22.6
Group 3	58	95.1	70	1.21	15.5
Group 4	61	93.9	70	1.15	13.1
Partial	247	99.6	313	1.27	21.1
Group 1	66	100	79	1.2	13.6
Group 2	55	98.2	74	1.35	29.1
Group 3	61	100	80	1.31	23.0
Group 4	65	100	80	1.23	20
Camponotus floridanus					
Complete	242	97.6	310	1.28	23.1
Group 1	62	93.9	71	1.15	12.9
Group 2	54	96.4	70	1.3	25.9
Group 3	61	100	82	1.34	29.5
Group 4	65	100	87	1.34	24.6
Partial	248	100	334	1.35	28.2
Group 1	66	100	81	1.23	19.7
Group 2	56	100	78	1.39	33.9
Group 3	61	100	86	1.41	34.4
Group 4	65	100	89	1.37	26.2
Nasonia vitripennis					
Complete	238	96.0	277	1.16	13.9
Group 1	63	95.5	68	1.08	7.9
Group 2	54	96.4	63	1.17	13.0
Group 3	61	100	72	1.18	16.4
Group 4	60	92.3	74	1.23	18.3
Partial	246	99.2	314	1.28	21.1
Group 1	65	98.5	74	1.14	12.3
Group 2	56	100	74	1.32	26.8
Group 3	61	100	79	1.3	21.3
Group 4	64	98.5	87	1.36	25
Ceratosolen solmsi					
Complete	248	100	279	1.12	10.9
Group 1	66	100	70	1.06	6.1
Group 2	56	100	64	1.14	12.5
Group 3	61	100	70	1.15	14.8
Group 4	65	100	75	1.15	10.8
Partial	248	100	292	1.18	14.9

Group 1	66	100	73	1.11	10.6
Group 2	56	100	69	1.23	17.9
Group 3	61	100	71	1.16	16.4
Group 4	65	100	79	1.22	15.4

Notes: "Prots" indicates number of the 248 ultra-conserved CEGs covered by genome. "%Completeness" indicates percentage of CEGs present in genome. "Total" indicates total number of CEGs present including putative orthologs. "Average" indicates average number of orthologs per CEG. "%Ortho" indicates percentage of detected CEGs with more than 1 ortholog. "Complete" indicates number of CEGs with >70% region covered by genome. "Partial" indicates number of CEGs partially covered by assembly.

Table S3. Gene-set completeness assessed by overlaps between insects' gene sets and CEGMA predicted KOG genes.

Type	*Ceratosolen solmsi*		*Nasonia vitripennis*		*Apis mellifera*		*Camponotus floridanus*	
	**Number**	**Percent (%)**	**Number**	**Percent (%)**	**Number**	**Percent (%)**	**Number**	**Percent (%)**
total KOGs	248	-	242		244	-	246	
one KOGs align one gene	219	88.3	219	90.5	233	95.5	240	97.6
CDS overlap > 0.8	118	47.6	202	83.5	215	88.1	209	85.0
CDS overlap > 0.5	176	71.0	213	88.0	226	92.6	228	92.7
one KOGs align more genes	2	-	4	-	10	-	4	-
one KOGs align no gene	27	-	19	-	1	-	2	-

Notes: CDS overlap > 0.8 represents "overlap rate in KOGgenes > 0.8 & overlap rate in genes > 0.8 in CDS level". Total KOGs (Eukaryotic Orthologous Groups) for each species were predicted by aligning KOG genes to genome, then each type of predicted KOG genes in the 4 insect species were counted up in the above table.

Table S4. Coverage of *C. solmsi* transcriptomes and EST data by genome assembly (version 1).

	Dataset	Number	Total length (bp)	Covered by assembly (%)	with >90% sequence in one scaffold		with >50% sequence in one scaffold	
					Number	Percent (%)	Number	Percent (%)
LarvaF	>0bp	56,235	55,811,816	98.5	50,982	90.7	55,544	98.8
	>200bp	45,870	54,036,702	98.5	42,093	91.8	45,399	99.0
	>500bp	27,439	48,339,631	98.7	25,693	93.6	27,267	99.4
	>1000bp	18,691	42,066,161	98.7	17,629	94.3	18,586	99.4
Pupae21F	>0bp	70,472	52,256,891	98.4	63,847	90.6	69,821	99.1
	>200bp	60,758	50,580,515	98.4	55,573	91.5	60,257	99.2
	>500bp	27,987	40,432,576	98.5	26,144	93.4	27,813	99.4
	>1000bp	15,314	31,490,024	98.4	14,347	93.7	15,211	99.3
Pupae25F	>0bp	51,461	35,562,532	98.1	46,853	91.1	50,732	98.6
	>200bp	44,621	34,390,303	98.2	41,048	92.0	44,062	98.8
	>500bp	20,994	26,922,533	98.4	19,715	93.9	20,847	99.3
	>1000bp	10,662	19,618,452	98.3	10,040	94.2	10,590	99.3
female_adult	>0bp	52,387	29,487,392	97.4	45,931	87.7	51,521	98.4
	>200bp	43,566	27,957,404	97.5	38,727	88.9	42,921	98.5
	>500bp	17,080	19,873,839	97.7	15,682	91.8	16,912	99.0
	>1000bp	7,800	13,365,989	97.7	7,210	92.4	7,729	99.1
female_all	>0bp	77,343	70,864,172	97.1	66,868	86.5	75,751	97.9
	>200bp	77,343	70,864,172	97.1	66,868	86.5	75,751	97.9
	>500bp	36,807	58,434,103	97.3	32,536	88.4	36,331	98.7
	>1000bp	21,646	47,745,577	97.3	19,202	88.7	21,389	98.8
LarvaM	>0bp	54,680	55,334,276	98.4	49,575	90.7	53,799	98.4
	>200bp	44,536	53,598,247	98.5	40,874	91.8	43,949	98.7
	>500bp	26,721	48,100,872	98.8	25,099	93.9	26,539	99.3
	>1000bp	18,399	42,127,470	98.7	17,389	94.5	18,271	99.3
Pupae21M	>0bp	66,328	46,350,425	98.4	60,476	91.2	65,769	99.2
	>200bp	56,857	44,730,541	98.4	52,285	92.0	56,408	99.2
	>500bp	25,872	35,020,929	98.5	24,257	93.8	25,720	99.4
	>1000bp	13,420	26,256,029	98.5	12,607	93.9	13,331	99.3
Pupae25M	>0bp	44,510	28,836,203	98.6	41,377	93.0	44,209	99.3
	>200bp	38,723	27,840,484	98.6	36,340	93.9	38,500	99.4
	>500bp	16,966	21,026,981	98.6	16,113	95.0	16,874	99.5
	>1000bp	8,048	14,745,152	98.5	7,643	95.0	8,001	99.4
male_adult	>0bp	61,984	37,931,242	96.1	53,956	87.1	59,259	95.6
	>200bp	52,053	36,214,082	96.4	46,000	88.4	50,118	96.3
	>500bp	20,572	26,518,622	97.1	18,756	91.2	20,206	98.2
	>1000bp	9,620	18,880,358	97.2	8,770	91.2	9,492	98.7
male_all	>0bp	78,143	71,101,748	96.8	67,628	86.5	75,584	96.7
	>200bp	78,143	71,101,748	96.8	67,628	86.5	75,584	96.7

	>500bp	36,981	58,368,125	97.3	32,946	89.1	36,395	98.4
	>1000bp	21,401	47,366,068	97.4	19,202	89.7	21,126	98.7
	>0bp	15,718	8,999,713	93.9	12,323	78.4	15,247	97.0
Sanger_EST	>200bp	15,493	8,965,625	94.0	12,231	79.0	15,062	97.2
	>500bp	12,748	7,856,284	94.4	10,491	82.3	12,409	97.3

Notes: We ran the *blastn* program using transcripts generated from the transcriptome or Sanger EST data as a query against the genome assembly with an E-value cutoff of 1e-5. The number of transcripts that had hits in the genome assembly was divided by the total transcript number for each length range to calculate the coverage percentage. We also evaluated the percentage of transcripts >90% and >50% in length that could be covered by genome assembly. “Covered by assembly (%)” indicates percent of bases in assembled scaffolds covered by EST or unigene when mapping with BLAT. LarvaF, Pupae21F, Pupae25F, female_adult, LarvaM, Pupae21M, Pupae25M, and male_adult indicate the eight transcriptome data used for trinity assembly and tgicl clustering to get unigenes for evaluation. female_all and male_all indicate that the above four sets of female and male unigene sequences were further combined and clustered to evaluate the genome coverage.

Table S5. Repeat classes identified in the *C. solmsi* genome.

Repeat type	Number of elements	Sum length of repeats (bp)	Percentage of assembly (%)
TOTAL	830,720	26,030,093	9.37
**Non-Interspersed Repeats**	117,668	11,021,248	3.97
Simple Repeats	2,064	305,288	0.11
Low Complexity	82,326	6,238,763	2.25
Satellite	33,278	3,080,527	1.11
**Transposable Elements**	713,052	17,782,707	6.40
LTR	62,098	7,160,903	2.58
BEL/Pao	9,274	1,978,285	0.71
Ty1/Copia	6,406	507,275	0.18
Gypsy/DIRS1	21,986	1,999,448	0.72
Retroviral	15,007	1,149,966	0.41
SINEs	584	39,420	0.01
LINEs	37,575	3,069,972	1.11
L2/CR1/Rex	16,490	1,314,422	0.47
R1/LOA/Jockey	6,976	739,435	0.27
R2/R4/DRE	3,942	441,600	0.16
L1/RTE/Bov-B	3,978	411,155	0.15
Penelope	5,273	460,587	0.17
DNA Transposons	255,767	11,328,501	4.08
hAT	47,339	3,625,788	1.31
TcMar	14,108	1,033,073	0.37
MULE/MuDR	28,615	1,992,803	0.72
CMC-EnSpm/Transib/Chapaev	92,380	6,595,108	2.37
Ginger/Helitron	17,968	1,368,260	0.49
PIF/Sola/P/Nonosib	21,903	1,738,970	0.63
Kolobok/Maverick/	21,499	1,720,090	0.62
Other	445	34,085	0.01
Novel	23,439	4,963,308	1.79

Table S6. Annotation of non-coding RNA in the *C. solmsi* genome.

Type	Copy number	Average length (bp)	Total length (bp)	% of Genome
**microRNA**	64	88.44	5660	0.00204
**tRNA**	138	75.32	10394	0.00374
**rRNA**	39	106.44	4151	0.00149
18S	19	141.79	2694	0.00097
28S	3	64	192	0.00007
5.8S	2	55	110	0.00004
5S	15	77	1155	0.00042
**small nuclear RNA**	19	128.53	2442	0.00088
CD-box	7	95.29	667	0.00024
HACA-box	0	0	0	0
Splicing	12	147.92	1775	0.00064

Table S7. The distinct enriched GO terms of *Ceratosolen solmsi* genes in expanded families.

GO term	Function	P value
GO:0048854	brain morphogenesis	0.00014
GO:0001964	startle response	0.00369
GO:0031987	locomotion involved in locomotory behavior	0.00658
GO:0050905	neuromuscular process	0.00658

Table S8. Detailed information about the thirteen putatively rapidly evolved genes in fig wasp.

Gene ID	Family ID	Putative function
CSO_000287	14667	transmembrane protein 164-like
CSO_001112	14534	probable ribosome biogenesis protein C16orf42 homolog
CSO_001115	527	zinc finger protein 616-like; may be involved in transcriptional regulation
CSO_001724	6114	tetraspanin-11-like; organizer of membrane-signaling complexes
CSO_001764	7604	hippocampus abundant transcript 1 protein-like; sugar transporter family
CSO_003345	10406	CKLF-like MARVEL transmembrane domain-containing protein 4-like; belongs to chemokine-like factor gene superfamily
CSO_003635	1073	Nasonia vitripennis hypothetical protein LOC100114982
CSO_003961	4764	rac GTPase-activating protein 1-like; controlling the activity of G proteins
CSO_004030	14686	Nasonia vitripennis hypothetical protein LOC100118458
CSO_005423	10175	probable 26S proteasome non-ATPase regulatory subunit 3-like
CSO_005676	7907	Rho guanine nucleotide exchange factor 2 (RhoGEF2); act contrary to rac GTPase-activating protein 1
CSO_006481	11427	lipid storage droplets surface-binding protein 1-like; activation of lipolysis
CSO_010886	13640	probable alpha-ketoglutarate-dependent dioxygenase ABH6-like

Table S9. Comparisons on the evolutionary rates of the Gr and Or genes with Single-Copy Orthologs (SCO).

	Ceratosolen solmsi	*Nasonia vitripennis*	*Apis mellifera*
Gr	0.153	0.067	0.011
Or	0.089	0.044	0.045
Gr+Or	0.096	0.045	0.040
SCO	0.039	0.026	0.028
paired wilcoxion rank sum tests	P=0.0156	P=0.573	P=0.811

Table S10. Comparisons of the detoxification genes in several arthropod species.

	C. solmsi	*N. vitripennis*	*A. mellifera*	*D. melanogaster*	*A. gambiae*	*A.pisum*	*T.castaneum*	*B.mori*	*T.urticae*
**GSTs**									
Sigma	4	8	4	1	1	6	7	2	0
Delta	4	5	1	11	12	10	3	4	16
Epsilon	0	0	0	14	8	0	19	8	0
Omega	2	2	1	5	1	0	3	4	2
Zeta	1	1	1	2	1	0	1	2	0
Theta	0	3	1	4	2	2	1	1	1
other	0	0	0	0	0	2	0	2	12
Cytosolic GST total	11	19	8	37	28	20	34	23	31
**P450**									
CYP4	10	30	4	32	45	32	44	32	23
CYP3	11	48	28	36	42	32	72	36	10
CYP2	7	7	8	6	10	10	8	10	48
Mitochondrial CYPs	6	7	6	11	9	8	9	8	5
P450 total	34	92	46	85	106	82	133	86	86
**CCEs**									
Dietary class									
A clade	3	8	5	0	0	5	0	32	0
B clade	1	5	3	13	16	0	14	12	0
C clade	0	0	0	0	0	0	12	7	0
Hormone/semiochemical processing									
D clade	0	4	1	3	0	0	2	5	0
E clade	1	11	2	2	4	18	7	2	0
F clade	1	2	2	3	6	0	2	2	0
F' clade	0	0	0	0	0	0	0	0	2
G clade	0	0	0	0	4	0	0	2	0
Neuro/developmental									
H clade	1	1	1	5	10	0	1	1	2

I clade	1	1	1	1	1	1	1	1	0
J clade	2	2	2	1	2	2	2	1	1
K clade	1	1	1	1	1	1	1	1	1
L clade	5	5	5	4	5	3	5	1	5
M clade	1	1	1	2	2	0	2	1	1
N clade	0	0	0	0	0	0	0	1	0
J' clade	0	0	0	0	0	0	0	0	34
J'' clade	0	0	0	0	0	0	0	0	22
other	0	0	0	0	0	0	0	0	3
CCEs total	17	41	24	35	51	30	49	69	71

Notes: *C. solmsi*: *Ceratosolen solmsi*; *N. vitripennis*: *Nasonia vitripennis*; *A. mellifera*: *Apis mellifera*; *D. melanogaster*: *Drosophila melanogaster*; *A. gambiae*: *Anopheles gambiae*; *A.pisum*: *Acyrthosiphon pisum*; *T.castaneum*: *Tribolium castaneum*; *B.mori*: *Bombyx mori*; *T.urticae*: *Tetranychus urticae*. Data referred to references [36-42] .

Table S11. Comparison of the innate immune gene members in seven insect species.

Gene name	Pathway	*D. melanogaster*	*T. urticae*	*D. pulex*	*A. pisum*	*A. mellifera*	*N. vitripennis*	*C. solmsi*
**TOTAL**		**139**	**48**	**67 to 73**	**70**	**85**	**132**	**89**
Number of AMPs		20	--	--	--	7	37	8
attacins	Effector/AMP	NP_523745.1						
		NP_523746.1						
		NP_523729.3						
		NP_524391.2						
abaecin	Effector/AMP	NP_523752.1				NP_001011617.1	NP_001171239.1	CSO_000345
(metchnikowin)							NP_001171240.1	
							XP_003425826.1	
navitripenicin	Effector/AMP						AEO53066.1	
hymenoptaecin	Effector/AMP					NP_001011615.1	NP_001165829.1	
							NP_001234886.1	
defensin	Effector/AMP	NP_523672.1				NP_001011616.2	NP_001159944.1	
						NP_001011638.1	NP_001159943.1	
							XP_001605239.1	
nasonin	Effector/AMP						NP_001171934.1	CSO_001253
							NP_001171933.1	CSO_002891
							NP_001171237.1	CSO_011520
							XP_001607888.1	
							NP_001171236.1	
							XP_001605675.1	
							XP_001599338.1	
							XP_001608211.1	
							XP_001607446.1	
							XP_001599336.1	
							XP_001602681.1	
							nasonin10	
							nasonin12	

navitricin	Effector/AMP						XP_001607644.1	
							XP_001607635.1	
naickin	Effector/AMP					amickin1	XP_001600083.1	CSO_008653
							XP_001599718.1	CSO_008654
							Naickin3	
glynavicin	Effector/AMP						XP_001600869.2	CSO_011521
							XP_001607541.1	
							XP_001607559.1	
							XP_001600805.1	
hisnavicin	Effector/AMP						XP_001606568.1	CSO_004750
							NP_001166274.1	
							XP_001599590.1	
nahelixin	Effector/AMP	NP_524588.1				NP_001011607.1	XP_001607338	
(cecropin)		NP_524589.1						
		NP_524590.1						
		NP_524591.1						
apisimin	Effector/AMP					NP_001011582.1		
diptericin	Effector/AMP	NP_476808.1						
		NP_523787.2						
drosocin	Effector/AMP	NP_523744.1					XP_001599539.1	
drosomycin	Effector/AMP	NP_523901.1					XP_001599338.1	
		NP_647803.1						
		NP_728860.2						
		NP_728873.1						
		NP_728872.1						
		NP_728862.1						
		NP_728861.1						
thaumatin	Effector/AMP				6			
mcr	Cellular response/effector	NP_524688.1	1	1 to 4	1	XP_397416.3	XP_001608105.1	CSO_007911
TEPs	Cellular	NP_523578.1	1	3 to 6	2	XP_001122599.2	XP_001599750.2	CSO_002367

	response/effector	NP_523506.1				XP_392454.3	XP_001604193.2	CSO_003220
		NP_523507.2						
		NP_523603.2						
		NP_609988.3						
turandots	Cellular	NP_788077.2						
	response/Effector	NP_536780.3						
		NP_536782.2						
		NP_524422.1						
		NP_536781.2						
		NP_536779.2						
		NP_523482.1						
		NP_536778.2						
alk	Cellular response/Effector	NP_652600.1	1	1	1	XP_392254.4	XP_001602929.2	CSO_002201
croquemort	Cellular response/Effector	NP_787957.1			1	XP_392321.3	XP_001604561.2	CSO_011514
draper	Cellular response/Effector	NP_477450.1	2	1	1	XP_624855.2	XP_001606322.2	CSO_009826
eater	Cellular	NP_651533.3				XP_001120277.2	XP_001607181.1	CSO_006556
	response/Effector					XP_001121267.2	XP_003425307.1	CSO_006555
						XP_394098.4		
hemese	Cellular response/Effector	NP_652734.2						
hemolectin	Cellular response/Effector	NP_524060.2		1	1	XP_395067.4		CSO_003931
nimA	Cellular response/Effector	NP_001246026.1				XP_001120328.1	XP_003425333.1	CSO_006554
phl	Cellular response/Effector	NP_525047.1	1	1	1	XP_396892.2	XP_001605107.1	CSO_000691
pnt	Cellular	NP_524461.2	1	1	1	XP_003250677.1		CSO_008744

	response/Effector							
pvr	Cellular response/Effector	NP_523509.2	1	1	1	XP_396748.4	XP_001600926.2	CSO_006860
rac	Cellular response/Effector	NP_476950.1<br>NP_648121.1	1	1	1	XP_623951.1		
scavenger-receptor C	Cellular response/Effector	NP_477102.1<br>NP_524720.2<br>NP_524747.1<br>NP_608789.1	4	2	1	XP_394726.3	XP_001603920.2	CSO_001215
serpent	Cellular response/Effector	NP_732100.2				XP_001121273.2	XP_003426466.1	CSO_006092
lysozyme	Cellular response/Effector	NP_523881.1<br>NP_476829.1<br>NP_476828.1<br>NP_523882.1<br>NP_476827.2<br>NP_476823.1<br>NP_524869.1			3	XP_003249674.1<br>XP_001120136.1<br>XP_393161.2	XP_001600829.2<br>XP_001607428.1	CSO_001249<br>CSO_009616
chitinase	Cellular response/Effector	NP_001036422.1<br>NP_477298.2<br>NP_524962.2			7	XP_397146.3<br>XP_396925.3<br>XP_395734.4<br>XP_395707.4<br>XP_623995.1<br>XP_623744.2<br>XP_001120887.1<br>XP_395372.3	NP_001155084.1<br>NP_001128139.2<br>XP_001604515.2<br>XP_001600140.2<br>XP_001606158.2<br>XP_001604954.1<br>XP_001601416.2<br>XP_001601382.1<br>XP_001599305.1<br>XP_003428114.1<br>XP_003428113.1<br>XP_001601999.2	CSO_003912<br>CSO_010053<br>CSO_005254<br>CSO_005611<br>CSO_006238<br>CSO_006239<br>CSO_006392<br>CSO_007043<br>CSO_011518<br>CSO_009469

ppo	Cellular response/Effector	NP_476812.1			2	NP_001011627.1	NP_001164357.1	CSO_002243
		NP_610443.1					XP_001606582.2	CSO_002244
		NP_524760.1					NP_001164331.1	
PGRPs	Recognition	NP_573078.1	1			NP_001157188.1	XP_001605218.1	CSO_005815
		NP_729468.2				NP_001157187.1	XP_001603488.1	CSO_011503
		NP_572727.1				XP_392452.2	XP_001601870.1	CSO_007959
		NP_650079.1				XP_001121036.2	XP_003427753.1	CSO_007960
		NP_648917.1					XP_003427765.1	CSO_007961
		NP_648145.1					XP_003423873.1	CSO_007962
		NP_610409.1					XP_003427766.1	
		NP_610410.1					NP_001164436.1	
		NP_648299.3					NP_001164440.1	
		NP_610407.1					NP_001164435.1	
		NP_996028.1					NP_001164439.1	
		NP_648916.1						
		NP_001027113.1						
GNBPs	Recognition	NP_524142.2		11	2	NP_001157186.1	NP_001155149.1	
		NP_730350.1				XP_001121634.2	XP_003424242.1	
		NP_523986.2					XP_001600843.1	
cactin	TOLL	NP_523422.3	1	1	1	XP_624972.3	XP_003425945.1	CSO_004392
cactus	TOLL	NP_723960.1	1	1	1	NP_001157184.1	XP_001603027.1	CSO_010541
						XP_394485.2	XP_003427510.1	CSO_010540
						XP_001121575.2	XP_001603141.1	
DIF	TOLL	NP_523589.2						
MyD88	TOLL	NP_610479.1	1	1	1	XP_396644.4	XP_001602490.1	CSO_001021
dorsal	TOLL	NP_724052.1	1	1	1	NP_001011577.1	XP_001602675.2	CSO_003075
						XP_395180.4	XP_001602435.2	CSO_003125
							XP_001603465.2	CSO_003126
							XP_003427515.1	CSO_003128
gprk2	TOLL	NP_476867.1	1	1	1	XP_394109.3	XP_001606366.1	CSO_004715
grass	TOLL	NP_651543.1	1	1				

necrotic	TOLL	NP_524851.1	1					
pelle	TOLL	NP_476971.1	2	1	1	XP_624002.3	XP_001599847.1	CSO_005099
							XP_001601124.2	
pellino		NP_524466.1	1	1	1	XP_392595.4	XP_001603151.2	CSO_002853
							XP_001601292.3	
persephone	TOLL	NP_573297.1						
serpin 27A	TOLL	NP_652024.1			1	XP_001122067.2	XP_001602351.1	CSO_000973
spaetzle	TOLL	NP_524526.1	6	7	6	XP_001121213.1	XP_001606369.2	CSO_000137
		NP_729010.1				XP_391869.3	XP_001607462.2	CSO_000569
		NP_609160.2					XP_001605307.1	CSO_000907
		NP_609504.2					XP_001599503.2	CSO_008943
		NP_647753.1					XP_001604933.2	CSO_009830
		NP_611961.1					XP_001606529.2	CSO_010856
SPE	TOLL	NP_651168.1						
spheroide	TOLL	NP_573148.2						
sphinx1	TOLL	NP_729255.2						
sphinx2	TOLL	NP_729256.2						
spirit	TOLL	NP_001162707.1	1					
tolls	TOLL	NP_524518.1	4	7	7	XP_396158.1	XP_001604577.1	CSO_000560
		NP_476814.1				NP_001013379.1	XP_001604871.2	CSO_000592
		NP_649719.2				XP_393712.2	XP_003424883.1	CSO_003732
		NP_523519.2				XP_393713.2	XP_001604880.2	CSO_004035
		NP_477438.1				XP_393717.2	XP_003424932.1	CSO_005169
		NP_524081.1				XP_001120678.1	XP_003425317.1	CSO_007948
		NP_523797.1					XP_001603014.1	CSO_007952
		NP_524757.1					XP_001601629.2	
		NP_649214.1						
tube	TOLL	NP_001189164.1			1	XP_001121229.2	XP_001606360.1	CSO_011517
traf6 (traf2)	TOLL/JNK	NP_511080.2				XP_624207.3		
eiger	JNK	NP_724878.2		1	1			
hemipterous	JNK	NP_727661.1	1	1	1	XP_396834.1	XP_001604642.2	CSO_000425

JRA (d-Jun)	JNK	NP_476586.1		1	1	XP_003251036.1	XP_003427246.1	CSO_009661
wengen	JNK	NP_728186.1		1		XP_001122675.2		
basket/JNK	IMD / JNK	NP_723541.1	1	1	1	XP_392806.3	XP_003424073.1	CSO_000516
TAB2	IMD / JNK	NP_611408.2				XP_001122664.2	XP_003424901.1	CSO_005086
TAK1	IMD / JNK	NP_524080.1	1	1	1	XP_397248.4	XP_001604249.1	
ird5	IMD / JNK	NP_524751.3		1	1	XP_623135.2		CSO_000625
kayak	IMD / JNK	NP_001027579.1	1	1		GB12212	XP_001601050.2	CSO_010293
kenny	IMD / JNK	NP_523856.2		1				
ankyrin	IMD	NP_787122.1	1	1	1	XP_397331.2	XP_001608224.2	CSO_001440
						XP_392578.4	XP_001601419.1	CSO_004204
							XP_003424459.1	CSO_007993
							XP_003427955.1	
							XP_003424981.1	
caspar	IMD	NP_611080.1		1	1	XP_392750.1	XP_001600992.1	CSO_011516
FADD	IMD	NP_651006.1				XP_003251873.1		CSO_010848
dnr1	IMD	NP_611680.2		1	1	XP_396349.2		
dredd	IMD	NP_477251.3				XP_001120830.2	XP_003425038.1	CSO_002577
IAP2	IMD	NP_477127.1	1	1	1	XP_396819.2	XP_001606042.2	CSO_002117
IMD	IMD	NP_573394.1		1		NP_001157189.1	NP_001135910.1	CSO_006889
relish	IMD	NP_477094.1	1	1		XP_624626.3	XP_001602212.2	CSO_002757
sick	IMD	NP_001246097.1	1	1	1			
ubc13	IMD	NP_511150.1	1	1	1	XP_392901.2	XP_001606785.1	CSO_001570
uev1A	IMD	NP_647959.1	1	1	1	XP_393411.1	XP_001607297.1	CSO_005911
domeless	JAK/STAT	NP_523412.1	1	1	1	XP_003251700.1	XP_003425467.1	CSO_008608
hopscotch/JAK	JAK/STAT	NP_511119.2	1	1	1	XP_001121783.2	XP_001602854.2	CSO_002323
STAT92E	JAK/STAT	NP_996243.1	1	1	1	XP_397181.1	XP_001605495.2	CSO_003747
unpaired3 (UPD3)	JAK/STAT	NP_001097014.1						

Notes: *D. melanogaster*: *Drosophila melanogaster*; *T.urticae*: *Tetranychus urticae*; *D. pulex*: *Daphnia pulex*; *A.pisum*: *Acyrthosiphon pisum*; *A. mellifera*: *Apis mellifera*; *N. vitripennis*: *Nasonia vitripennis*; *C. solmsi*: *Ceratosolen solmsi*. For species of *T. urtecae*, *D.pulex*, and *A.pisum*, we only indicate the number of gene members with gene IDs not provided. References [40, 43].

Table S12. Comparison of sex biased gene expression patterns through four life stages of fig wasp (*Drosophila* as control).

		expressed gene number	sex differentiated gene number	sex differentiated gene percent (%)	over-expressed gene number (percent %)	down regulated gene number (percent %)
Larva	Female	8887	1412	15.9	693(7.8)	719(8.1)
	Male	8875	1412	15.9	719(8.1)	693(7.8)
Pupae21	Female	9682	1859	19.0	1290(13.3)	569(5.9)
	Male	9603	1859	19.4	569(5.9)	1290(13.4)
Pupae25	Female	9365	4932	52.7	4414(47.1)	518(5.5)
	Male	9202	4932	53.6	518(5.6)	4414(48.0)
Adult	Female	9007	6168	68.5	4165(46.2)	2003(22.2)
	Male	9332	6168	66.1	2003(21.5)	4165(44.6)
Drosophila	Female	10513	4586	43.6	1809(17.2)	2777(26.4)
	Male	12196	4586	37.6	2777(22.8)	1809(14.8)

Notes: the analysis for *Drosophila* is based on the RNA-Seq data of the adult abdomen of *Drosophila willistoni* (Data accession number is GSE31723: GSM787643, *D. willistoni* female abdomen rep1; GSM787644, *D. willistoni* male abdomen rep1; Reference [44]). LarvaF: female larva; LarvaM: male larva; Pupae21F: female early pupa; Pupae21M: male early pupa; Pupae25F: female late pupa; Pupae25M: male late pupa; F: female adult; M: male adult.

Table S13. The distinct enriched GO functions of sexually divergently expressed genes in different samples.

**Up-regulated genes in larval male**
oxidoreductase activity
**Down-regulated genes in larval male**
N/A
**Up-regulated genes in pupal21 male**
catalytic activity
oxidoreductase activity
**Down-regulated genes in pupal21 male**
gated channel activity
ion channel activity
substrate-specific channel activity
channel activity
passive transmembrane transporter activity
receptor activity
signaling receptor activity
extracellular ligand-gated ion channel activity
cation channel activity
ligand-gated ion channel activity
ligand-gated channel activity
signal transducer activity
molecular transducer activity
G-protein coupled receptor activity
transmembrane signaling receptor activity
substrate-specific transmembrane transporter activity
transmembrane transporter activity
substrate-specific transporter activity
excitatory extracellular ligand-gated ion channel activity
transporter activity
ion transmembrane transporter activity
**Up-regulated genes in pupal25 male**
N/A
**Down-regulated genes in pupal25 male**
structural constituent of ribosome
helicase activity
RNA binding
translation initiation factor activity
translation factor activity, nucleic acid binding
DNA-dependent ATPase activity
ATP-dependent helicase activity
purine NTP-dependent helicase activity
ATP binding

nucleotide binding
nucleoside phosphate binding
adenyl nucleotide binding
adenyl ribonucleotide binding
**Up-regulated in adult male**
transporter activity
transmembrane transporter activity
calcium ion binding
substrate-specific transmembrane transporter activity
ion transmembrane transporter activity
sequence-specific DNA binding
metal ion transmembrane transporter activity
**Down-regulated in adult male**
structural constituent of ribosome
translation factor activity, nucleic acid binding
translation initiation factor activity
nucleotide binding
nucleoside phosphate binding
RNA binding
small molecule binding
helicase activity
purine ribonucleoside triphosphate binding
ribonucleotide binding
purine ribonucleotide binding
structural molecule activity
aminoacyl-tRNA ligase activity
ligase activity, forming carbon-oxygen bonds
ligase activity, forming aminoacyl-tRNA and related compounds
DNA-directed RNA polymerase activity
RNA polymerase activity
ATP-dependent helicase activity
purine NTP-dependent helicase activity
nucleotidyltransferase activity
organic cyclic compound binding
heterocyclic compound binding
ATP binding
adenyl nucleotide binding
adenyl ribonucleotide binding

Table S14. *Blastn* search of the fig wasp genome with all bacteria database.

Scaffold	Hit count*	Hit overlaps** (bp)	Top genus_name***	Best_bact_len****	Bact_score*****
scaffold53	20	4266	*Cupriavidus*	591	68
scaffold58	12	2065	*Mycoplasma*	255	24
scaffold506	7	7388	*Methylotenera*	1509	200
scaffold93	4	1426	uncultured	668	42
scaffold5	4	715	*Hamiltonella*	266	21
scaffold687	4	1920	*Pectobacterium*	1136	105
scaffold108	3	1502	*wPip*	516	64
scaffold90	2	361	*Flavobacterium*	194	27
scaffold799	2	5111	*Yersinia*	1333	200
scaffold22	1	118	*Methanosarcina*	118	24
scaffold113	1	105	*Methanothermococcus*	105	8
scaffold860	1	1295	*Rahnella*	1295	39

Notes:

*Hit count, Number of independent HSP bacterial regions found in the scaffold.

**Hit overlaps (bp), Total number of base-pairs under a bacterial-like HSP.

***Top genus name, Name of the bacteria with the lowest eval to the scaffold.

****Best_bact_len , Length of the best (lowest e-value) bacterial HSP.

*****Bact_score, The e-value of the best bacterial blastn HSP, to help with sorting in excel the e-values is converted so that only the exponent is shown as a positive number (e-value of 0 is made “200” by default).

Table S15. Manual annotation of *hsp* genes in the fig wasp genome.

ID	Gene description
**Hsp10**	
CSO_011485	10 kDa heat shock protein, mitochondrial-like
**Hsp20|HspB**	
CSO_002027	protein lethal(2)essential for life-like, small heat shock protein
CSO_006190	protein lethal(2)essential for life-like, small heat shock protein
CSO_007405	heat shock protein beta-1-like
CSO_006191	protein lethal(2)essential for life-like
CSO_006192	protein lethal(2)essential for life-like
**Hsp40|DnaJ**	
CSO_000056	dnaJ homolog subfamily B member 11-like
CSO_001219	cysteine string protein-like isoform 2
CSO_001285	dnaJ homolog subfamily C member 21-like
CSO_003932	dnaJ homolog subfamily C member 1-like
CSO_011489	dnaJ homolog subfamily B member 13-like
CSO_004335	dnaJ protein homolog 1-like
CSO_004827	dnaJ homolog subfamily A member 1-like
CSO_005475	dnaJ homolog subfamily C member 24-like
CSO_011491	dnaJ homolog subfamily C member 11-like
CSO_006732	dnaJ homolog subfamily C member 22-like
CSO_006955	dnaJ homolog subfamily B member 6-like
CSO_011492	dnaJ homolog subfamily C member 8-like
CSO_007456	dnaJ homolog subfamily C member 16-like
CSO_008147	DnaJ-like protein subfamily C member 14
CSO_011494	dnaJ homolog subfamily C member 18-like
CSO_008323	dnaJ homolog subfamily B member 12-like
CSO_008616	dnaJ homolog subfamily C member 2-like
CSO_008781	dnaJ homolog subfamily C member 17-like
CSO_008874	dnaJ homolog subfamily C member 10-like
CSO_009802	J domain-containing protein CG6693
CSO_010434	dnaJ homolog subfamily C member 13
CSO_010619	dnaJ homolog subfamily C member 3-like
CSO_010897	protein tumorous imaginal discs, mitochondrial-like
CSO_011493	dnaJ homolog subfamily C member 30-like
CSO_011126	dnaJ homolog subfamily C member 7-like
CSO_000624	translocation protein SEC63 homolog
CSO_005635	Cyclin G-associated kinase
CSO_009078	J domain-containing protein-like
**Hsp60**	
CSO_011484	60 kDa heat shock protein, mitochondrial-like
**Hsp70**	
CSO_000235	heat shock cognate 70 protein

CSO_000480	heat shock protein 68-like
CSO_000594	heat shock 70 kDa protein cognate 3-like
CSO_000925	hypoxia up-regulated protein 1-like, HSP70
CSO_003219	heat shock protein 70 A1-like
CSO_011486	heat shock 70 kDa protein cognate 5-like
CSO_006558	heat shock protein Hsp70Ab-like
CSO_011487	heat shock protein 67B2-like
**Hsp90**	
CSO_005276	endoplasmin-like, hsp90B1
CSO_007820	heat shock protein 75 kDa, mitochondrial-like
CSO_008013	Heat shock protein HSP 90-alpha
**Hsp110**	
CSO_001617	heat shock 70 kDa protein 4L-like

Table S16. Comparisons of the gene that may be involved in the development of eyes in five insect species.

**Gene symbol**	***D. melanogaster***	***C. solmsi***	***N. vitripennis***	***B. mori***	***D. plexippus***
Total number of genes	65	61		48	54
**Main retina**					
distal antenna	NP_651346	CSO_010287		BGIBMGA004818	DPGLEAN00077
distal antenna related	NP_651343	CSO_008825			
eyegone	NP_524042	CSO_002266	NP_001107659.1	BGIBMGA0009331	DPGLEAN15291
twin of eyegone	NP_524041	ND			
eyeless	NP_524628	CSO_004787	ACT79977.1	BGIBMGA009546	DPGLEAN02181
twin of eyeless	NP_524638	CSO_008996	ACT79981.1	BGIBMGA009432	DPGLEAN00908
eyes absent	NP_723188	CSO_009171	XP_001603597.2	BGIBMGA004453	DPGLEAN04095
sine oculis	NP_476733	CSO_011203		ND	DPGLEAN10223
dachshund	NP_723969	CSO_003392		BGIBMGA010389	DPGLEAN21309
optix	NP_524695	CSO_004007		BGIBMGA011285	DPGLEAN08309
teashirt	NP_524733	ND		BGIBMGA006678	DPGLEAN09888
tip-top	NP_523615	CSO_002414			
optix binding protein	NP_724479	CSO_009235		BGIBMGA008897	DPGLEAN08272
sine oculis binding protein	NP_610703	ND		ND	DPGLEAN12112
Microphtalmia associated transcription factor	NP_001033808	CSO_001601		ND	DPGLEAN10188
	NP_523977	CSO_007048	XP_001601600.2	BGIBMGA005390	DPGLEAN12225
hairy		CSO_002733	XP_001601817.1		
extramacrochaete	NP_523876	CSO_008241		BGIBMGA012124	DPGLEAN19645
daughterless	NP_477189	CSO_011275		BGIBMGA003285	DPGLEAN13328
atonal	NP_731223	CSO_009748		BGIBMGA013643	DPGLEAN02592
scabrous	NP_476710	CSO_002613		BGIBMGA000480	DPGLEAN18615
glass	NP_476854	CSO_000980		BGIBMGA002234	DPGLEAN06609
photoreceptor-cell-specific nuclear receptor	NP_611032	CSO_002136	XP_001606227.2	BGIBMGA013855	DPGLEAN05607
pebbled	NP_476674	CSO_007992		BGIBMGA004355	DPGLEAN02445
bunched	NP_525103	CSO_009522		BGIBMGA002705	DPGLEAN13699
rough	NP_524521	CSO_007101		ND	DPGLEAN10933
lozenge	NP_511099	CSO_006005	XP_001603414.2	BGIBMGA008907	DPGLEAN21068
runt	NP_523424	CSO_006006		BGIBMGA008906	DPGLEAN21067
big brother	NP_477065	CSO_010105	NP_001135856	BGIBMGA001342	DPGLEAN04490
		CSO_000009			
brother	NP_477066				
seven up	NP_524325	CSO_010231	NV18803	BGIBMGA001391	DPGLEAN20818
BarH1	NP_523387	CSO_000064		ND	DPGLEAN22302
BarH2	NP_523386				
BarH-like	NP_572815	CSO_000428	XP_001601247	BGIBMGA007623	DPGLEAN06193
sevenless	NP_511114	CSO_002068		BGIBMGA011535	DPGLEAN19817

bride of sevenless	NP_542440				
phyllopod	NP_725394	ND		ND	ND
seven in absentia	NP_476725	ND		BGIBMGA002878	DPGLEAN15848
anterior open	NP_722766	CSO_008308		BGIBMGA001167	DPGLEAN21233
shaven	NP_524633	CSO_002903	GQ301540.1	BGIBMGA013358	DPGLEAN12316
			ACT79980.1		
cut	NP_524764	CSO_000353	XP_001607026.2	BGIBMGA013198	DPGLEAN08613
tramtrack	NP_733443	CSO_010739		BGIBMGA007530	DPGLEAN19333
embryonic lethal,abnormal					
vision	NP_572842(fne)			BGIBMGA013823	DPGLEAN14282
	NP_525033(elav)	CSO_005419		BGIBMGA005888	DPGLEAN14614
	NP_476937(Rbp9)			BGIBMGA008284	DPGLEAN20097
drosocrystallin	NP_476906	ND		ND	ND
klingon	NP_524454	CSO_006585		BGIBMGA001703	DPGLEAN07987
	NP_524605	CSO_007432	XP_001603185	BGIBMGA006308	DPGLEAN18496
		CSO_006391	XP_001604537		
		CSO_006813	XP_001605598		
		CSO_006787	XP_001605659		
chaoptic		CSO_006923	XP_001606268		
soxN (sox neuro)	NP_524735	CSO_001257		BGIBMGA006156	DPGLEAN02459
	NP_524842	CSO_001191		BGIBMGA013353	DPGLEAN19875
onecut		CSO_001195			
prominin	NP_647770	CSO_001774	XP_001607729.2	BGIBMGA003937	DPGLEAN04982
		CSO_009524	XP_003425572.1		
			XP_003425573.1		
CG14955	NP_647769				
PvuIIPstI homology 13	NP_477330	ND		ND	ND
eyes shut	NP_001027571	CSO_000383		ND	DPGLEAN17109
warts	NP_733403	CSO_004903		BGIBMGA006330	DPGLEAN21306
melted	NP_523953	ND		BGIBMGA002897	DPGLEAN07797
**Drosal rim subset**					
spalt-major	NP_723670	CSO_000053		BGIBMGA002946	DPGLEAN03048
spalt-related	NP_523548	CSO_000054			
senseless	NP_524818	CSO_005631	XP_003426864.1	BGIBMGA003190	DPGLEAN22443
	NP_476578	CSO_005561		BGIBMGA004885	DPGLEAN20422
homothorax		CSO_002944		BGIBMGA011254	DPGLEAN17297
prospero	NP_731565	CSO_008650		BGIBMGA005083	DPGLEAN10138
orthodenticle (ocelliless)	NP_511091	CSO_002316	AAT94174.1	BGIBMGA008198	DPGLEAN08854
	NP_476748	CSO_008624		BGIBMGA010012	DPGLEAN16692
		CSO_011505			
spineless		CSO_011506			
araucan (iroC)	NP_524045	CSO_005343		BGIBMGA000637	DPGLEAN15162
caupolican (iroC)	NP_524046	CSO_005345			
mirror (iroC)	NP_524047	ND		BGIBMGA000639	DPGLEAN15164

Notes: *D. melanogaster*: *Drosophila melanogaster*; *C. solmsi*: *Ceratosolen solmsi*; *N. vitripennis*: *Nasonia vitripennis*; *B. mori*: *Bombyx mori*; *D. plexippus*: *Danaus plexippus*.

Table S17. Comparisons of the gene that may be involved in the development of wings in four insect species.

Gene symbol	*D. melanogaster*	*C. solmsi*	*N. vitripennis*	*A. pisum*
TOTAL	57	64	31	19
		CSO_009573	XP_001599660.2	XP_001946004.2
apterous	NP_724428.1	CSO_009575	XP_001599685.2	XP_001949543.1
armadillo	NP_476666.1	CSO_009259		
arrow	NP_524737.2	CSO_006876		
antennapedia	NP_996168.1	CSO_008709	NP_001161164.1	XP_001947187.1
baboon	NP_477000.1	CSO_010647		
bifid	NP_525070.2			
blistered	NP_726438.1	CSO_004431		
wing blister	ADV37056.1	CSO_003317		
brinker	NP_511069.3	CSO_000453		
		CSO_005098	XP_001600362.1	
		CSO_006713	XP_001603630.2	
crossveinless	NP_536786.1		XP_001607991.2	
cubitus interruptus	NP_524617.3	CSO_006900		
cut	NP_524764.1	CSO_000353		
	AAA97401.1	CSO_010723		
dally	NP_524071.2	CSO_002439	XP_001607767.2	
				XP_001945626.1
				XP_001944147.2
decapentaplegic	NP_477311.1	CSO_005640	XP_001607677.1	XP_001946010.2
delta	NP_477264.1	CSO_000975		
distal-less	NP_523857.1	CSO_001278		
		CSO_002426	XP_001607672.2	
engrailed	NP_523700.2	CSO_002427	XP_001607674.2	XP_001949185.2
escargot	NP_476600.1	CSO_000939		
fringe	NP_524191.1	CSO_005888		
hedgehog	NP_524459.2	CSO_009800		XP_001943707.1
Hipk	NP_612038.2	CSO_001909		
		CSO_005561	XP_001601467.2	
homothorax	NP_476578.3	CSO_002944	XP_003424016.1	XP_001951115.2
knirps	NP_524187.1	CSO_009849		
mastermind	NP_525115.1			
		CSO_000409	XP_003424076.1	
mindbomb	NP_648826.2	CSO_002708	XP_001606025.1	
		CSO_002763	XP_001601460.2	
mad	NP_477017.1	CSO_005251	XP_001602991.1	XP_001942613.2
medea	NP_524610.1	CSO_000830		
smox	NP_511079.1	CSO_002431	XP_001608214.2	
dad	NP_477260.1			

nemo	NP_729319.1	CSO_011004		
nipped-A	NP_001014499.2	CSO_000197		
		CSO_007561		
notch	NP_476859.2	CSO_007564	XP_001603128.2	XP_001948396.1
notum	NP_730096.2			
nubbin	NP_001097153.1	CSO_009962		XP_001944692.2
		CSO_001798		
		CSO_000193		
optomotorblind	NP_525070.2	CSO_010942	NV50208	XP_001945683.2
		CSO_010477		
patched	NP_523661.2	CSO_004234	NP_001123271.1	XP_001949597.2
punt	NP_731926.1	CSO_004874		
		CSO_011043	XP_001607800.2	
		CSO_006384	XP_001606093.2	
rhomboid	NP_523883.2	CSO_001642	XP_001602532.1	
saxophone	NP_523652.2	CSO_005058		
scalloped	NP_727900.1	CSO_004783		
serrate	NP_524527.3	CSO_002966		XP_001952607.2
		CSO_008712		
Sex-combs reduced	NP_524248.2	CSO_008711	NP_001128396.1	
smoothened	NP_523443.1	CSO_005235		
spalt-major	NP_723670.2	CSO_000053	NV17843	XP_001943388.2
spalt-related	NP_523548.1		XP_001601305.2	
		CSO_000072	XP_003424852.1	
suppressor of hairless	NP_476868.1	CSO_001516	XP_001600894.1	
tartan	NP_524055.2	CSO_007861	XP_003427009.1	
capricious	NP_524056.3			
target of wingless	NP_648061.1			
teashirt	NP_523615.2	CSO_002414		
tiptop	NP_524733.2	CSO_002414	XP_001600801.2	XP_001946631.2
thickveins	NP_787989.1	CSO_006232		
Ultrabithorax	NP_536752.1	CSO_008701		XP_001944459.2
ventral veins lacking	NP_523948.1	CSO_004410		
vestigial	NP_523723.1	CSO_010344	XP_001603271.2	
wingless	NP_523502.1	CSO_000946		XP_001945295.1

Notes: *D. melanogaster*: *Drosophila melanogaster*; *C. solmsi: Ceratosolen solmsi*; *N. vitripennis*: *Nasonia vitripennis*; *A. pisum*: *Acyrthosiphon pisum*.

Table S18. Comparison of the gene numbers in circadian rhythem system in different insect species.

Species	*timeless*	*timeout*	*period*	*cycle*	*clock*	*cwo*	*pdp*	*vri*	*cry1*	*cry2*	*pdf*
C. solmsi	0	2	1	1	1	1	1	1	0	1	1
A. mellifera	0	2	1	1	1	1	1	1	0	1	1
C. floridanus	0	2	1	1	1	1	1	1	0	1	1
H. saltator	0	2	1	1	1	1	1	1	0	1	1
N. vitripennis	0	2	1	1	1	2	1	1	0	1	1
D. plexippus	1	1	1	1	1	1	1	1	1	1	1
A. pisum	1	1	1	2	1	1	1	1	1	2	0
T. castaneum	1	1	1	1	1	1	1	1	0	1	0
D. pulex	10	1	1	1	1	1	1	1	1	1	1

Notes: *C. solmsi*: *Ceratosolen solmsi*; *A. mellifera*: *Apis mellifera*; *C. floridanus*: *Camponotus floridanus*; *H. saltator*: *Harpegnathos saltator*; *N. vitripennis*: *Nasonia vitripennis*; *D. plexippus*: *Danaus plexippus*; *A. pisum*: *Acyrthosiphon pisum*; *T. castaneum*: *Tribolium castaneum*; *D. pulex*: *Daphnia pulex*.

Table S19. Comparisons of the *yellow*-like genes among three hymenopteran insect species.

Gene name	Gene ID	Numbers (Am:Nv:Cs)	Members in Nv	Cs Position (Scaffold)	Nv Position (Scaff Id)	Notes
Cs-yellow-b	CSO_000634	1:1:1	Nv-yellow-b	1	5	
Cs-yellow-x2	CSO_001559	1:1:1	Nv-yellow-x2	10	21	unique in Hymenoptera
Cs-yellow-h	CSO_002345	1:1:1	Nv-yellow-h	11	42	
Cs-yellow-e3	CSO_002346	1:1:1	Nv-yellow-e3	11	42	originator of MRJPL gene
Cs-yellow-e	CSO_002347	1:1:1	Nv-yellow-e	11	42	
Cs-yellow-g2	CSO_002348	1:3:1	Nv-yellow-g2a,b,c	11	42	3 duplicates in *N.vitripennis*
Cs-yellow-g	CSO_002349	1:1:1	Nv-yellow-g	11	42	
Cs-yellow-x1	CSO_007152	1:5:1	Nv-yellow-x1a-e	4	62(a,b,c); 16(d,e)	unique in Hymenoptera; 5 duplicates in *N. vitripennis*
Cs-yellow	CSO_007840	1:1:1	Nv-yellow	45	36	
Cs-RJPL-1,2	CSO_009712 CSO_009713	0:10:2	Nv-RJPL-1-10	6	1011(1),143(3), 42(6)	unique in Chalcidoidea
Cs-yellow-f	CSO_010352	1:1:1	Nv-yellow-f	7	1395	unique in Hymenoptera

Notes: Am-*Apis mellifera*, Nv-*Nasonia vitripennis*, Cs-*Ceratosolen solmsi*.

Table S20. Manually annotated *hox* genes in fig wasp.

ID	Scaffold	Most similar gene description in NCBI
CSO_000064	scaffold1	Homeobox protein B-H1 (62%A)
CSO_000353	scaffold1	Homeobox protein cut [Harpegnathos saltator]
CSO_000397	scaffold1	EFN68751.1|Zinc finger protein 782 [Camponotus floridanus]
CSO_000428	scaffold1	homeobox protein GBX-1, putative (69%a) homeobox protein B-H1-like [Nasonia vitripennis]
CSO_000438	scaffold1	homeobox protein extradenticle-like isoform 2 [Nasonia vitripennis]
CSO_000673	scaffold1	segmentation protein even-skipped [Apis mellifera]
CSO_000996	scaffold10	Homeobox protein Nkx-2.5 (49%A)
CSO_011507	scaffold10	EFN79395.1 BarH-like 1 homeobox protein [Harpegnathos saltator]
CSO_001278	scaffold10	XP_001601872.2 homeotic protein distal-less-like [Nasonia vitripennis]
CSO_001289	scaffold10	XP_001602252.2 Homeobox protein GBX-1 (36%U)
CSO_001404	scaffold10	XP_001607545.1 homeobox protein MSX-1-like [Nasonia vitripennis]
CSO_001567	scaffold10	XP_001603153.1 insulin gene enhancer protein ISL-1-like [Nasonia vitripennis]
CSO_001648	scaffold10	XP_001606728.2|homeobox protein extradenticle-like [Nasonia vitripennis]
CSO_001651	scaffold10	XP_003393891.1|hypothetical protein LOC100642717 [Bombus terrestris]
CSO_001806	scaffold10	XP_001606402.2|protein Hook homolog 3-like [Nasonia vitripennis]
CSO_001988	scaffold11	XP_001605275.2| LIM/homeobox protein Lhx3-like isoform 1 [Nasonia vitripennis]
CSO_002266	scaffold11	NP_001107659.1|eyegone [Nasonia vitripennis]
CSO_002278	scaffold11	XP_001606147.2|Homeobox protein SIX4 (61%A)
CSO_002316	scaffold11	XP_003707995.1|uncharacterized protein LOC100879392 [Megachile rotundata]
CSO_002317	scaffold11	XP_003393134.1|homeobox protein otx5-B-like [Bombus terrestris]
CSO_002426	scaffold12	EFN84787.1|Segmentation polarity homeobox protein engrailed [Harpegnathos saltator]
CSO_002427	scaffold12	XP_001607672.2|Segmentation polarity homeobox protein engrailed (53%A)
CSO_002435	scaffold12	XP_003494022.1|homeobox protein ARX-like [Bombus impatiens]
CSO_002660	scaffold13	XP_003427431.1|Nk homeobox 7 (12%U)
CSO_002944	scaffold14	XP_001601467.2|homeobox protein homothorax-like [Nasonia vitripennis]
CSO_003114	scaffold14	XP_001605174.1|LIM homeobox transcription factor 1-beta-like [Nasonia vitripennis]
CSO_003599	scaffold17	XP_001600570.2|zinc finger homeobox protein 3-like [Nasonia vitripennis]
CSO_004007	scaffold2	XP_001607088.2|hypothetical protein LOC100123443 [Nasonia vitripennis]
CSO_004410	scaffold26	EFN69886.1|POU domain protein CF1A [Camponotus floridanus]
CSO_004436	scaffold26	NP_001153690.1|TGIF-like [Nasonia vitripennis]
CSO_004787	scaffold28	XP_001601318.2|paired box protein Pax-6 [Nasonia vitripennis]
CSO_005037	scaffold29	XP_001599133.1|hematopoietically-expressed homeobox protein HHEX-like [Bombus terrestris]
CSO_011510	scaffold29	XP_003690448.1|H2.0-like homeobox protein-like [Apis florea]
CSO_005314	scaffold29	XP_001599466.2|visual system homeobox 1 (69%A)
CSO_005343	scaffold29	XP_001604937.1|Homeobox protein araucan (70%A)
CSO_005345	scaffold29	XP_001604958.2|homeobox protein caupolican-like [Nasonia vitripennis]
CSO_005434	scaffold29	XP_003491882.1|LIM/homeobox protein Lhx3-like [Bombus impatiens]

CSO_005561	scaffold3	XP_003704976.1|homeobox protein PKNOX2-like [Megachile rotundata]
CSO_005565	scaffold3	XP_003399890.1|brain-specific homeobox protein homolog [Bombus terrestris]
CSO_005809	scaffold30	XP_001603519.2|Dorsal root ganglia homeobox protein (59%A)
CSO_005894	scaffold30	XP_001606575.2|homeobox protein orthopedia-like
CSO_005954	scaffold30	XP_001605968.2|inhibitory POU protein-like [Nasonia vitripennis]
CSO_006045	scaffold30	XP_003426540.1|Homeotic protein empty spiracles
CSO_006290	scaffold31	XP_003489458.1|homeobox protein ceh-8-like [Bombus impatiens]
CSO_006484	scaffold35	XP_001608004.1|Homeobox protein unc-4 (69%A)
CSO_006745	scaffold35	XP_003703488.1|homeobox protein Nkx-6.2-like [Megachile rotundata]
CSO_006865	scaffold39	NP_001128397.1|caudal [Nasonia vitripennis]
CSO_007101	scaffold4	XP_003425474.1|homeobox protein rough-like [Nasonia vitripennis]
CSO_007494	scaffold43	XP_003393515.1|retinal homeobox protein Rx-like [Bombus terrestris]
CSO_007495	scaffold43	EGI57540.1|Homeobox protein ARX [Acromyrmex echinatior]
CSO_007553	scaffold43	XP_001602803.2|diencephalon/mesencephalon homeobox protein 1-like, partial [Nasonia vitripennis]
CSO_008395	scaffold48	XP_002431383.1|POU domain protein C, putative [Pediculus humanus corporis]
CSO_008451	scaffold49	XP_001599246.2|paired box protein Pax-3-B
CSO_008494	scaffold5	XP_001601152.2|LIM/homeobox protein Lhx5-like [Nasonia vitripennis]
CSO_008600	scaffold53	XP_003489795.1| homeobox protein aristaless-like 4-like isoform 2 [Bombus impatiens]
CSO_008650	scaffold56	NP_001164363.1|homeobox protein prospero [Nasonia vitripennis]
CSO_008689	scaffold56	XP_001603594.2|Homeobox protein abdominal-B (45%U) (abd-B)
CSO_008699	scaffold56	XP_003402189.1|homeobox protein abdominal-A homolog isoform 1 [Bombus terrestris] (abd-A)
CSO_008701	scaffold56	NP_001162171.1|ultrabithorax [Apis mellifera] (ubx)
CSO_008709	scaffold56	NP_001161164.1|antennapedia-like protein [Nasonia vitripennis] (antp)
CSO_008711	scaffold56	XP_001603670.1|Fushi-tarazu protein (Fragment), putative (ftz)
CSO_008712	scaffold56	NP_001128396.1|sex combs reduced [Nasonia vitripennis] (scr)
CSO_008715	scaffold56	XP_003700437.1|Homeotic protein deformed (dfd)
CSO_008716	scaffold56	XP_001603758.2|Homeobox protein, putative (11%U) (hox3)
CSO_008718	scaffold56	EGI64564.1|Homeotic protein proboscipedia [Acromyrmex echinatior] (pb)
CSO_008720	scaffold56	XP_001603839.2|homeobox protein Hox-B1-like [Nasonia vitripennis] (lab)
CSO_008854	scaffold56	XP_001606522.2|Short stature homeobox protein (59%A)
CSO_008938	scaffold56	XP_001599629.1|LIM/homeobox protein Lhx9 (56%A)
CSO_008996	scaffold57	XP_001602823.2|paired box protein Pax-6 [Nasonia vitripennis]
CSO_009134	scaffold57	XP_003693389.1|paired mesoderm homeobox protein 2B-like [Apis florea]
CSO_009305	scaffold6	EFN87697.1|Homeobox protein not2 [Harpegnathos saltator]
CSO_009553	scaffold6	XP_001601499.1|Retinal homeobox protein Rx2 (67%A)
CSO_009573	scaffold6	XP_001599685.2| LIM/homeobox protein Lhx9-like [Nasonia vitripennis]
CSO_009575	scaffold6	XP_001599660.2|apterous, protein (66%A)
CSO_009602	scaffold6	XP_001601511.1|nk homeobox protein
CSO_009603	scaffold6	XP_001601482.2|homeobox protein HMX3
CSO_009698	scaffold6	XP_003696436.1|homeobox protein Nkx-2.1-like [Apis florea]
CSO_011513	scaffold6	XP_003400164.1|PREDICTED: homeobox protein Hox-B3-like

CSO_009894	scaffold6	XP_003701047.1|Homeobox protein prophet of Pit-1
CSO_009962	scaffold61	XP_003394939.1| PREDICTED: protein nubbin-like
CSO_009976	scaffold61	XP_001603592.1|pituitary homeobox homolog Ptx1-like [Nasonia vitripennis]
CSO_010195	scaffold63	XP_001119904.1|t-cell leukemia homeobox protein 3-like [Apis mellifera]
CSO_010199	scaffold63	EGI63977.1|Transcription factor LBX1 [Acromyrmex echinatior]
CSO_010200	scaffold63	XP_003427887.1|homeobox protein Nkx-2.5-like [Nasonia vitripennis]
CSO_010201	scaffold63	XP_001600859.1|homeobox protein Nkx-2.6-like [Nasonia vitripennis]
CSO_010203	scaffold63	XP_001601013.2|Muscle segmentation homeobox (43%U)
CSO_010204	scaffold63	XP_003703878.1|homeobox protein H17
CSO_010990	scaffold75	XP_001601137.2|LIM/homeobox protein Awh-like [Nasonia vitripennis]
CSO_011203	scaffold87	XP_001600428.2|Homeobox protein SIX1 (75%A)
CSO_011353	scaffold94	XP_001599456.2|paired mesoderm homeobox protein 2-like [Nasonia vitripennis]

Table S21. Annotation of the homologous genes implicated in sex determination pathways in *Ceratosolen solmsi*.

**Gene**	***C. solmsi***
complementary sex determiner	--
feminizer	CSO_000025
	CSO_011499
doublesex	CSO_010597
transformer-2	CSO_006852
fruitless	CSO_001447
dissatisfaction	CSO_002136
extra-marcochaetae	CSO_008241
groucho	CSO_010842
	CSO_010836
deadpan	CSO_007058
female lethal(2)d	CSO_000800
virilizer	CSO_011500
daughterless	CSO_011275
intersex	CSO_007971
runt	CSO_006006
hopscotch	CSO_002323
sex lethal	CSO_006311

Table S22. Fig wasp specific genes are significantly devoid of putatively methylated genes compared to orthologous genes.

Number of Genes	Orthologous Genes	Fig Wasp Specific Genes
Putatively Methylated Genes	1320	274
Putatively Non-methylated Genes	6766	3040
		P < 10-16 (Fisher’s exact test)

Supplementary reference

1. Henderson B, Allan E, Coates ARM: **Stress wars: the direct role of host and bacterial molecular chaperones in bacterial infection.** *Infect Immun* 2006, **74:**3693-3706.
2. Pockley AG: **Heat shock proteins as regulators of the immune response.** *The Lancet* 2003, **362:**469-476.
3. Zhan S, Merlin C, Boore Jeffrey L, Reppert Steven M: **The monarch butterfly genome yields insights into long-distance migration.** *Cell* 2011, **147:**1171-1185.
4. Kaminker JS, Singh R, Lebestky T, Yan H, Banerjee U: **Redundant function of *Runt* Domain binding partners, *Big brother* and *Brother*, during *Drosophila* development.** *Development* 2001, **128:**2639-2648.
5. Reinke R, Krantz DE, Yen D, Lawrence Zipursky S: **Chaoptin, a cell surface glycoprotein required for *Drosophila* photoreceptor cell morphogenesis, contains a repeat motif found in yeast and human.** *Cell* 1988, **52:**291-301.
6. Zelhof AC, Hardy RW, Becker A, Zuker CS: **Transforming the architecture of compound eyes.** *Nature* 2006, **443:**696-699.
7. Allada R, Chung BY: **Circadian organization of behavior and physiology in *Drosophila*.** *Annu Rev Physiol* 2010, **72:**605-624.
8. Kaushik R, Nawathean P, Busza A, Murad A, Emery P, Rosbash M: **PER-TIM interactions with the photoreceptor cryptochrome mediate circadian temperature responses in *Drosophila*.** *PloS Biol* 2007, **5:**e146.
9. Wang B, Xiao J-H, Bian S-N, Niu L-M, Murphy RW, Huang D-W: **Evolution and expression plasticity of opsin genes in a fig pollinator, *Ceratosolen solmsi*.** *PLoS ONE* 2013, **8:**e53907.
10. Reppert SM: **A colorful model of the circadian clock.** *Cell* 2006, **124:**233-236.
11. Maleszka R, Kucharski R: **Analysis of *Drosophila* yellow-B cDNA reveals a new family of proteins related to the royal jelly proteins in the honeybee and to an orphan protein in an unusual bacterium *Deinococcus radiodurans*.** *Biochem Biophys Res Commun* 2000, **270:**773-776.
12. Drapeau MD, Albert S, Kucharski R, Prusko C, Maleszka R: **Evolution of the Yellow/Major Royal Jelly Protein family and the emergence of social behavior in honey bees.** *Genome Res* 2006, **16:**1385-1394.
13. Werren JH, Richards S, Desjardins CA, Niehuis O, Gadau J, Colbourne JK, Group TNGW: **Functional and evolutionary insights from the genomes of three parasitoid *Nasonia* species.** *Science* 2010, **327:**343-348.
14. Suen G, Teiling C, Li L, Holt C, Abouheif E, Bornberg-Bauer E, Bouffard P, Caldera EJ, Cash E, Cavanaugh A, et al: **The genome sequence of the leaf-cutter ant *Atta cephalotes* reveals insights into its obligate symbiotic lifestyle.** *PLoS Genet* 2011, **7:**e1002007.
15. Carroll SB, Grenier JK, Weatherbee SD: *From DNA to diversity: molecular genetics and the evolution of animal design, 2nd edition.* Oxford, UK: Wiley-Blackwell Publishing Ltd; 2004.
16. Lewis EB, Pfeiffer BD, Mathog DR, Celniker SE: **Evolution of the homeobox complex in the Diptera.** *Curr Biol* 2003, **13:**R587-R588.
17. Dearden PK, Wilson MJ, Sablan L, Osborne PW, Havler M, McNaughton E, Kimura K, Milshina NV, Hasselmann M, Gempe T, et al: **Patterns of conservation and change in honey bee developmental genes.** *Genome Res* 2006, **16:**1376-1384.
18. Pearson JC, Lemons D, McGinnis W: **Modulating Hox gene functions during animal body patterning.** *Nat Rev Genet* 2005, **6:**893-904.
19. Cook JM: **Sex determination in the Hymenoptera: a review of models and evidence.** *Heredity* 1993, **71:**421-435.
20. Schmieder S, Colinet D, Poirié M: **Tracing back the nascence of a new sex-determination pathway to the ancestor of bees and ants.** *Nat Commun* 2012, **3:**895.

21. Verhulst EC, Beukeboom LW, van de Zande L: **Maternal control of haplodiploid sex determination in the wasp *Nasonia*.** *Science* 2010, **328:**620-623.
22. Glastad KM, Hunt BG, Yi SV, Goodisman MAD: **DNA methylation in insects: on the brink of the epigenomic era.** *Insect Mol Biol* 2011, **20:**553-565.
23. Jurkowski TP, Meusburger M, Phalke S, Helm M, Nellen W, Reuter G, Jeltsch A: **Human DNMT2 methylates tRNA (Asp) molecules using a DNA methyltransferase-like catalytic mechanism.** *RNA* 2008, **14:**1663-1670.
24. Bird AP: **DNA methylation and the frequency of CpG in animal DNA.** *Nucleic Acids Res* 1980, **8:**1499-1504.
25. Elango N, Yi SV: **DNA methylation and structural and functional bimodality of vertebrate promoters.** *Mol Biol Evol* 2008, **25:**1602-1608.
26. Honeybee Genome Sequencing Consortium: **Insights into social insects from the genome of the honeybee *Apis mellifera*.** *Nature* 2006, **443:**931-949.
27. Wang Y, Jorda M, Jones PL, Maleszka R, Ling X, Robertson HM, Mizzen CA, Peinado MA, Robinson GE: **Functional CpG methylation system in a social insect.** *Science* 2006, **314:**645-647.
28. Park J, Peng Z, Zeng J, Elango N, Park T, Wheeler D, Werren JH, Yi SV: **Comparative analyses of DNA methylation and sequence evolution using *Nasonia* genomes.** *Mol Biol Evol* 2011, **28:**3345-3354.
29. Sarda S, Zeng J, Hunt BG, Yi SV: **The evolution of invertebrate gene body methylation.** *Mol Biol Evol* 2012, **29:**1907-1916.
30. The International Aphid Genomics Consortium: **Genome sequence of the pea aphid *Acyrthosiphon pisum*.** *PloS Biol* 2010, **8:**e1000313.
31. Martinson E, Herre E, Machado C, Arnold AE: **Culture-free survey reveals diverse and distinctive fungal communities associated with developing figs (*Ficus* spp.) in Panama.** *Microb Ecol* 2012, **64:**1073-1084.
32. Smith CR, Smith CD, Robertson HM, Helmkampf M, Zimin A, Yandell M, Holt C, Hu H, Abouheif E, Benton R, et al: **Draft genome of the red harvester ant *Pogonomyrmex barbatus*.** *Proc Natl Acad Sci USA* 2011, **108:**5667-5672.
33. Bonasio R, Zhang G, Ye C, Mutti NS, Fang X, Qin N, Donahue G, Yang P, Li Q, Li C, et al: **Genomic comparison of the ants *Camponotus floridanus* and *Harpegnathos saltator*.** *Science* 2010, **329:**1068-1071.
34. Smith CD, Zimin A, Holt C, Abouheif E, Benton R, Cash E, Croset V, Currie CR, Elhaik E, Elsik CG, et al: **Draft genome of the globally widespread and invasive Argentine ant (*Linepithema humile*).** *Proc Natl Acad Sci USA* 2011, **108:**5673-5678.
35. Nygaard S, Zhang G, Schiott M, Li C, Wurm Y, Hu H, Zhou J, Ji L, Qiu F, Rasmussen M, et al: **The genome of the leaf-cutting ant *Acromyrmex echinatior* suggests key adaptations to advanced social life and fungus farming.** *Genome Res* 2011, **21:**1339-1348.
36. Yu Q, Lu C, Li B, Fang S, Zuo W, Dai F, Zhang Z, Xiang Z: **Identification, genomic organization and expression pattern of glutathione S-transferase in the silkworm, *Bombyx mori*.** *Insect Biochem Mol Biol* 2008, **38:**1158-1164.
37. Oakeshott JG, Johnson RM, Berenbaum MR, Ranson H, Cristino AS, Claudianos C: **Metabolic enzymes associated with xenobiotic and chemosensory responses in *Nasonia vitripennis*.** *Insect Mol Biol* 2010, **19:**147-163.
38. Ramsey JS, Rider DS, Walsh TK, De Vos M, Gordon KHJ, Ponnala L, Macmil SL, Roe BA, Jander G: **Comparative analysis of detoxification enzymes in *Acyrthosiphon pisum* and *Myzus persicae*.** *Insect Mol Biol* 2010, **19:**155-164.
39. Tsubota T, Shiotsuki T: **Genomic analysis of carboxyl/cholinesterase genes in the silkworm *Bombyx mori*.** *BMC Genomics* 2010, **11:**377.
40. Grbic M, Van Leeuwen T, Clark RM, Rombauts S, Rouze P, Grbic V, Osborne EJ, Dermauw W, Thi Ngoc PC, Ortego F, et al: **The genome of *Tetranychus urticae* reveals herbivorous pest adaptations.** *Nature* 2011, **479:**487-492.
41. Shi H, Pei L, Gu S, Zhu S, Wang Y, Zhang Y, Li B: **Glutathione S-transferase (GST) genes in the red flour beetle, *Tribolium castaneum*, and comparative analysis with five additional insects.** *Genomics* 2012, **100:**327-335.

42. Zhu F, Moural T, Shah K, Palli S: **Integrated analysis of cytochrome P450 gene superfamily in the red flour beetle, *Tribolium castaneum*.** *BMC Genomics* 2013, **14:**174.
43. Gerardo N, Altincicek B, Anselme C, Atamian H, Barribeau S, de Vos M, Duncan E, Evans J, Gabaldon T, Ghanim M, et al: **Immunity and other defenses in pea aphids, *Acyrthosiphon pisum*.** *Genome Biol* 2010, **11:**R21.
44. Meisel RP, Malone JH, Clark AG: **Disentangling the relationship between sex-biased gene expression and X-linkage.** *Genome Res* 2012, **22:**1255-1265.
